# Life on Green Patches: Diversity and Seasonal Changes of Butterfly Communities Associated With Wastelands of the Post‐Industrial Central European City

**DOI:** 10.1002/ece3.70695

**Published:** 2024-12-16

**Authors:** Sylwia Pietrzak, Krzysztof Pabis

**Affiliations:** ^1^ Department of Invertebrate Zoology and Hydrobiology University of Lodz Łódź Poland

**Keywords:** diversity, habitat fragmentation, lepidoptera, ruderal vegetation, urbanisation

## Abstract

Urban wastelands are among the most neglected urban habitats. Our study demonstrated that those spatially restricted patches of vegetation are an important refuge for various species of butterflies. We have assessed the diversity, distribution patterns, and seasonal changes of butterfly communities based on two‐year (2019–2020), quantitative studies at 5 urban wastelands in a large post‐industrial city in Central Poland. Forty‐six species of butterflies were recorded in the city. We have noticed homogeneity of fauna, although all investigated sites were characterised by high diversity and co‐occurrence of species associated with different habitats (e.g., grasslands, woodlands). Most of the species were common in Central Poland, although we have also recorded the presence of more specialised butterflies. Bray–Curtis similarity analysis reflected mostly seasonal changes in species composition. Seasonal patterns were very similar at all investigated sites and during both seasons, pointing to relative stability. Urban wastelands hosted from 34 to 41 species. This pattern results from the high diversity of microhabitats and the co‐occurrence of various plant species at single sites, which is very important for plant‐dependent organisms like butterflies.

## Graphical abstract

We have assessed diversity, distribution patterns and seasonal changes of butterfly communities based on 2 year quantitative studies at 5 urban wastelands located in large postindustrial city in the Central Poland. Urban wastelands are small scale biodiversity host spots for butterfly fauna, that stand out from species poor butterfly communities associated with agricultural landscape typical for areas surrounding the city as a result of habitat heterogeneity and plant diversity. Our study is a first quantitative analysis of urban butterfly communities in Central and Eastern Europe and one of the few similar studies in general.

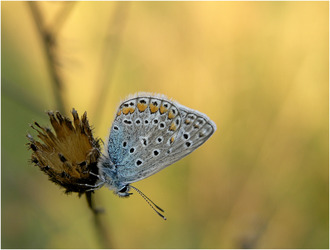

## 1 Introduction

Cities are complicated systems characterised by unique functioning mechanisms, and exceptional evolutionary pathways (Diamond and Martin 2021). The dynamic character, unstable conditions, and variety of disturbance agents make them great natural laboratories which may allow us to answer more general and very interesting questions associated with ecology and evolution, particularly those related to habitat loss, connectivity, disturbance, recovery processes, or resilience to changes (Parris 2018; Diamond and Martin 2021). It is especially evident in the context of the growing number of threats associated with climate warming, which are resulting in horizontal and vertical range extensions (Konvicka et al. 2003; Chen et al. 2011), changes in phenology and development time (MacLean, Kingsolver, and Buckley 2016), or modifications of life histories (Magura et al. 2021). Urban heat islands might be interesting small‐scale model sites for such studies, allowing for the analysis of ecosystem resilience or thermal regimes of particular species facing temperature changes similar to what has already been demonstrated for ants (Angilletta Jr et al. 2007).

Half of the global human population currently lives in urban areas, therefore, cities have become the closest ecosystems we humans interact with (Ritchie and Roser 2018). Growing attention to biodiversity resulted in increased interest in studies of urban ecosystems (Pimm and Raven 2000; Sánchez‐Bayo and Wyckhuys 2019). Cities are unique fragmented areas consisting of various habitat patches of different quality (Alberti 2005). Our common notion of urban habitat is usually an urban green space, mainly intentionally designed, like parks and lawns or some relicts of natural or semi‐natural vegetation, like urban forests, that are often protected as nature reserves (Konvicka and Kadlec 2011, Nielsen et al. 2014, Fontaine et al. 2016, Han et al. 2022, Płóciennik et al. 2023). The importance of the above‐mentioned habitats is undoubtedly relevant, but other urban habitats are still neglected in modern studies. A good example of such areas are patches of ruderal vegetation and various wastelands that may act as ecological corridors (Oki et al. 2021; Zellmer and Goto 2022) and create a living space and resources for butterflies and other insects associated with open habitats (Karlsson and Wiklund 2004; Twerd, Sobieraj‐Betlińska, and Szefer 2021). Wastelands are defined as areas where spontaneous vegetation takes over and is mainly or completely left without implementing maintenance. Therefore, these wastelands become a small‐scale hot spot of resources for local wildlife (Qviström 2008; Bonthoux et al. 2014; Sanches and Pellegrino 2016).

Butterflies are often mentioned as perfect indicators of urban disturbance (Blair 1999; Dennis et al. 2017; Tzortzakaki et al. 2019). They are functionally diverse, easy to identify, and many species are sensitive to changes, disturbance processes, and pollution events (Blair 1999; Meléndez‐Jaramillo et al. 2021; Kozlov et al. 2022). Moreover, butterflies are sometimes treated as good surrogates for general urban biodiversity assessment (Dollar et al. 2014). Nevertheless, the number of studies dedicated to urban butterfly communities is still relatively scarce, even in densely populated areas in Europe (Ramirez‐Restrepo and MacGregor‐Fors 2017). A large number of studies have focused on simple urbanisation gradients, demonstrating the relatively obvious pattern of declining diversity towards the city centre (e.g., Blair and Launer 1997; Blair 1999; Matsumoto 2015; Sobczyk, Pabis, and Wieczorek 2018; Tzortzakaki et al. 2019), or contained only the species lists (Ramirez‐Restrepo and MacGregor‐Fors 2017). We especially lack studies of resource‐based approaches, including host‐plant interactions, floral resources availability, diversity‐microhabitat relations (Dennis, Shreeve, and Van Dyck 2006), and temporal trends based on quantitative data (Ramirez‐Restrepo and MacGregor‐Fors 2017). There are also large spatial gaps in urban butterfly studies, especially in tropical areas, but also in Central and Eastern Europe (Ramirez‐Restrepo and MacGregor‐Fors 2017), an area that is facing an increasing level of urbanisation (Restrepo Cadavid et al. 2017) and numerous threats associated with climate change (Engelhardt et al. 2022) an aspect of great importance for urban areas (McCarthy and Sanderson 2011).

These facts are surprising, especially when we realise that Europe is facing a substantial decline in insect abundance, most comprehensively documented in protected areas (Hallmann et al. 2017), but certainly visible in other disturbed and modified urban or agricultural habitats (Moller 2020). Moreover, recent studies have demonstrated the decline of European butterfly abundance and diversity, with numerous local extinction events (Warren et al. 2021), and it is becoming evident that butterflies are declining faster in these urban areas (Dennis et al. 2017). Urbanisation is listed among the most important causes of insect decline on a global scale (Fenoglio et al. 2021). At the same time, there are almost no ecological analyses of urban butterfly communities in Central Europe. The only studies from Poland contain raw species lists (Machnikowski 1999; Winiarska 2003; Palik et al. 2005; Sielezniew and Dziekańska 2019) or are focused on urbanisation gradient (Senn 2015; Sobczyk, Pabis, and Wieczorek 2018). There are also a few studies from the Czech Republic, but all were focused on Prague and analysed nature reserves and large parks (Kadlec et al. 2008; Konvicka and Kadlec 2011).

Wastelands are important for understanding diversity patterns and ecological processes in cities and, as such, should be among the main focuses for not just ecologists but also authorities, especially taking into account the fact that those fragmented areas are vulnerable to intensive management practises (Aguilera et al. 2019). It has also been proven that urban green spaces increase the well‐being of citizens, something that is much needed in the current times (Sanches and Pellegrino 2016; Ma et al. 2019; Huma, Lin, and Hyder 2021; Reyes‐Riveros et al. 2021). Nevertheless, sustainable management strategies require comprehensive baseline knowledge. There are no reference point datasets for Central European urban butterfly communities that could serve as a baseline for further temporal studies during times of global change. Therefore, our study aims to analyse seasonal changes in butterfly diversity associated with fragmented ruderal sites located in a large city (Łódź, Central Poland) in relation to habitat characteristics, based on a two‐year, quantitative approach. We hypothesise that urban wastelands are important habitats for ecologically and taxonomically diverse butterfly fauna.

## 2 Materials and Methods

### 2.1 Study Area

Łódź is the fourth largest city in Poland, both in terms of surface area and number of citizens. It is located on an upland in the central part of the country. The current area of the city reaches nearly 300 km^2^.Łódź is inhabited by about 660,000 citizens. It is spatially connected with smaller urbanised areas, which extend the urbanised surface area by about 40%.

**FIGURE 1:**
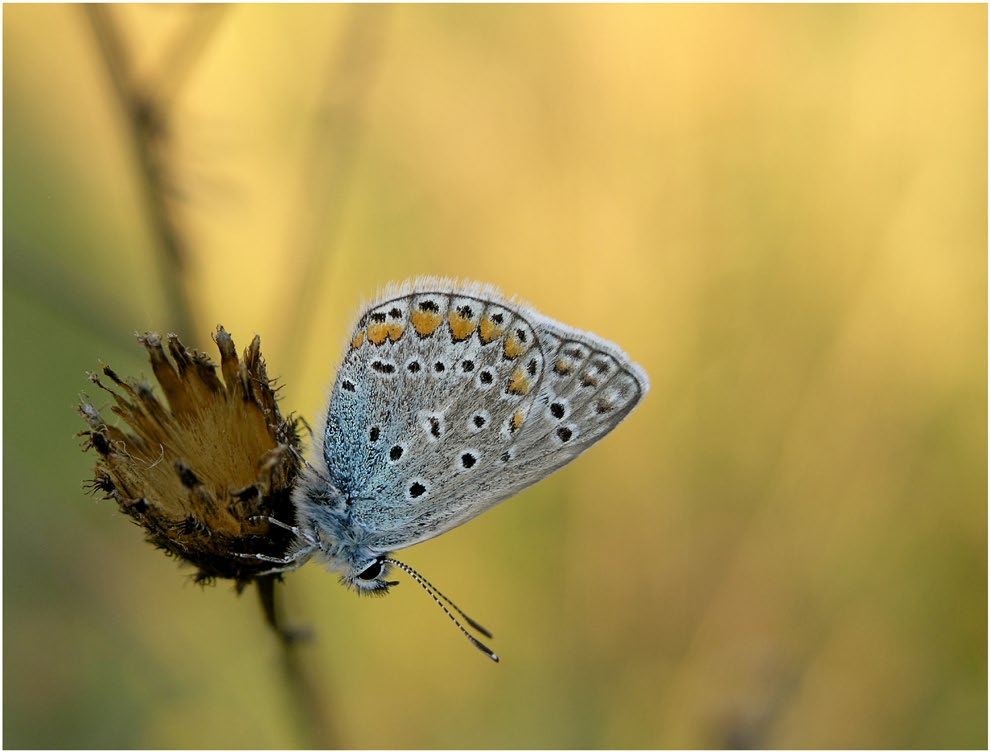
*Polyommatus icarus* is one the most common species in Łódź (photo by Krzysztof Pabis).

The large agglomeration developed rapidly as a result of textile manufacturing development in a few decades, starting from the beginning of the XIX century. Vast forests of various kinds and marshlands initially covered the area of mostly flat relief crossed by a network of small rivers and streams (Tranda et al. 1983; Markowski et al. 1998; Witosławski 2006). As the city grew, most of the rivers crossing the area were hidden underground and were eventually transformed into sewage canals. Unlike some older cities often organised along large river banks, Łódź is more uniformly and densely built up and is not fragmented by riverbeds that might bring opportunities for some species dispersal. Even the smallest species, like *Polyommatus icarus*, one of the most common butterflies in Łódź (Figure 1), might benefit from wastelands and other green spaces that may serve as ecological corridors or refuge.

**FIGURE 2:**
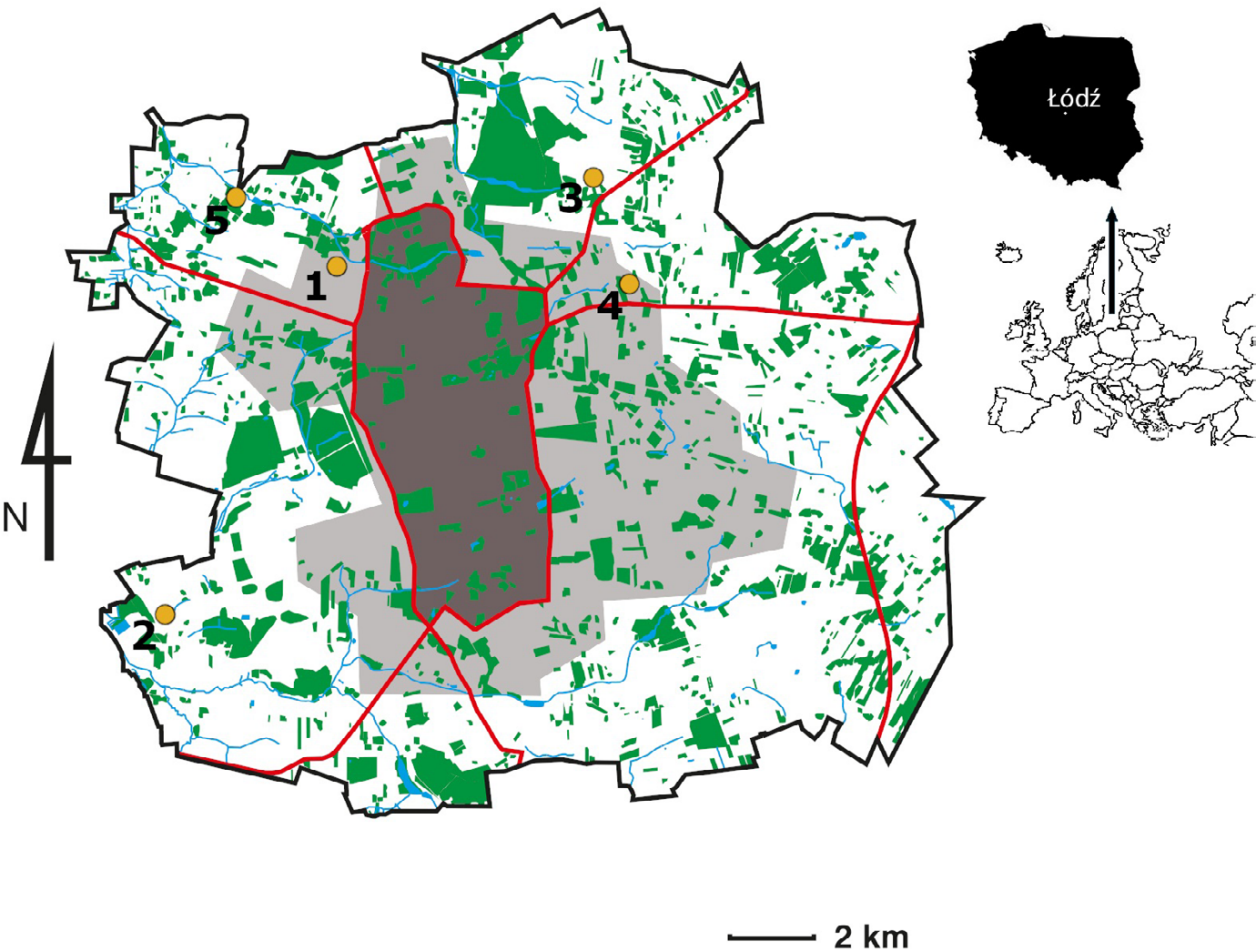
Distribution of sampling sites in the city. Major parks, squares and gardens are indicated. Borders of urbanisation zones are also marked using dark grey, light grey and white color. Red lines indicate the main streets of the city. (1) Brukowa, (2) Maratonska, (3) Rogi, (4) Telefoniczna, (5) Traktorowa.

Three urbanisation zones (Figure 2) were distinguished in Łódź: inner city, peri‐urban area, and outskirts (Witosławski 2006). The inner city (zone I) is characterised by the highest impermeable ground coverage and tightly arranged buildings, with green spaces mostly restricted to lawns, parks, and cemeteries (usually on the border of inner and peri‐urban areas). Residential estates, small houses, and some industrial facilities dominate in the peri‐urban area (zone II). There are more green spaces like parks, gardens, and wastelands than are found in the city centre. Outskirts (zone III) have a loose building arrangement, and there are some agricultural lands, wastelands, meadows, and semi‐natural and ruderal habitats. This zone is also characterised by the presence of the large forest complex (Łagiewniki Forest—1200 ha) located in the northeast part of the city (Witosławski 2006).

### 2.2 Field Studies

Five research sites were distinguished based mostly on their large size and type of habitat (Figure 1). They represented wasteland habitats located in the peri‐urban area (zone II) and outskirts (zone III), while there are no similar habitats in the most densely urbanised zone I. All the sites remained the wasteland character for more than 20 years. Butterfly counts followed a widely‐used monitoring scheme proposed by Pollard (1977) modified from an originally introduced 1100 m distance to match the small size of the urban research sites, which is common practise in similar studies (Mattoni et al. 2001; Clark, Reed, and Chew 2007; Aguirre‐Gutiérrez et al. 2017; Tzortzakaki et al. 2019). All transects were visited weekly for 26 weeks from April to September of 2019 and 2020 unless rain or temperatures below 13°C occurred (only a few such exceptions occurred in April of both years). Forms for transect description included information about temperature, humidity, wind conditions, and cloud cover. Weather‐related factors were obtained right before starting transect walks with actual weather information provided through the service https://weather.com/ or assessed by observation (for cloud cover). Identification was based on guides for Polish and European fauna (Buszko and Masłowski 2008; Sielezniew and Dziekańska 2010; Tolman 1997). Altogether, 214 Pollard walks (a single walk was treated as a quantitative sample) were conducted, including 109 in 2019 and 105 in 2020. The number of Pollard walks on each site equalled 22 in 2019 and 21 in 2020 at Brukowa, 24 in 2019 and 21 in 2020 at Maratońska, 22 in 2019 and 20 in 2020 at Rogi, 22 in 2019 and 21 in 2020, 19 in 2019 and 22 in 2020 at Traktorowa.

Data about the species composition of flowering plants was also collected for each site regularly throughout the whole sampling season. Identification was based on keys and field guides along with a distribution atlas dedicated to the flora of Łódź (Rutkowski 1998; Witosławski 2006; Sudnik‐Wójcikowska 2020). This dataset was used for the description of sampling sites and the list of species is included in Appendix S1. Detailed descriptions of sites are provided in Appendix S2.

Site on Brukowa (B) had the size of about 2 ha and was located in a predominantly industrial district, in close vicinity to the Zabieniec train station (peri‐urban zone of Łódź). Transect was 700 m long. Maratonska (M) was situated in a close proximity to the western city border, near the sewage treatment facility in the outskirts zone and had size of about 2 ha (Figure 1). The transect for this site was 600 m long. The site on Rogi (R) was situated on the border between the peri‐urban area and outskirt zone with Lagiewniki forest nearby (Figure 2). It was about 3 ha and the transect was 700 m long. The Telefoniczna (TL) site was situated in the peri‐urban zone (Figure 2). The area (about 3 ha) was neighbouring a residential area, tramway depot, magazines and workshop. Transect was 700 m long. The Traktorowa (TR) was the outskirt site situated in Sokolowka river valley (size of about 2 ha), between a riverbed and a residential area (Figure 1). The transect here was 500 m long. Wastelands were not moved during the studied period, except of the site on Maratonska which was only partially moved once in the summer of 2020, therefore this fact did not affected butterfly communities.

### 2.3 Data Analysis

The sample was defined as one transect count providing data about the number of species and individuals. Since transects had different lengths in order to cover the whole spectrum of vegetation types at each site and depending on specific characteristics of the sites, the number of individuals was calculated for the 500 m length (the size of the shortest transect) in order to analyse fully comparable samples. Those values were used in all further analysis.

Diversity analysis was performed in the Primer 5.0 package (Clarke and Warwick 2001). Number of species per sample (S), Margalef Index, Shannon Index (log e), and Pielou evenness were calculated for each sample (Magurran 2004). Taxonomic diversity Delta (Δ) and taxonomic distinctness Delta⁎ (Δ⁎) were also calculated to provide a view of the phylogenetic diversity at each site based on Linnaean ranks, namely: species, genus, tribe, subfamily, family, and superfamily (Appendix S3). Analysis was performed in Primer 5.0 package (Clarke and Warwick 2001) and based on formulas proposed by Warwick and Clarke (1995) as well as Clarke and Warwick (2001).

Mean values of total butterfly abundance and all above‐mentioned indices with standard deviation (SD) were calculated for each site. The statistical significance of differences between studied sites was analysed using appropriate statistical tests in the Statistical 6 package. Normal distribution was checked using Shapiro–Wilk's test, and homogeneity of variance was assessed using Levene's test. As a result, a non‐parametric Kruskall–Wallis test was used. Post hoc testing was done using Dunn's test. Differences between the seasons at each site were assessed using *t*‐test and Man–Whitney *U* test.

Bray–Curtis similarity was used in order to analyse distribution patterns and similarities between analysed samples. Hierarchical agglomerative clustering was performed using a group−average method. Data were square root transformed to minimise the influence of dominant species on the results of analysis (Clarke and Warwick 2001). Samples were coded with a combination of information about the year, observation week, site abbreviation, and sample number (i.e., 2019_02_B_01—first sample derived from Brukowa site during the second week of the season 2019). SIMPROF test with a 5% significance level was performed to check the multivariate structure within groups and a SIMPER analysis, which allowed the selection of the species most important for dendrogram division (Clarke and Gorley 2001), was also performed. This part of the analysis was performed using a Primer 7 package.

Additionally, frequency of occurrence [%] defined as the percentage of samples where a given species was found in the total number of samples at a particular site or particular cluster was calculated. Maximum and mean abundance with standard deviation was calculated for each species in each cluster of samples and for each site. For every species, the association index DAI (the percentage of individuals of a given species recorded in a given cluster group/site, within the total number of individuals of that species in the study area) was used. The DAS association index (the percentage of samples containing individuals of a given species in a given cluster group/site within the total number of samples containing that species in the study area) was also calculated (Salzwedel, Rachor, and Gerdes 1985; Siciński 2000).

Data on total butterfly abundance, species richness, and species composition was also visualised on a background of temperature, humidity, and other weather conditions data throughout the whole season, separately for 2019 and 2020 demonstrating phenological dynamics of butterfly communities at each site.

## 3 Results

### 3.1 Species Composition of Fauna

Altogether, 46 species (7880 individuals) were recorded at 5 investigated sites during both seasons. Species represented all five butterfly families. The most speciose were Nymphalidae (20 species), followed by Lycaenidae (12 species), Pieridae (8 species), Hesperiidae (5 species), and Papilionidae (1 species). The species with the highest frequency of occurrence in all samples were: *Coenonympha pamphilus* (*F* = 67%), *Pieris rapae* (*F* = 65%), and *Pieris napi* (*F* = 57%) (Appendix S4).

The species composition was similar for all investigated sites, demonstrating a homogenous character of fauna. Twenty‐five species occurred at all five sites, 6 species were found at four sites, 5 species at 3 sites (Appendix S4). Nevertheless, particular sites differed in frequency of occurrence and/or abundance of particular species, and values of DAI and DAS association indices differed between investigated sites. Therefore, the core of the fauna differed strongly. For example, *Maniola jurtina*, one of the most common and abundant species, had similar values of DAS index (15%–23%) for all the sites, but DAI values showed that Traktorowa hosted the highest number of individuals (DAI = 49%), followed by Rogi site (DAI = 35%) (Appendix S4).

#### 3.1.1 Brukowa Site

Altogether 36 species were recorded at the Brukowa site, but only 3 with frequency of occurrence higher than 40%: *Coenonympha pamphilus* (*F* = 62.2% 1.6 ± 2.6 ind./500 m), *Pieris napi* (*F* = 62.2% 2 ± 2.6 ind./500 m) and *Pieris rapae* (*F* = 62.2% 3.6 ± 3.9 ind./500 m). The highest DAI and DAS were obtained for *Lasiomata megera* (DAI = 32.6%, DAS = 50%), *Colias hyale* (DAI = 31.3%, DAS = 37.5%) and *Nymphalis antiopa* (DAI = 40.5%, DAS = 33.3%).

#### 3.1.2 Maratońska Site

Altogether, 41 species were recorded here, and all 5 families were represented. It was the site with the highest number of species. Eleven species had a frequency of occurrence higher than 40% on this site. Most of them were also very abundant. The highest values were recorded for: *Coenonympha pamphilus* (*F* = 88.2% 10.8 ± 10.9 ind./500 m), *Pieris rapae* (*F* = 71.1% 3.4 ± 3.6 ind./500 m), *Lycaena phleas* (*F* = 62.2% 1.6 ± 2 ind./500 m) and *Lycaena tityrus* (*F* = 62.2% 2.6 ± 3.4 ind./500 m). The highest DAI and DAS were recorded for *Polyommatus coridon* (DAI = 100%, DAS = 100%), *Melitaea cinxia* (DAI = 100%, DAS = 100%), and *Pontia edusa* (DAI = 89.1%, DAS = 80.8%).

#### 3.1.3 Rogi Site

Altogether, 36 species were found at the Rogi Site, including 6 species with frequency of occurrence higher than 40%. The most frequent and abundant species were *Pieris napi* (*F* = 64.2% 2.5 ± 3.1 ind./500 m), *Coenonympha pamphilus* (*F* = 59.5% 3.2 ± 3.9 ind./500 m) and *Polyommatus icarus* (*F* = 52.4% 1.9 ± 2.7 ind./500 m). The highest DAI and DAS were recorded for *Satyrium w*‐*album* (DAI = 100%, DAS = 100%) and *Argynnis paphia* (DAI = 90.7%, DAS = 76.9%).

#### 3.1.4 Telefoniczna Site

Altogether, 36 species were recorded here, but only 4 had a frequency higher than 40%. *Coenonympha pamphilus* (*F* = 72.1% 3.6 ± 4.1 ind./500 m) and *Pieris rapae* (*F* = 76.7% 2.7 ± 3.7 ind./500 m) were the most frequent. The highest DAI and DAS were recorded for *Satyrium pruni* (DAI = 100%, DAS = 100%), *Polygonia c‐album* (DAI = 36.7%, DAS = 40.5%), and *Lycaena dispar* (DAI = 41.7%, DAS = 36.8%).

#### 3.1.5 Traktorowa Site

With 34 species recorded, it was the least speciose site and the only one lacking *Papilio machaon* , and therefore family Papilionidae. Seven species had a frequency higher than 40%. The most frequent species were *Coenonympha pamphilus* (*F* = 70.7% 4.3 ± 5.6 ind./500 m), *Pieris napi* (*F* = 63.4% 3.4 ± 3.6 ind./500 m) and *Lycaena phleas* (*F* = 63.4% 1.2 ± 1.2 ind./500 m). The highest DAI and DAS were recorded for *Brenthis ino* (DAI = 100%, DAS = 100%), *Carcharodus alceae* (DAI = 100%, DAS = 100%), and *Araschnia levana* (DAI = 77%, DAS = 60%).

### 3.2 Diversity and Abundance at Investigated Sites

**FIGURE 3:**
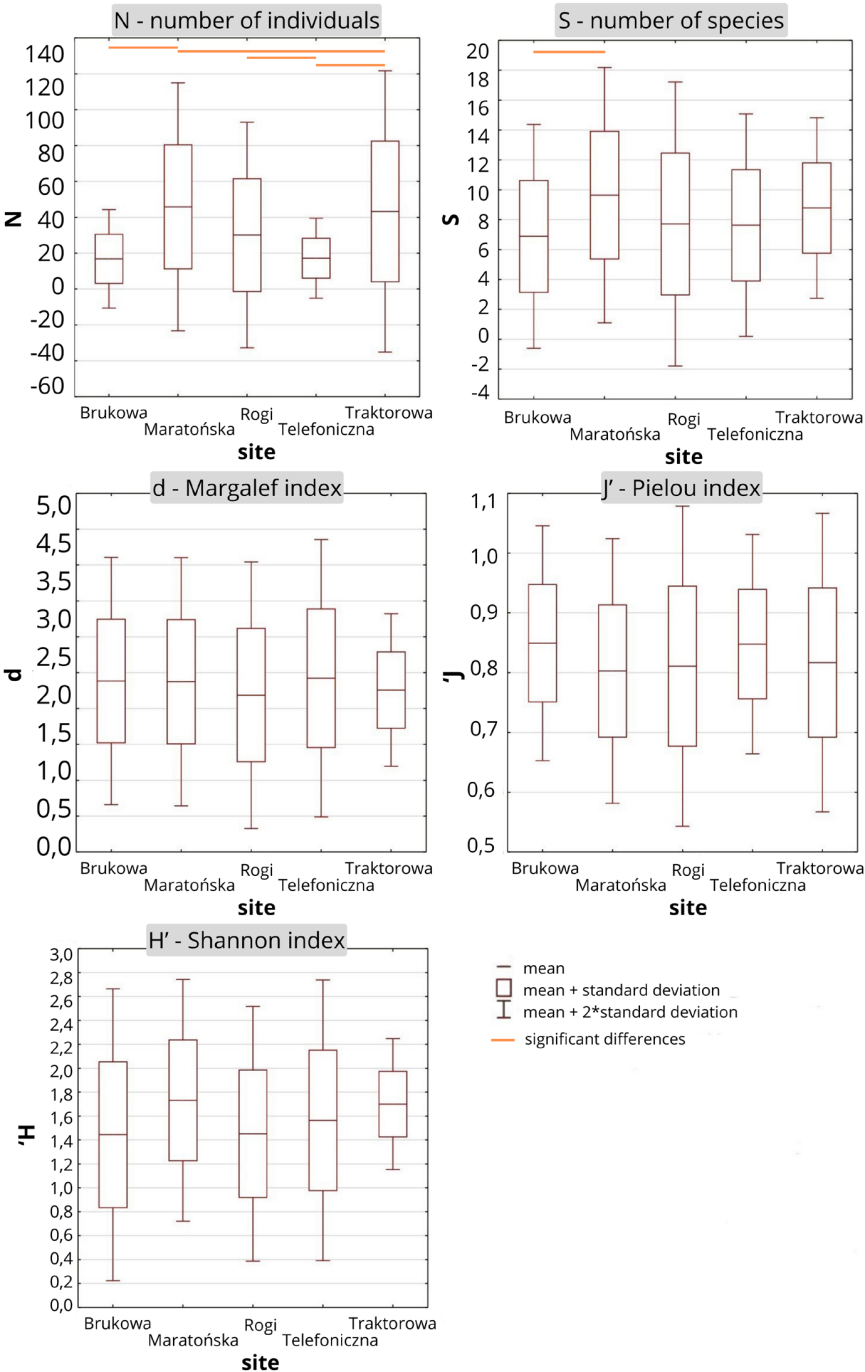
Mean values (with standard deviation) of abundance and diversity indices for each site. Statistically significant pairs are marked using strait lines (Kruskal–Wallis test, Dunn test *p* < 0.05).

The highest mean abundance of butterflies was noted in Maratońska (46 ± 35 ind./500 m) and Traktorowa (43 ± 39 ind./500 m). The lowest abundance was recorded in Brukowa (17 ± 14 ind./500 m) (Figure 3). Mean species richness per sample was similar on all the sites (Figure 3). The highest value was recorded in Maratońska (10 ± 4), and the lowest in Brukowa (7 ± 4). The highest mean value of the Margalef index was recorded in Telefoniczna (2.4 ± 1) and the lowest in Rogi (2.2 ± 1).

**FIGURE 4:**
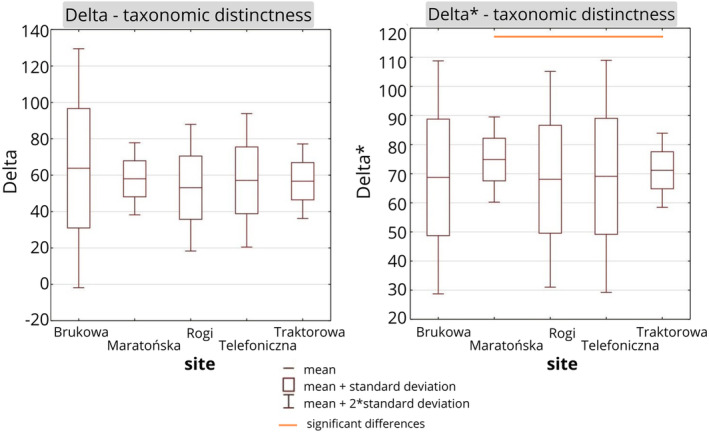
Mean values (with standard deviation) of taxonomic diversity Delta (Δ) and taxonomic distinctness Delta⁎ (Δ⁎). Statistically significant pairs are marked using strait lines (Kruskal–Wallis test, Dunn test *p* < 0.05).

Values of diversity indices and evenness were overall similar on all the sites (Figure 3). The mean value of the Shannon index was the highest in Maratońska (1.7 ± 0.5) and the lowest in Brukowa (1.4 ± 0.6). Evenness was the highest in Brukowa (0.9 ± 0.1) and the lowest in Maratońska (0.8 ± 0.1). The taxonomic distinctness Delta was the highest in Brukowa (62.8 ± 32.8) and the lowest in Rogi (53.1 ± 17.4), although there were no statistically significant differences (Kruskal–Wallis test *p* < 0.05). Taxonomic distinctness Delta* was the highest in Maratońska (74.9 ± 7.3) and the lowest in Rogi (68.1 ± 18.5) (Figure 4).

No seasonal effects (2019 vs. 2020) were detected except for Brukowa for abundance (Mann–Whitney *U* test, *p* < 0.05), species richness (*t*‐test, *p* < 0.05), evenness (*t*‐test, *p* < 0.05), and diversity (Shannon index) (Mann–Whitney *U* test, p < 0.05) Significantly lower values of Delta* were recorded only in Telefoniczna in 2020 (Mann–Whitney *U* test, *p* < 0.05).

### 3.3 Similarity Analysis

**FIGURE 5:**
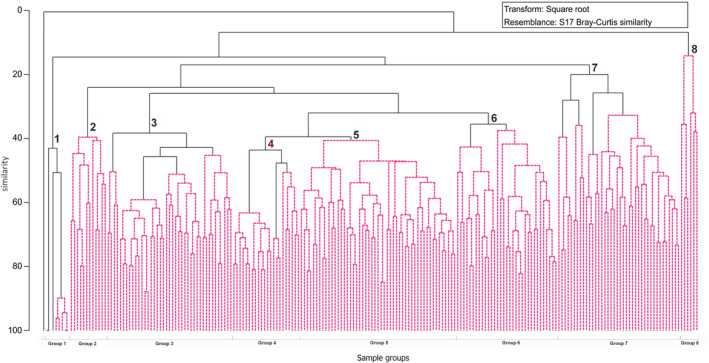
Dendrogram of samples (Bray–Curtis formula group−average method square root transformed data). Spotted lines indicate groups that were differentiated by SIMPROF test with 5% significance level.

Similarity analysis allowed the description of seasonal and spatial patterns in species distribution. Seasonal differences were most pronounced with differences between the species‐poor early spring and end of summer, (Clusters 1, 2, and 7) and the most speciose summer months (Clusters 3, 4, 5). Maratońska and Traktorowa were most clearly separated from the other sites. Cluster 6 (samples from May and June) was also spatially divided between Brukowa and Rogi. A dendrogram based on Bray‐Curtis's similarity index showed eight clusters mostly on a 40%–50% similarity level (Figure 5, Appendix S14). SIMPROOF differentiated 18 smaller groups, but we have decided to follow a more general pattern because of the faunistic composition of sub‐clusters was very similar. Species composition and characteristics of each group are included in Appendices S5 and S6.

SIMPER analysis pointed at *Coenonympha pamphilus*, *Pieris rapae* , *Pieris napi* , *Maniola jurtina*, *Meleanargia galathea*, and *Aphantopus hypernatus* as the species with the highest contributions for similarity within clusters as well as dissimilarities with other clusters, that is, *Coenonympha pamphilus* contributed over 25% to the similarity in cluster 5 and over 50% in cluster 6. Except for *Polyommatus coridon*, less numerous species like *Aglais io*, *Gonepteryx rhamni*, *Polyommatus icarus*, and *Lycaena tityrus* also contributed to the grouping results, for example, *A. io* was associated with cluster 7 (similarity contribution 26.9%) and *P. coridon* with cluster 4 (similarity contribution 13.9%). Details of SIMPER results are provided in Appendix S7.

### 3.4 Fauna Changes Throughout the Whole Season

Most of the species recorded in Łódź were bivoltine, but it was not always clearly visible due to temporal overlap of generations. For most of the sites, the abundance peak was recorded in July (Weeks 15–17) except at Maratońska, where the peak was visible around Week 20 and was associated with an increased abundance of *P. coridon* and the second generation of *C. pamphilus* (Appendices [Link], [Link], [Link], [Link], [Link], [Link]). In general, peaks of abundance were recorded when the highest number of species co‐occur and during seasonal maximums of the main dominants. For example, in Traktorowa in 2020, the peak of species number was recorded in Week 14, while the peak of abundance was recorded a week later as a result of a higher abundance of *Aphantopus hyperantus*.

Seasonal changes in species composition resulted in divisions into three main periods: (1) start of the season/early spring, (2) middle season/late spring and summer, (3) end of the season/late summer, and early autumn (Appendices [Link], [Link], [Link], [Link], [Link], [Link]). The beginning of the season revealed the presence of species overwintering as adult forms, mainly *Aglais io*, *Polygonia c‐album*, or *Gonepteryx rhamni*, accompanied by early spring species like *Anthocharis cardamines*, *Pieris napi* and *Pieris rapae* . Middle season starts at the turn of May and June, with the appearance of *Ochlodes sylvanus* and *Coenonympha pamphilus*. This latter species is active for most of the season, together with *Pieris rapae* and *Pieris napi* . Middle season is characterised by the highest number of co‐occurring species. *Maniola jurtina* and *Melanarghia galanthea* contributed to the highest total abundance of butterflies on sites where they dominate, like Traktorowa and Rogi. End of season is marked by the general decline of the abundance of individuals representing first or second generation (e.g., *Melanarghia galanthea*, *Maniola jurtina*) and the emergence of the third generation of species like *Pieris napi* , *Pieris rapae* , *Polyommaus icarus*, *Lycaena phlaeas* , and *Lycaena tityrus*. In general, the 2019 season was more humid and warmer than the 2020 season. It was especially visible in the colder early spring of 2020. However, abundance was higher in 2020 with the exception of Brukowa (Appendix S8), which suffered some additional disturbances discussed later in the paper. Increasing temperatures corresponded with the beginning of activity for some species like *A. hyperantus* at all sites, *T. lineola* at Maratońska and Telefoniczna, *T. silvestris* at Traktorowa, and *M. jurtina* at Traktorowa.

## 4 Discussion

### 4.1 Local‐Regional Species Pools: Urban Fauna versus Semi‐Natural and Agricultural Areas of Central Poland

Only 72 out of 160 Polish butterfly species were recorded recently in Central Poland as a result of large, long‐term citizen science projects (Buszko and Masłowski 2008; Buszko and Nowacki 2017) as this region is dominated mostly by agricultural habitats or planted pine forests (Kurowski 2013).

Therefore, the local species pools of particular urban habitat patches strongly reflect the regional species pool of Central Poland. Nevertheless, it remains difficult to interpret why species like *Pyrgus malvae*, *Hesperia comma* , *Hyponephele lycaon Callophrys rubi*, *Boloria selene* , or *Melitaea athalia* do not penetrate urban habitats despite the fact that their host plants are common in Łódź (Witosławski 2006) and their nearest populations are found in the Łagiewniki Forest, which is located within the borders of the city and well connected with the urbanised areas (including sites like Brukowa and Rogi) by a net of ecological corridors, which includes large parks and/or railway tracks (Sobczyk, Pabis, and Wieczorek 2018). Similar observations were recently presented for Gdynia for the same group of species (Senn 2015). *H. comma* and *P. malvae* are generally associated with dry meadows or ruderal sites (Sielezniew and Dziekańska 2010), and their absence in the large wastelands in Łódź is very surprising, pointing at the necessity of more detailed studies of their biology and factors that could restrict their distribution in the city, but also on a larger scale as urbanisation is progressing throughout Central Europe (Restrepo Cadavid et al. 2017). Additionally, *H. comma* is a relatively good disperser that may move between habitat patches as a result of stepping‐stone colonisation (Davies et al. 2005).

Species like *Cupido argiades* and *Lycaena dispar* are currently common in Łódź. At the end of XX century, *C. argiades* was quite rare in Central Poland, and it was declining throughout the whole country (Buszko and Masłowski 2008). *C. argiades* later expansion in Poland and high abundance in Łódź might be associated with climate warming as this has previously been demonstrated in other parts of Europe (Warren et al. 2021) and due to the presence of the heat island effect typical for urbanised areas (Santamouris 2007; McCarthy and Sanderson 2011). *Lycaena dispar* is included in the EU Habitat Directive and was recently included in the landscape‐scale restoration actions in some European countries (Warren et al. 2021). It was initially associated with wetlands, but its habitat requirements are changing, probably as a result of climate warming (Sielezniew and Dziekańska 2010; Lindman et al. 2015), accompanied by changes in host plant preferences (more xerophilous species of *Rumex*) (Martin and Pullin 2004; Buszko and Masłowski 2008). It was observed in Łódź on dry cultivated lawns, flying between blocks of flats, and at the same time is absent from many theoretically more suitable locations outside the city (personal observations), which makes its urban population a very interesting model for more comprehensive studies of development and/or evolutionary differences that may include not only changes in host plant utilisation but also thermal preferences or susceptibility to parasitoids between urban and non‐urban populations (Martin and Pullin 2004; Angilletta Jr et al. 2007, Diamond and Martin 2021; Theodorou 2022) or influence of toxic metals like copper and lead that accumulate in its host plants (Cakaj et al. 2023). We can even speculate that urban areas might constitute a refuge from a typically disturbed agricultural landscape.

Single habitat patches strongly reflected the species composition of the surrounding region, which confirms earlier observations that landscape‐level drivers of species diversity are important for butterflies (Viljur et al. 2020). It is even more pronounced when we look at urbanisation zones in Łódź. For example, Brukowa and Telefoniczna—relatively small sites located in the second urbanisation zone of Łódź hosted 36 species out of 38 species recorded in this zone (Sobczyk, Pabis, and Wieczorek 2018). Theoretically, over a large timescale, almost all species from a regional species pool would reach almost every community (Hillebrand and Blenckner 2002), but will not necessarily stay there for longer periods of time. Moreover, typically, the influence of the regional species pool on local fauna is the strongest when local communities are less structured by species interactions (mostly competition) and when many species are rare (Cornell and Harrison 2014). In the case of Łódź, the urban habitats were invaded by almost all available taxa from the regional species pool, which mostly came from a small number of common taxa. It is also worth mentioning that number of such watelands in Lodz is generally low and chosen sites constitute good representation of such habitats in this city. We did not noticed differences in species richness between the sites, than can be clearly attributed to distance from the city center or distance from the borders of the city. Maratonska was located close to the city border and had the highest richness, although the less diverse site on Rogi have the same character and it is neighbouring to Lagiewniki Forest and non‐urbanised areas. Since the caterpillar host plants of the recorded species are generally common in Lodz (Witosławski 2006), including investigated sites, we can assume that those species reproduce in the city.

### 4.2 Small‐Scale Diversity—Insight Into Details of Community Composition and Diversity

The urban wastelands of Łódź are characterised by an interesting and complex blend of various butterfly species co‐occurring in small, isolated, and very restricted sites. There is no quantitative data about butterfly communities in agriculture habitats in Central Poland, but particular habitat patches (e.g., meadows, areas around crop fields, or pine forest edges) have much lower butterfly species richness (personal observations) than urban habitat patches recorded in Łódź, where each small isolated site hosted from 34 to 41 species of butterflies. Even some relict and natural habitats have lower diversity than this, although with a higher number of rare or specialised taxa. For example, clearings isolated by large forests and covered by molinia meadows located in the Bolimowski Landscape Park, about 60 km from Łódź. There, we can find species like *P. nausithous*, *P. teius*, and *P. alcon* but not *P. aegeria*, *L. aliciphron*, *L. coridon*, *T. betulae* , or *L. megera* (Kurowski 2013, personal observations). Studies from Swedish grasslands showed that the number of species per site most often varied between 5 and 20, moreover, investigated sites were not homogenous and differed in species composition (Bergman et al. 2018). Similar results were also recorded for particular sites sampled in Northern Italy (Guariento et al. 2022). Butterfly communities associated with wastelands in Łódź include common species characterised in agricultural areas like pierids, grassland species like satyrines and hesperides, forest‐dwelling taxa like *P. aegeria* and *A. ilia*, species associated with shrubs like hairstreaks, large nymphalidae associated with *Urtica dioica* , thermophilus *P. machaon* which is typical for open spaces, but also some more specialised species, like *L. dispar* or *L. coridon* (Bartonova, Benes, and Konvicka 2014; Buszko and Masłowski 2008). Majority of species recorded in Lodz can be considered good dispersers (Bartonova, Benes, and Konvicka 2014), associated with common species of plants (Buszko and Masłowski 2008). Moreover, *L. coridon* is absent in Central Poland, which was confirmed by results of the long term citizen science project (Buszko 1997; Buszko and Masłowski 2008; personal observations) despite the availability of suitable habitats, therefore the population in Łódź is highly isolated but probably benefits from the heat islands effect or consists of a relict population. A similar ecological blend in community composition was already observed on urban habitat patches in Birmingham (Angold et al. 2006). Urban habitats might become an important refuge for some species of pollinators, although not always for Lepidoptera (Ockinger et al. 2009; Hall et al. 2017; Twerd, Sobieraj‐Betlińska, and Szefer 2021; Dylewski, Maćkowiak, and Banaszak‐Cibicka 2019; Theodorou 2022). A majority of earlier studies pointed at urbanisation as one of the main causes of diversity loss in pollinators (Aguilera et al. 2019; Theodorou 2022; Herrmann et al. 2023). However, we lack data about butterfly communities from urban wastelands, which are generally neglected in ecological studies and management or conservation strategies. At the same time, many studies are focused on the role of urban parks in maintaining urban diversity (e.g., Konvicka and Kadlec 2011; Sing et al. 2016; Han et al. 2022, Jasmani, Lamit, and van den Bosch 2022). Earlier results from Łódź demonstrated that even the largest parks host only a dozen or so butterfly species (Sobczyk, Pabis, and Wieczorek 2018). Similar differences between parks and ruderal sites were observed in Poznan (Dylewski, Maćkowiak, and Banaszak‐Cibicka 2019) and in Malmo (Ockinger et al. 2009), including lower species loss over time (Aguilera et al. 2019).

Our conclusions are confirmed by values of the Margalef Index and Shannon index as well as by values of taxonomic distinctness—indices that showed relatively high phylogenetic diversity. At the same time, evenness values and detailed analysis of the dominance structure (Appendix S4) point to a lack of communities highly dominated by one or two of the most eurytopic or resistant species, for instance in the case of rural butterfly communities numerically dominated by *P. napi* *s* and *P. rapae* (Feber et al. 1997; De Snoo et al. 1998; Sikkink, Kobiela, and Snell‐Rood 2017). The high abundance of those species in agricultural landscapes results from the strong dominance of their host plants and monoculture plant communities (Outhwaite, McCann, and Newbold 2022; Tan et al. 2022).

On the other hand, the high butterfly diversity of urban wastelands might be explained by high small‐scale plant diversity, which is not typical for species‐poor and highly disturbed agricultural landscapes surrounding the city (Jaskulski and Jaskulska 2012; Ma and Herzon 2014; Guerra et al. 2022). Chemisation and cultivation practises that lead to a simplification of plant communities in the agricultural areas surrounding Łódź are probably pushing butterflies into urban refuges. Accidental and random transport of various plant species into the city, disturbance at initial stages of succession, and the highly mosaic character of different microhabitats and soil types created by various human activities, lead to a blend of plant species characterised by different ecological requirements at one site (Maurer, Peschel, and Schmitz 2000; Czortek and Pielech 2020; Czortek et al. 2020; Czortek 2023), which was also observed in Łódź in general (Witosławski 2006) and at all sites investigated during this study (Appendix S1). This creates a mix of various resources, including host and nectar plants, open spaces, and elevated temperatures, which are beneficial for many butterflies (Wallis de Vries and van Swaay 2009; Cormont et al. 2011; Gordon and Kerr 2022). For example, dry open, warmer habitats select for increased fecundity and even longevity of species like *C. pamphilus*, *P. aegeria*, and *A. hyperantus* (Karlsson and Wiklund 2004), which are common in Łódź. Elevated temperatures typical for urban areas also increase the dispersal abilities of species like *M. jurtina* and *C. pamphilus*, enabling them to cope with fragmented landscapes (Cormont et al. 2011). Moreover, Łódź is dominated by grassland butterflies, indeed recent studies demonstrated that the surrounding forest is beneficial for many of them (Bergman et al. 2018).

Isolated urban habitat islands are becoming true microcosms, although unstable and prone to dynamic change. The urban green space that was a diversity hot spot in one season, may become deserted over the course of a year or less. Changes might be permanent (e.g., creation of new buildings or parking lots) or temporary and less pronounced (e.g., management practise, moving, creation of a new park, or changes along some important ecological corridor leading to a given site) and may also result from a modification in vegetation succession stages during each year (Strauss and Biedermann 2006; Aguilera et al. 2019; Habel et al. 2019). It was visible in the shifts that occurred at Brukowa in 2020 when species like B. *dia*, *L*. *alciphron*, *L. dispar* , and *A. cardamines* disappeared as a result of renovations along the railroad tracks. Railway‐associated habitats, like the Brukowa Site, are pointed out as potentially favourable for butterflies (Kalarus and Bąkowski 2015), especially in the cities like Łódź‐ densely urbanised and not crossed by any large rivers. Additionally, railways may serve as ecological corridors (Moroń et al. 2014; Moroń et al. 2017), therefore, we might expect a relatively fast recovery of the communities. Inspections in the following years (2021 and 2022) revealed that plant cover at the Brukowa Site is recovering, however, it has not reached its former abundance thus far.

At first glance, all 5 sites are similar and represent typical urban wastelands inhabited by the same group of about 25 butterflies and are characterised by similar values for diversity indices regardless of the distance from the city centre or city borders. Therefore, migration distance within the city seems to be less important for butterfly distribution, as was previously observed in Malmo (Ockinger et al. 2009). On the other hand, sites located in the most urbanised part of the city (Brukowa and Telefoniczna) had the lowest abundance and species numbers, which is probably related to these sites having the highest rate of disturbance (Blair and Launer 1997; Blair 1999; Matsumoto 2015). Despite general similarities, the butterfly communities of particular sites differ in abundance and frequency of occurrence for particular species (Appendix S4). Even theoretically the most common central European species like *I. lathonia*, *T. lineola* , *P. aegeria*, *A. hyperantus*, or *A. io* are not stable and abundant in communities at all sites. Those differences might result from the details of habitat characteristics, but we can speculate that they might also be driven by extinctions and migrations between habitat islands, as explained by MacArthur and Wilson's island biogeography theory, which was already applied to urban green spaces (Laurance 2008; Medeiros‐Sousa et al. 2017; Dunn et al. 2022) and migration–immigration dynamics in general (Hambäck and Englund 2005). Those processes may be responsible for differences in the frequency of occurrence and/or abundance of particular species between 2019 and 2020, like in the case of *L. phlaeas* , *T. betulae* , or *M. galathea* at Brukowa.

Analysis of phenological dynamics during both seasons did not demonstrate any major differences between the investigated sites nor between the seasons, pointing to a stable phenological pattern.. The spring species *A. cardamines* occurred earlier on Telefoniczna in 2019, although it is difficult to interpret this difference. Heat island effect seems to be visible in Łódź only in early spring, but even *A. cardamines*, *P. napi* and *P. rapae* occured in typical time for Poland (Buszko and Masłowski 2008). None of the species developed additional generations when compared to the typical phenology in Poland, although *I. lathonia* developed three generations in Łódź. It is not typical for all parts of Poland, but does not stand out from phenology in the central part of the country (Sielezniew and Dziekańska 2010; Buszko and Masłowski 2008). Similar observation concern *L. dispar* which is bivoltine in Łódź, but not always in natural habitats (Buszko and Masłowski 2008). Our results may suggest some changes in species' temporal overlap, which might be caused by increased temperatures (Gezon et al. 2018). This indicates possible changes in competition for resources (Gezon et al. 2018) or dys‐synchronisation with host plant development (Donoso et al. 2016; Schenk et al. 2018), although these aspects need further, more comprehensive studies.

## 5 Conclusions and Future Studies

This study revealed interesting research questions that should be addressed in the near future to enhance our understanding of urban butterfly community dynamics. There is a great need for mark‐release‐recapture studies, studies of genetic diversity, and connectivity of local urban populations, including migration within the city and from surrounding areas (Takami et al. 2004). There is undoubtedly a group of species that can be considered as best suited for city life, including *C. pamphilus*, *M. jurtina*, *P. napi* , *P. rapae* , and *P. icarus* . These species are often recorded in urban habitats of the central European cities (Machnikowski 1999; Höttinger 2000; Winiarska 2003; Ockinger et al. 2009; Konvicka and Kadlec 2011; Gelbrecht et al. 2016; Palik et al. 2005; Senn 2015; Dennis et al. 2017; Dylewski, Maćkowiak, and Banaszak‐Cibicka 2019; Sielezniew and Dziekańska 2019), and it is worthwhile to conduct more comprehensive studies of their ecology in cities.

Our results demonstrated high butterfly diversity in the urban wastelands, which might be important not only from a well‐being perspective and education of citizens (Taylor and Hochuli 2015) but also for providing protection to butterflies in Central Europe, where urban wastelands appear to be small‐scale faunal refugia of higher importance than a local scale within city borders. Since European butterfly populations are declining (Warren et al. 2021), urban habitats may constitute refuges for some species, especially grassland butterflies, but also taxa like *L. dispar* . Nevertheless, the role of wastelands will be diminished if green spaces are destroyed as a result of urban management. Therefore, it is crucial to link the results of ecological studies with appropriate urban planning that is suitable for a particular area (Niemelä 1999) and well‐planned educational actions, facilitating city citizens' involvement. For example, our studies of the butterfly fauna of Łódź resulted in a publication of the open‐access Field Guide to Butterflies in Łódź. This book was planned as a background for future citizen science actions related to the protection of green spaces in the city. Moreover, our results could be valuable for city management and can act as a reference point for further research in the whole region.

## Author Contributions

**Sylwia Pietrzak:** conceptualization (equal), formal analysis (lead), investigation (lead), methodology (equal), writing – original draft (equal), writing – review and editing (equal). **Krzysztof Pabis:** conceptualization (equal), formal analysis (supporting), investigation (supporting), methodology (equal), supervision (lead), writing – original draft (equal), writing – review and editing (equal).

## Conflicts of Interest

The authors declare no conflicts of interest.

## Supporting information


**Appendix S1.** List of plant species (excluding Poaceae) recorded on sampling sites. (M—Maratońska, B—Brukowa, R—Rogi, TL—Telefoniczna, TR—Traktorowa).

**Appendix S2.** Description of sampling sites.

**Appendix S3.** Classification of species to higher taxonomic units that was used for calculation of taxonomic distinctness. Nomenclature based on Kristensen et al. (2007) and Buszko and Masłowski (2008). Species in alphabetical order.

**Appendix S4.** Species composition, frequency of occurrence (F), abundance (M—mean, SD—standard deviation, MAX—maximum value per single transect) and values of association indices (DAI and DAS) at each site. The most important species on each site are marked in bold.

**Appendix S5.** Species composition, frequency of occurrence (F), abundance (M—mean, SD—standard deviation, MAX—maximum value per single transect) and values of association indices (DAI and DAS) for each cluster derived from Bray–Curtis similarity dendrogram (clusters 1–4).

**Appendix S6.** Species composition, frequency of occurrence (F), abundance (M—mean, SD—standard deviation, MAX—maximum value per single transect) and values of association indices (DAI and DAS) for each cluster derived from Bray–Curtis similarity dendrogram (clusters 5–8).

**Appendix S7.** SIMPER report.

**Appendix S8.** Seasonal changes of butterfly communities along 2019 and 2020 sampling seasons on Brukowa site on a background of weather conditions (humidity, temperature, cloudiness, wind speed). Abundance values per transect are given in the table.

**Appendix S9.** Seasonal changes of butterfly communities along 2019 and 2020 sampling seasons on Maratońska site on a background of weather conditions (humidity, temperature, cloudiness, wind speed). Abundance values per transect are given in the table.

**Appendix S10.** Seasonal changes of butterfly communities along 2019 and 2020 sampling seasons on Rogi site on a background of weather conditions (humidity, temperature, cloudiness, wind speed). Abundance values per transect are given in the table.

**Appendix S11.** Seasonal changes of butterfly communities along 2019 and 2020 sampling seasons on Telefoniczna site on a background of weather conditions (humidity, temperature, cloudiness, wind speed). Abundance values per transect are given in the table.

**Appendix S12.** Seasonal changes of butterfly communities along 2019 and 2020 sampling seasons on Traktorowa site on a background of weather conditions (humidity, temperature, cloudiness, wind speed). Abundance values per transect are given in the table.

**Appendix S13.** Species counts for all analysed sites. Samples labels were codded with combination of information about the year, observation week, site abbreviation and sample number (i.e., 2019_02_B_01). B—Brukowa, R—Rogi, M—Maratonska, TL—Telefoniczna, TR—Traktorowa.

**Appendix S14.** Samples from each dendrogram group.

## Data Availability

All raw data used in this study are available in Appendix S13 of this article.
