# Supplementary material for: Life on Green Patches: Diversity and Seasonal Changes of Butterfly Communities Associated With Wastelands of the Post‐Industrial Central European City
Source: Ecol Evol. 2024 Dec 16;14(12):e70695. doi: 10.1002/ece3.70695 (PMC11650753; doi:10.1002/ece3.70695)
Supplement: Supplementary file 1 — Appendix S1. List of plant species (excluding Poaceae) recorded on sampling sites. (M—Maratońska, B—Brukowa, R—Rogi, TL—Telefoniczna, TR—Traktorowa). [file ECE3-14-e70695-s007.docx]

Appendix1. List of plant species (excluding Poaceae) recorded on sampling sites. (M – Maratońska, B – Brukowa, R – Rogi, TL- Telefoniczna, TR – Traktorowa).

|  |  |  | **Presence on sites** | | | | |
| --- | --- | --- | --- | --- | --- | --- | --- |
| **Name of the species** | **Family** | **Genus** | M | B | R | TL | TR |
| *Sambucus nigra ssp. nigra* | Adoxaceae | *Sambucus* |  |  |  | X |  |
| *Allium vineale* | Amaryllidaceae | *Allium* | X | X | X |  |  |
| *Aegopodium podagraria* | Apiaceae | *Aegopodium* |  |  |  |  | X |
| *Anthriscus sylvestris* | Apiaceae | *Anthriscus* |  |  | X |  |  |
| *Chaerophyllum temulum* | Apiaceae | *Chaerophyllum* |  |  | X |  |  |
| *Daucus carota ssp. carota* | Apiaceae | *Daucus* | X | X | X | X | X |
| *Heracleum sphondylium ssp. glabrum* | Apiaceae | *Heracleum* |  |  |  |  | X |
| *Heracleum sphondylium ssp. sphondylium/glabrum* | Apiaceae | *Heracleum* |  | X |  |  |  |
| *Pastinaca sativa* | Apiaceae | *Pastinaca* |  | X | X |  |  |
| *Peucedanum oreoselinum* | Apiaceae | *Peucedanum* |  | X |  | X |  |
| *Pimpinella saxifraga ssp. saxifraga* | Apiaceae | *Pimpinella* |  | X | X |  |  |
| *Pimpinella sp.* | Apiaceae | *Pimpinella* |  |  | X |  |  |
| *Torilis japonica* | Apiaceae | *Torilis* |  | X | X | X | X |
| *Achillea millefolium* | Asteraceae | *Achillea* |  | X | X |  | X |
| *Achillea vulgaris* | Asteraceae | *Achillea* |  |  |  | X |  |
| *Alchemilla millefolium* | Asteraceae | *Alchemilla* |  |  |  | X |  |
| *Anchusa officinalis* | Asteraceae | *Anchusa* | X |  |  |  |  |
| *Aquilegia ×hybrida/vulgaris* | Asteraceae | *Aquilegia* |  |  | X |  |  |
| *Arctium tomentosum* | Asteraceae | *Arctium* |  |  |  |  |  |
| *Artemisia absinthium* | Asteraceae | *Artemisia* | X |  |  |  |  |
| *Artemisia camperstris ssp. campestris* | Asteraceae | *Artemisia* | X | X | X | X | X |
| *Artemisia vulgaris* | Asteraceae | *Artemisia* | X | X | X | X |  |
| *Cantaurea stoebe* | Asteraceae | *Cantaurea* | X | X | X | X | X |
| *Carduus acanthoides* | Asteraceae | *Carduus* |  | X | X |  |  |
| *Centaurea jacea* | Asteraceae | *Centaurea* |  |  | X |  |  |
| *Cerastium sp.* | Asteraceae | *Cerastium* |  |  | X |  | X |
| *Chamomilla suaveolens* | Asteraceae | *Chamomilla* |  |  |  | X |  |
| *Cichorium intybus ssp. intybus* | Asteraceae | *Cichorium* |  | X | X | X | X |
| *Cirsium arvense* | Asteraceae | *Cirsium* |  | X | X | X | X |
| *Cirsium vulgare* | Asteraceae | *Cirsium* |  | X | X | X | X |
| *Conyza canadensis* | Asteraceae | *Conyza* | X | X |  | X | X |
| *Coreopsis lanceolata* | Asteraceae | *Coreopsis* |  |  |  |  | X |
| *Echium vulgare* | Asteraceae | *Echium* | X | X | X |  |  |
| *Erigeron acris* | Asteraceae | *Erigeron* |  |  | X |  |  |
| *Erigeron annuus ssp. annuus* | Asteraceae | *Erigeron* |  | X | X | X | X |
| *Erigeron annuus ssp. septentrionalis* | Asteraceae | *Erigeron* | X | X | X |  |  |
| *Galinsoga parviflora* | Asteraceae | *Galinsoga* |  | X |  |  |  |
| *Helianthus sp.* | Asteraceae | *Helianthus* |  | X |  | X |  |
| *Helichrysum arenarium* | Asteraceae | *Helichrysum* | X |  | X |  | X |
| *Heliopsis scabra* | Asteraceae | *Heliopsis* |  |  |  |  | X |
| *Hieracium pilosella* | Asteraceae | *Hieracium* |  | X | X | X | X |
| *Hieracium sabaudum* | Asteraceae | *Hieracium* |  | X |  |  | X |
| *Hieracium umbellatum var. umbellatum* | Asteraceae | *Hieracium* |  |  | X | X | X |
| *Hypericum perforatum* | Asteraceae | *Hypericum* | X | X | X | X | X |
| *Hypochoeris radicata* | Asteraceae | *Hypochoeris* | X | X | X | X | X |
| *Jasione montana* | Asteraceae | *Jasione* | X | X | X |  | X |
| *Knautia arvensis* | Asteraceae | *Knautia* |  | X |  | X |  |
| *Lactuca serriola* | Asteraceae | *Lactuca* |  | X |  |  | X |
| *Lapsana communis* | Asteraceae | *Lapsana* |  | X | X |  |  |
| *Leontodon autumnalis ssp. autumnalis* | Asteraceae | *Leontodon* | X | X |  | X | X |
| *Matricaria perforata* | Asteraceae | *Matricaria* |  |  |  | X |  |
| *Rudbeckia hirta* | Asteraceae | *Rudbeckia* |  |  | X |  | X |
| *Rudbeckia hirta var. hirta* | Asteraceae | *Rudbeckia* |  |  |  |  |  |
| *Senecio jacobaea* | Asteraceae | *Senecio* | X | X |  |  | X |
| *Senecio vulgaris* | Asteraceae | *Senecio* |  | X |  |  |  |
| *Solidago ×niederederi* | Asteraceae | *Solidago* |  |  |  |  | X |
| *Solidago canadensis* | Asteraceae | *Solidago* |  | X | X | X | X |
| *Solidago gigantea* | Asteraceae | *Solidago* |  | X |  | X |  |
| *Solidago virgaurea* | Asteraceae | *Solidago* |  | X | X | X | X |
| *Sonchus asper* | Asteraceae | *Sonchus* |  | X |  |  |  |
| *Tanacetum vulgare* | Asteraceae | *Tanacetum* |  | X | X | X | X |
| *Taraxacum officinale coll.* | Asteraceae | *Taraxacum* |  |  | X | X |  |
| *Tragopogon dubius* | Asteraceae | *Tragopogon* | X | X |  |  |  |
| *Tragopogon sp.* | Asteraceae | *Tragopogon* |  | X | X |  |  |
| *Impatiens glandulifera* | Balsaminaceae | *Impatiens* |  |  |  |  | X |
| *Myosotis arvensis* | Boraginaceae | *Myosotis* |  | X |  |  |  |
| *Alliaria petiolata* | Brassicaceae | *Alliaria* |  |  | X |  |  |
| *Arabidopsis thaliana* | Brassicaceae | *Arabidopsis* |  | X |  |  |  |
| *Barbarea vulgaris* | Brassicaceae | *Barbarea* |  | X |  |  |  |
| *Berteroa incana* | Brassicaceae | *Berteroa* | X | X | X | X | X |
| *Cardaminopsis arenosa ssp. arenosa* | Brassicaceae | *Cardaminopsis* |  |  |  |  |  |
| *Descurainia sophia* | Brassicaceae | *Descurainia* |  |  |  | X |  |
| *Lepidium campestre* | Brassicaceae | *Lepidium* |  | X |  |  |  |
| *Lunaria annua* | Brassicaceae | *Lunaria* |  | X |  |  |  |
| *Raphanus raphanistrum* | Brassicaceae | *Raphanus* |  | X |  |  |  |
| *Rorippa sp.* | Brassicaceae | *Rorippa* |  | X |  |  |  |
| *Sisymbrium loeselii* | Brassicaceae | *Sisymbrium* |  | X | X | X | X |
| *Campanula rapunculoides* | Campanulaceae | *Campanula* |  |  | X | X |  |
| *Dianthus deltoides* | Caryophyllacaeae | *Dianthus* |  |  |  | X |  |
| *Melandrium album* | Caryophyllacaeae | *Melandrium* | X | X | X | X | X |
| *Saponaria officinalis* | Caryophyllacaeae | *Saponaria* | X | X | X |  |  |
| *Saponaria officinalis f. plena* | Caryophyllacaeae | *Saponaria* |  |  | X |  |  |
| *Silene vulgaris* | Caryophyllacaeae | *Silene* |  | X | X |  |  |
| *Stellaria graminea* | Caryophyllacaeae | *Stellaria* |  | X |  | X |  |
| *Convolvulus arvensis* | Convolvulaceae | *Convolvulus* | X | X | X | X | X |
| *Sedum maximum* | Crassulaceae | *Sedum* |  |  | X | X |  |
| *Echinocystsis lobata* | Cucurbitaceae | *Echinocystsis* |  |  |  |  | X |
| *Euphorbia esula* | Euphorbiaceae | *Euphorbia* |  | X | X | X |  |
| *Euphorbia helioscopia* | Euphorbiaceae | *Euphorbia* |  | X |  |  |  |
| *Caragana arborescens* | Fabaceae | *Caragana* |  |  |  |  |  |
| *Coronilla varia* | Fabaceae | *Coronilla* | X | X | X |  |  |
| *Cytisus scoparius* | Fabaceae | *Cytisus* |  |  | X |  |  |
| *Lathyrus latifolius* | Fabaceae | *Lathyrus* |  |  | X |  | X |
| *Lathyrus tuberosus* | Fabaceae | *Lathyrus* |  |  | X |  |  |
| *Lotus corniculatus* | Fabaceae | *Lotus* |  | X | X | X |  |
| *Lupinus polyphyllus* | Fabaceae | *Lupinus* |  |  |  | X |  |
| *Medicago ×varia* | Fabaceae | *Medicago* |  | X | X |  |  |
| *Medicago falcata* | Fabaceae | *Medicago* |  | X |  |  |  |
| *Medicago lupulina* | Fabaceae | *Medicago* |  |  | X |  |  |
| *Medicago sativa* | Fabaceae | *Medicago* |  |  |  | X |  |
| *Melilotus alba* | Fabaceae | *Melilotus* |  | X |  | X |  |
| *Melilotus officinalis* | Fabaceae | *Melilotus* |  | X | X | X |  |
| *Robinia pseudoacacia* | Fabaceae | *Robinia* | X | X | X | X |  |
| *Trifolium arvense* | Fabaceae | *Trifolium* | X | X | X | X | X |
| *Trifolium campestre* | Fabaceae | *Trifolium* |  |  | X |  |  |
| *Trifolium medium* | Fabaceae | *Trifolium* |  | X | X | X |  |
| *Trifolium pratense ssp. pratense* | Fabaceae | *Trifolium* |  |  | X | X |  |
| *Trifolium pratense ssp. sativum* | Fabaceae | *Trifolium* |  | X |  |  |  |
| *Trifolium repens ssp. repens* | Fabaceae | *Trifolium* |  |  | X | X |  |
| *Vicia cracca* | Fabaceae | *Vicia* |  | X | X | X |  |
| *Vicia villosa* | Fabaceae | *Vicia* |  | X |  |  | X |
| *Geranium molle* | Geraniaceae | *Geranium molle* |  |  |  |  | X |
| *Geranium robertianum* | Geraniaceae | *Geranium* |  | X |  |  |  |
| *Ballota nigra ssp. nigra* | Lamiaceae | *Ballota* |  |  | X | X |  |
| *Betonica officinalis* | Lamiaceae | *Betonica* |  |  |  | X |  |
| *Lamium purpureum* | Lamiaceae | *Lamium* |  | X |  |  |  |
| *Leonurus cardiaca* | Lamiaceae | *Leonurus* |  |  | X |  |  |
| *Mentha ×villosa* | Lamiaceae | *Mentha* |  |  | X |  |  |
| *Origanum vulgare* | Lamiaceae | *Origanum* |  | X |  |  | X |
| *Lythrum saliciaria* | Lythraceae | *Lythrum* |  |  |  |  | X |
| *Malus sp.* | Malvaceae | *Malus* |  |  | X | X |  |
| *Lavatera thuringiaca* | Malvaceae | *Lavatera* |  |  | X |  |  |
| *Ligustrum vulgare* | Oleaceae | *Ligustrum* |  |  |  | X |  |
| *Epilobium hirsutum* | Onagraceae | *Epilobium* |  |  |  |  | X |
| *Epilobium lamyi* | Onagraceae | *Epilobium* |  | X |  |  |  |
| *Epilobium montanum* | Onagraceae | *Epilobium* |  | X |  |  |  |
| *Oenothera sp./spp.* | Onagraceae | *Oenothera* | X | X | X | X | X |
| *Chelidonium majus* | Papaveraceae | *Chelidonium* |  | X | X |  | X |
| *Papaver dubium* | Papaveraceae | *Papaver* |  | X |  | X |  |
| *Papaver rhoeas* | Papaveraceae | *Papaver* |  |  | X |  |  |
| *Linaria vulgaris* | Plantaginaceae | *Linaria* |  | X |  |  | X |
| *Plantago lanceolata* | Plantaginaceae | *Plantago* |  |  | X | X | X |
| *Reynoutria japonica* | Polygonaceae | *Reynoutria* |  |  |  |  |  |
| *Polygonum rurivagum* | Polygonaceae | *Polygonum* |  | X |  |  |  |
| *Reseda lutea* | Resedaceae | *Reseda* |  | X |  |  |  |
| *Crataegus sp.* | Rosaceae | *Crataegus* |  |  | X | X |  |
| *Filipendula ulmaria* | Rosaceae | *Filipendula* |  |  |  |  | X |
| *Geum urbanum* | Rosaceae | *Geum* |  | X | X | X |  |
| *Padus serotina* | Rosaceae | *Padus* |  | X | X | X | X |
| *Potentilla anserina* | Rosaceae | *Potentilla* |  |  |  |  | X |
| *Potentilla argentea* | Rosaceae | *Potentilla* |  | X | X |  | X |
| *Potentilla dissecta/impolita* | Rosaceae | *Potentilla* |  | X |  |  | X |
| *Potentilla intermedia* | Rosaceae | *Potentilla* |  |  | X |  | X |
| *Potentilla repens* | Rosaceae | *Potentilla* |  |  | X | X | X |
| *Potentilla tenuiloba* | Rosaceae | *Potentilla* |  |  | X | X |  |
| *Prunus cerasifera* | Rosaceae | *Prunus* |  | X | X |  |  |
| *Prunus sp.* | Rosaceae | *Prunus* |  |  |  | X |  |
| *Rosa sp.* | Rosaceae | *Rosa* | X | X | X | X |  |
| *Rubus caesius* | Rosaceae | *Rubus* | X | X |  | X |  |
| *Rubus idaeus* | Rosaceae | *Rubus* |  |  |  | X |  |
| *Rubus sp.* | Rosaceae | *Rubus* |  |  |  |  | X |
| *Sanguisorba minor* | Rosaceae | *Sanguisorba* |  | X |  |  |  |
| *Sorbus aucuparia* | Rosaceae | *Sorbus* |  |  |  | X |  |
| *Galium album* | Rubiaceae | *Galium* |  | X |  |  |  |
| *Galium verum* | Rubiaceae | *Galium* |  |  |  | X |  |
| *Acer campestris* | Sapindaceae | *Acer* |  |  | X |  |  |
| *Acer platanoides* | Sapindaceae | *Acer* |  |  | X |  |  |
| *Verbascum densiflorum* | Scrophulariaceae | *Verbascum* | X | X |  | X |  |
| *Verbascum nigrum* | Scrophulariaceae | *Verbascum* | X |  |  |  |  |
| *Verbascum phlomoides* | Scrophulariaceae | *Verbascum* |  |  | X |  |  |
| *Verbascum sp.* | Scrophulariaceae | *Verbascum* |  |  |  |  |  |
| *Solanum dulcamara* | Solanaceae | *Solanum* |  | X |  |  |  |
| *Veronica chamaedrys* | Veronicaceae | *Veronica* |  |  | X |  | X |
| *Viola arvensis* | Violaceae | *Viola* |  | X |  |  |  |
| *Viola tricolor* | Violaceae | *Viola* |  | X |  |  |  |
| **Total number of species** |  |  | 30 | 91 | 84 | 68 | 56 |
