# Supplementary material for: Life on Green Patches: Diversity and Seasonal Changes of Butterfly Communities Associated With Wastelands of the Post‐Industrial Central European City
Source: Ecol Evol. 2024 Dec 16;14(12):e70695. doi: 10.1002/ece3.70695 (PMC11650753; doi:10.1002/ece3.70695)
Supplement: Supplementary file 2 — Appendix S2. Description of sampling sites. [file ECE3-14-e70695-s011.docx]

Appendix 2. Description of sampling sites.

| **Brukowa (B)**  It is adjacent to working field of company involved in recycling, including metals, electronical scrap, industrial and constructional wastes. Transect followed through ruderal shrubs and side of railroad tracks that are leading to the northern borders of the city. Vegetation included shrubs, small woodland, meadow and railroad-associated flora. Distinction between part following path from Brukowa street to railroad and part along the railroad was clearly visible. Except from early spring part of the site that is not attached to railroad was mostly covered from direct sunlight by leaves of trees and shrubs, thus larger spots of more exposed vegetation can be treated as separate microhabitat. Flora along this path was composed mostly of various grassess mixed with shrubs of *Robinia pseudoacatia*, *Prunus* sp and *Rubus* sp.*.* Flowering herbaceous plants were i.e. *Berteroa incana, Cirsium* sp.*, Erigeron* sp*, Knautia arvensis, Lotus corniculatus,* *Melilotus albus,* *Tanacetum vulgare*, *Trifolium* sp*.*. In the summer this part was dominated by *Solidago* sp.  Part along the railway was initially (spring time) covered with *Cardaminopsis arenosa, Viola* sp and *Geranium* sp., later species like *Berteroa incana, Erigeron* sp*, Oenothera* sp*, Origanium vulgare, Echium vulgare, Hypericum* sp*, Reseda lutea, Medicago* sp*.*, *Linaria vulgaris* dominated*. Solidago* sp. was also present here, but not as densely like in the other part of the site. |
| --- |
| **Maratońska (M)**  The route of the transect led between forest edge and linear mound running along the road (Sanitariuszek Street) then curved following forest edge, through open space between the forest and Maratońska Street. Comprehensively it can be described as a mix of dry meadow with a shrub patches restricted by roads. Vegetation included dry meadow and ecotone zone between meadow and coniferous forest. Shrubs (*Rubus, Robinia pseudacacia*, and *Syringa vulgaris*) were intersecting meadow into smaller compartments, creating heterogenous mosaic of habitats, that included patchess of lower and higher grasses or exposed sandy ground. Flora of the site was dominated by Poaceae e.g. *Corynephorus canescens.* Flowering plants include: *Berteroa incana, Centaurea stoebe*, *Erigeron* sp., *Prunus* sp*., Hieracium pilosella, Jasione montana, Viola* sp*, Echium vulgare,* *Knautia arvensis,* *Securigera varia, Anchusa officinalis,* and *Solidago* sp., |
| **Rogi (R)**  Transect led next to residential area, neighbouring to elemental school facility and ongoing construction of new block of flats. There was also a hill of anthropogenic origin on this site. The hill was covered with mixed patches of forest, meadow, shrubs and orchard. Transect followed a path through slope, hilltop and open habitats of meadowy character below the hill. Vegetation included meadows characterized by different level of moisture and ecotone zone between them and deciduous forest. Microhabitats include sand pits. The site use to be mowed until 2019. This change resulted in development of higher vegetation especially in more humid part of the site. Flora consists of grasses of different moisture preference, shrubs (*Sambucus nigra*, low *Malus* sp and young *Robinia pseudoacatia*). Flowering herbaceous plants include: *Cirsium* sp, *Pastinaca sativa, Daucus carota, Trifolium* sp.*, Vicia* sp*, Lotus corniculatus, Hieracium* sp*., Potentilla* sp*., Jasione montana, Knautia arvensis* and some plants associated with neighbouring estate gardens like *Rudbeckia hirta* and *Lathyrus latifolius*. |
| **Telefoniczna (TL)**  Transect led through hilly fragmentary humid unused area covered with a mix of ruderal vegetation, grassland, shrubs and forest. Woodland patches can be described as mixed forest although deciduous trees (e.g. birch) dominate. Grassland is partially enclosed by trees and shrubs. In the 2019 vegetation clearance was done under powerlines that are crossing the site resulting in increased connectivity between microhabitat patches. The most charismatic flowering herbaceous plants here are i.e: *Berteroa incana*, *Erigeron* sp*.*, *Melilotus albus, Melilotus officinalis, Lupinus polyphyllus,* *Convolvulus arvensis, Hieracium pilosella*, *Knautia arvensis*, *Stellaria* sp*.*, *Lotus corniculatus, Vicia* sp, *Trifolium arvense* andvarious Apiaceae i.e. *Daucus carota.* During flowering period *Solidago* sp. largely dominated most of this site. |
| **Traktorowa (TR)**  Transect followed through patches of humid meadow, ruderal meadow, forest clearing and 100 m part that have forestry character. Final section was located next to horse stables area and crop field.  More humid part of the meadow, located closer to the river was covered by *Cardaminopsis arenosa* and *Veronica chamaedrys* at the beginning of the season. Later it was dominated by various flowering plants i.e. *Ranunculaceae*, *Berteroa* *incana*, *Achillea vulgaris*, *Silene flos-cuculi, Cirsium* sp*.*, *Potentilla* sp*., Linaria vulgaris* and *Tanacetum vulgare*. Three patches of dryer meadow differ in flowering plant composition. First patch was adjacent to the humid area and flora include: *Helichrysum arenarium, Jasione montana, Vicia* sp*., Hieracium pilosella*, *Achillea vulgaris*, *Berteroa incana, Knautia arvensis, Senecio* sp*.*, *Centaurea stoebe*, *Potentilla* sp*.*, *Tanacetum vulgare* and *Solidago gigantea*. Grasses, were not the main part of vegetation here*.* Second patch was dominated by grasses and flowering plants like: *Hieracium* sp., *Vicia* sp., *Knautia arvensis*, *Jasione montana,* *Senecio* sp. Third patch was adjacent to forest. Flowering plants include *Rubus* sp*.*, *Jasione montana*, *Knautia arvensis* and *Hieracium* sp. Later during the season *Solidago virgaurea* and *Solidago gigantea* covered this part of the site. There are also some Apiaceae, *Urtica* sp.*, Lamium* sp. and dense coverage of *Impatiens glandulifera* along the edge of the forest. |
