# Supplementary material for: Life on Green Patches: Diversity and Seasonal Changes of Butterfly Communities Associated With Wastelands of the Post‐Industrial Central European City
Source: Ecol Evol. 2024 Dec 16;14(12):e70695. doi: 10.1002/ece3.70695 (PMC11650753; doi:10.1002/ece3.70695)
Supplement: Supplementary file 3 — Appendix S3. Classification of species to higher taxonomic units that was used for calculation of taxonomic distinctness. Nomenclature based on Kristensen et al. (2007) and Buszko and Masłowski (2008). Species in alphabetical order. [file ECE3-14-e70695-s014.docx]

Appendix 3. Classification of species to higher taxonomic units that was used for calculation of taxonomic distinctness. Nomenclature based on Kristensen et al. (2007) and Buszko and Masłowski (2017). Species in alphabetical order.

| **Species** | **Superfamily** | **Familiy** | **Subfamily** | **Tribe** | **Genus** |
| --- | --- | --- | --- | --- | --- |
| Aglais_io | Papilionoidea | Nymphalidae | Nymphalinae | Nymphalini | Aglais |
| Aglais_urticae | Papilionoidea | Nymphalidae | Nymphalinae | Nymphalini | Aglais |
| Anthocharis_cardamines | Papilionoidea | Pieridae | Pierinae | Anthocharini | Anthocharis |
| Apatura_ilia | Papilionoidea | Nymphalidae | Apaturinae | Apaturini | Apatura |
| Aphantopus_hyperantus | Papilionoidea | Nymphalidae | Satyrinae | Maniolini | Aphantopus |
| Araschnia_levana | Papilionoidea | Nymphalidae | Nymphalinae | Nymphalini | Araschnia |
| Argynnis_paphia | Papilionoidea | Nymphalidae | Heliconiinae | Argynnini | Argynnis |
| Aricia_agestis | Papilionoidea | Lycaenidae | Polyommatinae | Polyommatini | Aricia |
| Boloria_dia | Papilionoidea | Nymphalidae | Heliconiinae | Argynnini | Boloria |
| Brenthis_ino | Papilionoidea | Nymphalidae | Heliconiinae | Argynnini | Brenthis |
| Carcharodus_alceae | Hesperioidea | Hesperiidae | Pyrginae | - | Carcharodus |
| Celastrina_argiolus | Papilionoidea | Lycaenidae | Polyommatinae | Polyommatini | Celastrina |
| Coenonympha_glycerion | Papilionoidea | Nymphalidae | Satyrinae | Coenonymphini | Coenonympha |
| Coenonympha_pamphilus | Papilionoidea | Nymphalidae | Satyrinae | Coenonymphini | Coenonympha |
| Colias_hyale | Papilionoidea | Pieridae | Coliadinae | Coliadini | Colias |
| Cupido_argiades | Papilionoidea | Lycaenidae | Polyommatinae | Polyommatini | Cupido |
| Erynnis_tages | Hesperioidea | Hesperiidae | Pyrginae | - | Erynnis |
| Gonepteryx_rhamni | Papilionoidea | Pieridae | Coliadinae | Gonepterygini | Gonepteryx |
| Issoria_lathonia | Papilionoidea | Nymphalidae | Heliconiinae | Argynnini | Issoria |
| Lasiommata_megera | Papilionoidea | Nymphalidae | Satyrinae | Elymniini | Lasiommata |
| Leptidea_juvernica | Papilionoidea | Pieridae | Dismorphiinae | Leptideini | Leptidea |
| Lycaena_alciphron | Papilionoidea | Lycaenidae | Lycaeninae | Lycaenini | Lycaena |
| Lycaena_dispar | Papilionoidea | Lycaenidae | Lycaeninae | Lycaenini | Lycaena |
| Lycaena_phlaeas | Papilionoidea | Lycaenidae | Lycaeninae | Lycaenini | Lycaena |
| Lycaena_tityrus | Papilionoidea | Lycaenidae | Lycaeninae | Lycaenini | Lycaena |
| Maniola_jurtina | Papilionoidea | Nymphalidae | Satyrinae | Maniolini | Maniola |
| Melanargia_galathea | Papilionoidea | Nymphalidae | Satyrinae | Melanargiini | Melanargia |
| Melitaea_cinxia | Papilionoidea | Nymphalidae | Melitaeinae | Melitaeini | Melitaea |
| Nymphalis_antiopa | Papilionoidea | Nymphalidae | Nymphalinae | Nymphalini | Nymphalis |
| Ochlodes_sylvanus | Hesperioidea | Hesperiidae | Hesperiinae | - | Ochlodes |
| Papilio_machaon | Papilionoidea | Papilionidae | Papilioninae | Papilionini | Papilio |
| Pararge_aegeria | Papilionoidea | Nymphalidae | Satyrinae | Elymniini | Pararge |
| Pieris_brassicae | Papilionoidea | Pieridae | Pierinae | Pierini | Pieris |
| Pieris_napi | Papilionoidea | Pieridae | Pierinae | Pierini | Pieris |
| Pieris_rapae | Papilionoidea | Pieridae | Pierinae | Pierini | Pieris |
| Polygonia_c-album | Papilionoidea | Nymphalidae | Nymphalinae | Nymphalini | Polygonia |
| Polyommatus_coridon | Papilionoidea | Lycaenidae | Polyommatinae | Polyommatini | Polyommatus |
| Polyommatus_icarus | Papilionoidea | Lycaenidae | Polyommatinae | Polyommatini | Polyommatus |
| Pontia_edusa | Papilionoidea | Pieridae | Pierinae | Pierini | Pontia |
| Satyrium_pruni | Papilionoidea | Lycaenidae | Theclinae | Eumaeini | Satyrium |
| Satyrium_w-album | Papilionoidea | Lycaenidae | Theclinae | Eumaeini | Satyrium |
| Thecla_betulae | Papilionoidea | Lycaenidae | Theclinae | Theclini | Thecla |
| Thymelicus_lineola | Hesperioidea | Hesperiidae | Hesperiinae | - | Thymelicus |
| Thymelicus_sylvestris | Hesperioidea | Hesperiidae | Hesperiinae | - | Thymelicus |
| Vanessa_atalanta | Papilionoidea | Nymphalidae | Nymphalinae | Nymphalini | Vanessa |
| Vanessa_cardui | Papilionoidea | Nymphalidae | Nymphalinae | Nymphalini | Vanessa |
| Aglais_io | Papilionoidea | Nymphalidae | Nymphalinae | Nymphalini | Aglais |
