# Supplementary material for: Life on Green Patches: Diversity and Seasonal Changes of Butterfly Communities Associated With Wastelands of the Post‐Industrial Central European City
Source: Ecol Evol. 2024 Dec 16;14(12):e70695. doi: 10.1002/ece3.70695 (PMC11650753; doi:10.1002/ece3.70695)
Supplement: Supplementary file 4 — Appendix S4. Species composition, frequency of occurrence (F), abundance (M—mean, SD—standard deviation, MAX—maximum value per single transect) and values of association indices (DAI and DAS) at each site. The most important species on each site are marked in bold. [file ECE3-14-e70695-s004.docx]

Appendix 4. Species composition, frequency of occurrence (F), abundance (M – mean, SD – standard deviation, MAX – maximum value per single transect) and values of association indices (DAI and DAS) at each site. The most important species on each site are marked in bold.

|  | **Brukowa** | | | | | | **Maratońska** | | | | | | **Rogi** | | | | | | **Telefoniczna** | | | | | | **Traktorowa** | | | | | | **Total** | |
| --- | --- | --- | --- | --- | --- | --- | --- | --- | --- | --- | --- | --- | --- | --- | --- | --- | --- | --- | --- | --- | --- | --- | --- | --- | --- | --- | --- | --- | --- | --- | --- | --- |
| **species** | F | M | SD | MAX | DAS | DAI | F | M | SD | MAX | DAS | DAI | F | M | SD | MAX | DAS | DAI | F | M | SD | MAX | DAS | DAI | F | M | SD | MAX | DAS | DAI | F | Ind |
| *Carcharodus alceae* | 0,0% | - | - | - | 0,0% | 0,0% | - | - | - | - | - | - | - | - | - | - | - | - | - | - | - | - | - | - | 2,4% | 0,02 | 0,16 | 1,00 | 100,0% | 100,0% | 0,5% | 1 |
| *Erynnis tages* | 11,6% | 0,08 | 0,23 | 0,71 | 20,8% | 10,0% | 17,8% | 0,38 | 1,25 | 7,71 | 33,3% | 48,0% | 21,4% | 0,32 | 0,71 | 2,86 | 37,5% | 38,0% | 4,7% | 0,03 | 0,15 | 0,71 | 8,3% | 4,0% | - | - | - | - | - | - | 11,2% | 46 |
| *Ochlodes sylvanus* | 14,0% | 0,28 | 0,86 | 4,29 | 16,7% | 12,6% | 8,9% | 0,21 | 0,82 | 4,29 | 11,1% | 9,8% | 19,0% | 0,58 | 1,66 | 7,14 | 22,2% | 25,1% | 25,6% | 0,71 | 1,72 | 7,14 | 30,6% | 31,8% | 17,1% | 0,49 | 1,47 | 7,00 | 19,4% | 20,7% | 16,8% | 125 |
| *Thymelicus lineola* | 7,0% | 0,07 | 0,26 | 1,43 | 8,3% | 3,8% | **28,9%** | **0,90** | **2,29** | **12,00** | **36,1%** | **52,9%** | 7,1% | 0,07 | 0,26 | 1,43 | 8,3% | 3,8% | 23,3% | 0,28 | 0,59 | 2,14 | 27,8% | 15,9% | 17,1% | 0,44 | 1,12 | 5,00 | 19,4% | 23,6% | 16,8% | 90 |
| *Thymelicus sylvestris* | 11,6% | 0,10 | 0,29 | 1,43 | 17,9% | 8,2% | 8,9% | 0,11 | 0,39 | 1,71 | 14,3% | 9,8% | 11,9% | 0,17 | 0,52 | 2,14 | 17,9% | 13,6% | 9,3% | 0,12 | 0,41 | 2,14 | 14,3% | 9,5% | 24,4% | 0,76 | 1,71 | 8,00 | 35,7% | 59,0% | 13,1% | 60 |
| *Aricia agestis* | 23,2% | 0,32 | 0,65 | 2,14 | 15,6% | 10,4% | **53,3%** | **1,52** | **1,98** | **7,71** | **37,5%** | **52,6%** | 19,0% | 0,15 | 0,34 | 1,43 | 12,5% | 4,9% | 16,3% | 0,13 | 0,32 | 1,43 | 10,9% | 4,4% | 36,6% | 0,88 | 1,95 | 11,00 | 23,4% | 27,6% | 29,9% | 152 |
| *Celastrina argiolus* | 7,0% | 0,07 | 0,26 | 1,43 | 23,1% | 20,0% | 11,1% | 0,15 | 0,49 | 2,57 | 38,5% | 48,0% | 4,8% | 0,03 | 0,15 | 0,71 | 15,4% | 10,0% | 4,7% | 0,05 | 0,24 | 1,43 | 15,4% | 15,0% | 2,4% | 0,02 | 0,16 | 1,00 | 7,7% | 7,0% | 6,1% | 18 |
| *Cupido argiades* | 27,9% | 0,50 | 1,14 | 5,71 | 28,6% | 34,6% | 6,7% | 0,06 | 0,22 | 0,86 | 7,1% | 4,2% | **40,5%** | **0,54** | **0,80** | **2,86** | **40,5%** | **37,0%** | 23,3% | 0,35 | 0,79 | 3,57 | 23,8% | 24,2% | - | - | - | - | - | - | 19,6% | 86 |
| *Lycaena alciphron* | 7,0% | 0,08 | 0,36 | 2,14 | 21,4% | 22,5% | 15,6% | 0,19 | 0,51 | 2,57 | 50,0% | 54,1% | - | - | - | - | - | - | 2,3% | 0,02 | 0,11 | 0,71 | 7,1% | 4,5% | 7,3% | 0,07 | 0,26 | 1,00 | 21,4% | 18,9% | 6,5% | 19 |
| *Lycaena dispar* | 11,6% | 0,10 | 0,29 | 1,43 | 26,3% | 25,0% | 2,2% | 0,02 | 0,13 | 0,86 | 5,3% | 5,0% | 9,5% | 0,07 | 0,21 | 0,71 | 21,1% | 16,7% | 16,3% | 0,17 | 0,49 | 2,86 | 36,8% | 41,7% | 4,9% | 0,05 | 0,22 | 1,00 | 10,5% | 11,7% | 8,9% | 23 |
| *Lycaena phlaeas* | 30,2% | 0,27 | 0,44 | 1,43 | 15,1% | 7,5% | **62,2%** | **1,66** | **2,00** | **8,57** | **32,6%** | **48,9%** | 14,3% | 0,12 | 0,31 | 1,43 | 7,0% | 3,3% | 30,2% | 0,27 | 0,47 | 2,14 | 15,1% | 7,5% | **63,4%** | **1,22** | **1,26** | **5,00** | **30,2%** | **32,8%** | 40,2% | 176 |
| *Lycaena tityrus* | 18,6% | 0,20 | 0,45 | 1,43 | 11,1% | 4,0% | **62,2%** | **2,65** | **3,41** | **12,86** | **38,9%** | **55,3%** | 9,5% | 0,10 | 0,34 | 1,43 | 5,6% | 2,0% | 23,3% | 0,27 | 0,58 | 2,86 | 13,9% | 5,3% | **53,7%** | **1,76** | **2,37** | **10,00** | **30,6%** | **33,4%** | 33,6% | 245 |
| *Polyommatus coridon* | 0,0% | - | - | - | 0,0% | 0,0% | **46,7%** | **9,24** | **15,17** | **55,71** | **100,0%** | **100,0%** | - | - | - | - | - | - | - | - | - | - | - | - | - | - | - | - | - | - | 9,8% | 485 |
| *Polyommatus icarus* | **39,5%** | **0,95** | **1,91** | **7,14** | **17,0%** | **15,6%** | **53,3%** | **1,49** | **2,01** | **7,71** | **24,0%** | **25,5%** | **52,4%** | **1,89** | **2,71** | **10,00** | **22,0%** | **30,3%** | **48,8%** | **1,11** | **1,75** | **8,57** | **21,0%** | **18,3%** | 39,0% | 0,66 | 1,04 | 4,00 | 16,0% | 10,3% | 46,7% | 340 |
| *Satyrium pruni* | 0,0% | - | - | - | 0,0% | 0,0% | - | - | - | - | - | - | - | - | - | - | - | - | 2,3% | 0,02 | 0,11 | 0,71 | 100,0% | 100,0% | - | - | - | - | - | - | 0,5% | 1 |
| *Satyrium w-album* | 0,0% | - | - | - | 0,0% | 0,0% | - | - | - | - | - | - | 2,4% | 0,02 | 0,11 | 0,71 | 100,0% | 100,0% | - | - | - | - | - | - | - | - | - | - | - | - | 0,5% | 1 |
| *Thecla betulae* | 0,0% | - | - | - | 0,0% | 0,0% | 2,2% | 0,02 | 0,13 | 0,86 | 7,7% | 5,6% | 7,1% | 0,05 | 0,19 | 0,71 | 23,1% | 14,0% | 14,0% | 0,10 | 0,25 | 0,71 | 46,2% | 28,0% | 7,3% | 0,20 | 0,95 | 6,00 | 23,1% | 52,3% | 6,1% | 18 |
| *Aglais io* | 30,2% | 0,47 | 0,92 | 4,29 | 22,4% | 18,6% | 24,4% | 0,67 | 1,81 | 9,43 | 19,0% | 27,9% | 16,7% | 0,19 | 0,47 | 2,14 | 12,1% | 7,3% | 32,6% | 0,55 | 1,23 | 5,71 | 24,1% | 21,9% | 31,7% | 0,63 | 1,41 | 8,00 | 22,4% | 24,2% | 27,1% | 133 |
| *Aglais urticae* | 0,0% | - | - | - | 0,0% | 0,0% | 2,2% | 0,02 | 0,13 | 0,86 | 33,3% | 33,3% | - | - | - | - | - | - | 2,3% | 0,02 | 0,11 | 0,71 | 33,3% | 27,8% | 2,4% | 0,02 | 0,16 | 1,00 | 33,3% | 38,9% | 1,4% | 3 |
| *Apatura ilia* | 2,3% | 0,02 | 0,11 | 0,71 | 12,5% | 10,0% | - | - | - | - | - | - | 9,5% | 0,10 | 0,37 | 2,14 | 50,0% | 60,0% | 7,0% | 0,05 | 0,18 | 0,71 | 37,5% | 30,0% | - | - | - | - | - | - | 3,7% | 10 |
| *Aphantopus hyperantus* | 23,3% | 0,96 | 2,37 | 10,71 | 19,6% | 10,4% | 17,8% | 0,69 | 1,82 | 8,57 | 15,7% | 7,8% | 26,2% | 1,99 | 4,51 | 17,86 | 21,6% | 21,1% | 18,6% | 0,70 | 1,95 | 10,00 | 15,7% | 7,6% | 34,1% | 5,15 | 10,11 | 39,00 | 27,5% | 53,2% | 23,8% | 464 |
| *Araschnia levana* | 4,7% | 0,03 | 0,15 | 0,71 | 5,0% | 2,0% | 6,7% | 0,06 | 0,22 | 0,86 | 7,5% | 3,7% | 16,7% | 0,19 | 0,47 | 2,14 | 17,5% | 11,2% | 9,3% | 0,10 | 0,37 | 2,14 | 10,0% | 6,1% | **58,5%** | **1,32** | **1,78** | **8,00** | **60,0%** | **77,0%** | 18,7% | 76 |
| *Argynnis paphia* | 0,0% | - | - | - | 0,0% | 0,0% | 2,2% | 0,02 | 0,13 | 0,86 | 7,7% | 2,8% | 23,8% | 0,66 | 2,03 | 12,14 | 76,9% | 90,7% | - | - | - | - | - | - | 4,9% | 0,05 | 0,22 | 1,00 | 15,4% | 6,5% | 6,1% | 42 |
| *Boloria dia* | 9,3% | 0,20 | 0,99 | 6,43 | 25,0% | 26,3% | 17,8% | 0,40 | 1,03 | 4,29 | 50,0% | 55,3% | - | - | - | - | - | - | - | - | - | - | - | - | 9,8% | 0,15 | 0,48 | 2,00 | 25,0% | 18,4% | 7,5% | 39 |
| *Brenthis ino* | 0,0% | - | - | - | 0,0% | 0,0% | - | - | - | - | - | - | - | - | - | - | - | - | - | - | - | - | - | - | 4,9% | 0,05 | 0,22 | 1,00 | 100,0% | 100,0% | 0,9% | 2 |
| *Coenonympha glycerion* | 0,0% | - | - | - | 0,0% | 0,0% | 2,2% | 0,02 | 0,13 | 0,86 | 50,0% | 54,5% | 2,4% | 0,02 | 0,11 | 0,71 | 50,0% | 45,5% | - | - | - | - | - | - | - | - | - | - | - | - | 0,9% | 2 |
| *Coenonympha pamphilus* | **51,2%** | **1,63** | **2,63** | **9,29** | **15,3%** | **6,8%** | **82,2%** | **10,84** | **10,92** | **46,29** | **25,7%** | **47,7%** | **59,5%** | **3,18** | **3,92** | **12,86** | **17,4%** | **13,1%** | **72,1%** | **3,62** | **4,14** | **14,29** | **21,5%** | **15,2%** | **70,7%** | **4,27** | **5,57** | **25,00** | **20,1%** | **17,1%** | 67,3% | 1247 |
| *Issoria lathonia* | 14,0% | 0,13 | 0,39 | 2,14 | 12,5% | 6,7% | **53,3%** | **1,39** | **1,70** | **5,14** | **50,0%** | **73,2%** | 16,7% | 0,17 | 0,44 | 2,14 | 14,6% | 8,4% | 14,0% | 0,12 | 0,31 | 1,43 | 12,5% | 5,9% | 12,2% | 0,12 | 0,33 | 1,00 | 10,4% | 5,9% | 22,4% | 103 |
| *Lasiommata megera* | 6,9% | 0,05 | 0,18 | 0,71 | 50,0% | 32,6% | 4,4% | 0,08 | 0,40 | 2,57 | 33,3% | 52,2% | - | - | - | - | - | - | - | - | - | - | - | - | 2,4% | 0,02 | 0,16 | 1,00 | 16,7% | 15,2% | 2,8% | 8 |
| *Maniola jurtina* | 32,6% | 0,96 | 1,73 | 5,71 | 14,7% | 3,4% | **48,9%** | **1,89** | **3,04** | **11,14** | **23,2%** | **6,9%** | **47,6%** | **10,12** | **16,54** | **60,71** | **21,1%** | **34,7%** | 39,5% | 1,74 | 3,14 | 11,43 | 17,9% | 6,1% | **53,7%** | **14,59** | **23,80** | **86,00** | **23,2%** | **48,8%** | 44,4% | 1455 |
| *Melanargia galathea* | 25,6% | 0,63 | 1,57 | 8,57 | 17,5% | 7,6% | 33,3% | 2,57 | 5,45 | 24,86 | 23,8% | 32,4% | 33,3% | 2,57 | 6,03 | 27,14 | 22,2% | 30,2% | 27,9% | 0,93 | 2,01 | 7,86 | 19,0% | 11,2% | 26,8% | 1,61 | 3,44 | 15,00 | 17,5% | 18,5% | 29,4% | 446 |
| *Melitaea cinxia* | 0,0% | - | - | - | 0,0% | 0,0% | 4,4% | 0,08 | 0,36 | 1,71 | 100,0% | 100,0% | - | - | - | - | - | - | - | - | - | - | - | - | - | - | - | - | - | - | 0,9% | 4 |
| *Nymphalis antiopa* | 4,7% | 0,05 | 0,24 | 1,43 | 33,3% | 40,5% | 4,4% | 0,04 | 0,18 | 0,86 | 33,3% | 32,4% | 2,4% | 0,02 | 0,11 | 0,71 | 16,7% | 13,5% | 2,3% | 0,02 | 0,11 | 0,71 | 16,7% | 13,5% | - | - | - | - | - | - | 2,8% | 7 |
| *Pararge aegeria* | 9,3% | 0,07 | 0,21 | 0,71 | 8,5% | 5,1% | 2,2% | 0,02 | 0,13 | 0,86 | 2,1% | 1,5% | 33,3% | 0,37 | 0,62 | 2,14 | 29,8% | 28,2% | 27,9% | 0,22 | 0,37 | 1,43 | 25,5% | 16,7% | 39,0% | 0,66 | 1,06 | 5,00 | 34,0% | 48,5% | 22,0% | 67 |
| *Polygonia c-album* | 7,0% | 0,07 | 0,26 | 1,43 | 8,1% | 8,2% | 2,2% | 0,02 | 0,13 | 0,86 | 2,7% | 2,4% | 16,7% | 0,15 | 0,37 | 1,43 | 18,9% | 18,4% | 34,9% | 0,30 | 0,47 | 2,14 | 40,5% | 36,7% | 26,8% | 0,29 | 0,51 | 2,00 | 29,7% | 34,3% | 17,3% | 44 |
| *Vanessa atalanta* | 11,6% | 0,08 | 0,23 | 0,71 | 14,3% | 9,2% | 8,9% | 0,08 | 0,25 | 0,86 | 11,4% | 8,8% | 19,0% | 0,19 | 0,42 | 1,43 | 22,9% | 20,1% | 23,3% | 0,28 | 0,66 | 2,86 | 28,6% | 31,1% | 19,5% | 0,29 | 0,64 | 2,00 | 22,9% | 30,8% | 16,4% | 49 |
| *Vanessa cardui* | 37,2% | 1,61 | 4,08 | 22,14 | 24,2% | 26,2% | 28,9% | 1,31 | 3,17 | 12,00 | 19,7% | 22,3% | 38,1% | 1,07 | 1,98 | 7,14 | 24,2% | 17,0% | 25,6% | 0,56 | 1,18 | 5,00 | 16,7% | 9,2% | 24,4% | 1,63 | 7,05 | 45,00 | 15,2% | 25,3% | 30,8% | 330 |
| *Papilio machaon* | 2,3% | 0,02 | 0,11 | 0,71 | 10,0% | 6,2% | 2,2% | 0,02 | 0,13 | 0,86 | 10,0% | 7,4% | 16,7% | 0,22 | 0,60 | 2,86 | 70,0% | 80,2% | 2,3% | 0,02 | 0,11 | 0,71 | 10,0% | 6,2% | - | - | - | - | - | - | 4,7% | 16 |
| *Anthocharis cardamines* | 9,3% | 0,28 | 1,00 | 4,29 | 18,2% | 24,7% | 8,9% | 0,25 | 0,85 | 4,29 | 18,2% | 22,7% | 4,8% | 0,03 | 0,15 | 0,71 | 9,1% | 2,9% | 9,3% | 0,15 | 0,51 | 2,14 | 18,2% | 13,1% | 19,5% | 0,44 | 1,05 | 5,00 | 36,4% | 36,6% | 10,3% | 59 |
| *Colias hyale* | 7,0% | 0,05 | 0,18 | 0,71 | 37,5% | 31,3% | 6,7% | 0,06 | 0,22 | 0,86 | 37,5% | 37,5% | 4,8% | 0,05 | 0,24 | 1,43 | 25,0% | 31,3% | - | - | - | - | - | - | - | - | - | - | - | - | 3,7% | 9 |
| *Gonepteryx rhamni* | 37,2% | 0,71 | 1,63 | 8,57 | 22,2% | 21,5% | 35,6% | 0,67 | 1,38 | 6,86 | 22,2% | 21,0% | 31,0% | 0,88 | 2,15 | 9,29 | 18,1% | 26,0% | 25,6% | 0,30 | 0,59 | 2,14 | 15,3% | 9,0% | 39,0% | 0,78 | 1,33 | 6,00 | 22,2% | 22,4% | 33,6% | 180 |
| *Leptidea juvernica* | 2,3% | 0,02 | 0,11 | 0,71 | 11,1% | 9,4% | 6,7% | 0,06 | 0,22 | 0,86 | 33,3% | 34,0% | 9,5% | 0,09 | 0,28 | 1,43 | 44,4% | 47,2% | 2,3% | 0,02 | 0,11 | 0,71 | 11,1% | 9,4% | - | - | - | - | - | - | 4,2% | 10 |
| *Pieris brassicae* | 11,6% | 0,10 | 0,29 | 1,43 | 23,8% | 17,6% | 17,8% | 0,23 | 0,62 | 3,43 | 38,1% | 42,4% | 7,1% | 0,07 | 0,26 | 1,43 | 14,3% | 11,8% | 7,0% | 0,07 | 0,26 | 1,43 | 14,3% | 11,8% | 4,9% | 0,10 | 0,49 | 3,00 | 9,5% | 16,5% | 9,8% | 30 |
| *Pieris napi* | **62,8%** | **2,03** | **2,58** | **10,71** | **22,1%** | **20,1%** | 42,2% | 1,31 | 2,15 | 7,71 | 15,6% | 13,6% | **64,3%** | **2,50** | **3,10** | **13,57** | **22,1%** | **24,2%** | **53,5%** | **1,05** | **1,58** | **7,14** | **18,9%** | **10,4%** | **63,4%** | **3,37** | **3,58** | **13,00** | **21,3%** | **31,8%** | 57,0% | 539 |
| *Pieris rapae* | **72,1%** | **3,62** | **3,93** | **15,71** | **22,3%** | **29,8%** | 71,1% | 3,41 | 3,61 | 14,57 | 23,0% | 29,4% | **52,4%** | **1,17** | **1,48** | **5,00** | **15,8%** | **9,4%** | **76,7%** | **2,71** | **3,68** | **20,00** | **23,7%** | **22,3%** | **51,2%** | **1,15** | **1,35** | **5,00** | **15,1%** | **9,0%** | 65,0% | 676 |
| *Pontia edusa* | 4,65% | 0,05 | 0,24 | 1,43 | 7,7% | 3,9% | **46,7%** | **1,09** | **1,64** | **6,00** | **80,8%** | **89,1%** | - | - | - | - | - | - | 4,7% | 0,07 | 0,34 | 2,14 | 7,7% | 5,2% | 2,4% | 0,02 | 0,16 | 1,00 | 3,8% | 1,8% | 12,1% | 65 |
