# Supplementary material for: Life on Green Patches: Diversity and Seasonal Changes of Butterfly Communities Associated With Wastelands of the Post‐Industrial Central European City
Source: Ecol Evol. 2024 Dec 16;14(12):e70695. doi: 10.1002/ece3.70695 (PMC11650753; doi:10.1002/ece3.70695)
Supplement: Supplementary file 5 — Appendix S5. Species composition, frequency of occurrence (F), abundance (M—mean, SD—standard deviation, MAX—maximum value per single transect) and values of association indices (DAI and DAS) for each cluster derived from Bray–Curtis similarity dendrogram (clusters 1–4). [file ECE3-14-e70695-s010.docx]

Appendix 5. Species composition, frequency of occurrence (F), abundance (M – mean, SD – standard deviation, MAX – maximum value per single transect) and values of association indices (DAI and DAS) for each cluster derived from Bray-Curtis similarity dendrogram (clusters 1-4).

|  | Cluster 1 | | | | | | Cluster 2 | | | | | | Cluster 3 | | | | | | Cluster 4 | | | | | |
| --- | --- | --- | --- | --- | --- | --- | --- | --- | --- | --- | --- | --- | --- | --- | --- | --- | --- | --- | --- | --- | --- | --- | --- | --- |
|  | F | M | SD | MAX | DAS | DAI | F | M | SD | MAX | DAS | DAI | F | M | SD | MAX | DAS | DAI | F | M | SD | MAX | DAS | DAI |
| *Erynnis tages* | 13% | 0,18 | 0,51 | 1,43 | 4,2% | 4,0% | - | - | - | - | - | - | 7% | 0,09 | 0,36 | 2,14 | 12,5% | 10,0% | 23% | 0,62 | 1,72 | 7,71 | 20,8% | 38,4% |
| *Carcharodus alceae* | - | - | - | - | - | - | - | - | - | - | - | - | - | - | - | - | - | - | - | - | - | - | - | - |
| *Thymelicus lineola* | - | - | - | - | - | - | - | - | - | - | - | - | 39% | 0,57 | 0,98 | 5,00 | 44,4% | 30,6% | 50% | 1,80 | 3,06 | 12,00 | 30,6% | 52,0% |
| *Ochlodes sylvanus* | 25% | 0,18 | 0,33 | 0,71 | 5,6% | 1,5% | - | - | - | - | - | - | 41% | 0,81 | 1,47 | 7,00 | 47,2% | 34,3% | - | - | - | - | - | - |
| *Thymelicus sylvestris* | - | - | - | - | - | - | - | - | - | - | - | - | 54% | 1,13 | 1,68 | 8,00 | 78,6% | 87,8% | 14% | 0,15 | 0,42 | 1,71 | 10,7% | 6,3% |
| *Thecla betulae* | - | - | - | - | - | - | - | - | - | - | - | - | 2% | 0,02 | 0,11 | 0,71 | 7,7% | 4,7% | - | - | - | - | - | - |
| *Lycaena phlaeas* | - | - | - | - | - | - | 25% | 0,30 | 0,64 | 2,14 | 3,5% | 2,3% | 41% | 0,68 | 1,12 | 5,00 | 19,8% | 18,3% | 82% | 2,34 | 2,34 | 8,57 | 20,9% | 33,7% |
| *Celastrina argiolus* | - | - | - | - | - | - | - | - | - | - | - | - | 15% | 0,13 | 0,33 | 1,43 | 46,2% | 37,0% | 9% | 0,10 | 0,35 | 1,43 | 15,4% | 16,0% |
| *Polyommatus icarus* | - | - | - | - | - | - | 17% | 0,42 | 0,98 | 2,86 | 2,0% | 1,9% | 24% | 0,49 | 1,23 | 6,43 | 10,0% | 7,6% | 77% | 2,34 | 2,26 | 7,14 | 17,0% | 19,7% |
| *Cupido argiades* | - | - | - | - | - | - | 33% | 0,48 | 0,77 | 2,14 | 9,5% | 9,2% | 34% | 0,64 | 1,26 | 5,71 | 33,3% | 42,7% | 18% | 0,14 | 0,31 | 0,86 | 9,5% | 5,1% |
| *Lycaena dispar* | - | - | - | - | - | - | - | - | - | - | - | - | 5% | 0,03 | 0,16 | 0,71 | 10,5% | 8,3% | 5% | 0,04 | 0,18 | 0,86 | 5,3% | 5,0% |
| *Aricia agestis* | - | - | - | - | - | - | 25% | 0,24 | 0,47 | 1,43 | 4,7% | 2,2% | 22% | 0,50 | 1,78 | 11,00 | 14,1% | 15,8% | 91% | 2,82 | 2,03 | 7,71 | 31,3% | 47,6% |
| *Lycaena alciphron* | - | - | - | - | - | - | - | - | - | - | - | - | 24% | 0,27 | 0,56 | 2,57 | 71,4% | 70,3% | 5% | 0,04 | 0,18 | 0,86 | 7,1% | 5,4% |
| *Lycaena tityrus* | - | - | - | - | - | - | - | - | - | - | - | - | 12% | 0,30 | 1,04 | 6,00 | 6,9% | 5,8% | 86% | 3,54 | 3,54 | 12,86 | 26,4% | 36,1% |
| *Polyommatus coridon* | - | - | - | - | - | - | - | - | - | - | - | - | - | - | - | - | - | - | 77% | 18,04 | 17,82 | 55,71 | 81,0% | 95,5% |
| *Satyrium pruni* | - | - | - | - | - | - | - | - | - | - | - | - | - | - | - | - | - | - | - | - | - | - | - | - |
| *Satyrium w-album* | - | - | - | - | - | - | - | - | - | - | - | - | 2% | 0,02 | 0,11 | 0,71 | 100,0% | 100,0% | - | - | - | - | - | - |
| *Aglais io* | - | - | - | - | - | - | - | - | - | - | - | - | 17% | 0,20 | 0,49 | 2,14 | 12,1% | 7,6% | 23% | 0,37 | 0,84 | 3,43 | 8,6% | 7,6% |
| *Boloria dia* | - | - | - | - | - | - | - | - | - | - | - | - | 17% | 0,33 | 0,86 | 3,43 | 43,8% | 41,7% | 18% | 0,43 | 1,08 | 4,29 | 25,0% | 28,9% |
| *Araschnia levana* | 13% | 0,09 | 0,25 | 0,71 | 2,5% | 1,0% | - | - | - | - | - | - | 44% | 0,94 | 1,60 | 8,00 | 45,0% | 55,0% | 14% | 0,12 | 0,30 | 1,00 | 7,5% | 3,7% |
| *Issoria lathonia* | - | - | - | - | - | - | 17% | 0,18 | 0,44 | 1,43 | 4,2% | 2,5% | 7% | 0,10 | 0,39 | 2,14 | 6,3% | 4,7% | 45% | 1,28 | 1,81 | 5,14 | 20,8% | 32,9% |
| *Pararge aegeria* | - | - | - | - | - | - | 50% | 0,36 | 0,37 | 0,71 | 12,8% | 7,7% | 32% | 0,52 | 1,01 | 5,00 | 27,7% | 38,5% | - | - | - | - | - | - |
| *Coenonympha pamphilus* | 100% | 4,11 | 3,85 | 12,86 | 5,6% | 3,2% | 42% | 0,71 | 1,29 | 4,29 | 3,5% | 0,8% | 46% | 1,28 | 2,49 | 12,86 | 13,2% | 5,1% | 95% | 13,65 | 12,90 | 46,29 | 14,6% | 29,4% |
| *Polygonia c-album* | - | - | - | - | - | - | - | - | - | - | - | - | 34% | 0,29 | 0,42 | 1,43 | 37,8% | 33,9% | - | - | - | - | - | - |
| *Vanessa atalanta* | - | - | - | - | - | - | 33% | 0,48 | 0,88 | 2,86 | 11,4% | 14,7% | 27% | 0,29 | 0,54 | 2,00 | 31,4% | 30,0% | 9% | 0,08 | 0,25 | 0,86 | 5,7% | 4,4% |
| *Nymphalis antiopa* | - | - | - | - | - | - | - | - | - | - | - | - | 2% | 0,02 | 0,11 | 0,71 | 16,7% | 13,5% | 5% | 0,03 | 0,15 | 0,71 | 16,7% | 13,5% |
| *Aglais urticae* | - | - | - | - | - | - | - | - | - | - | - | - | - | - | - | - | - | - | - | - | - | - | - | - |
| *Lasiommata megera* | - | - | - | - | - | - | - | - | - | - | - | - | 5% | 0,04 | 0,19 | 1,00 | 33,3% | 26,1% | 5% | 0,12 | 0,55 | 2,57 | 16,7% | 39,1% |
| *Vanessa cardui* | - | - | - | - | - | - | 25% | 0,30 | 0,64 | 2,14 | 4,5% | 1,3% | 37% | 1,04 | 3,50 | 22,14 | 22,7% | 16,1% | 55% | 2,31 | 3,51 | 12,00 | 18,2% | 19,2% |
| *Apatura ilia* | - | - | - | - | - | - | - | - | - | - | - | - | 15% | 0,14 | 0,40 | 2,14 | 75,0% | 80,0% | - | - | - | - | - | - |
| *Aphantopus hyperantus* | - | - | - | - | - | - | - | - | - | - | - | - | 83% | 8,29 | 9,47 | 39,00 | 66,7% | 85,6% | 45% | 1,97 | 3,61 | 15,00 | 19,6% | 10,9% |
| *Argynnis paphia* | - | - | - | - | - | - | 8% | 0,06 | 0,21 | 0,71 | 7,7% | 2,3% | 15% | 0,48 | 1,97 | 12,14 | 46,2% | 64,7% | 5% | 0,04 | 0,18 | 0,86 | 7,7% | 2,8% |
| *Brenthis ino* | - | - | - | - | - | - | - | - | - | - | - | - | 2% | 0,02 | 0,16 | 1,00 | 50,0% | 50,0% | - | - | - | - | - | - |
| *Coenonympha glycerion* | - | - | - | - | - | - | - | - | - | - | - | - | 2% | 0,02 | 0,13 | 0,86 | 50,0% | 54,5% | - | - | - | - | - | - |
| *Maniola jurtina* | - | - | - | - | - | - | - | - | - | - | - | - | 98% | 22,69 | 24,36 | 86,00 | 42,1% | 76,0% | 86% | 3,99 | 3,88 | 11,14 | 20,0% | 7,2% |
| *Melanargia galathea* | - | - | - | - | - | - | - | - | - | - | - | - | 95% | 6,02 | 5,84 | 27,14 | 61,9% | 69,2% | 68% | 4,53 | 6,78 | 24,86 | 23,8% | 27,9% |
| *Melitaea cinxia* | - | - | - | - | - | - | - | - | - | - | - | - | - | - | - | - | - | - | - | - | - | - | - | - |
| *Papilio machaon* | - | - | - | - | - | - | - | - | - | - | - | - | 7% | 0,05 | 0,19 | 0,71 | 30,0% | 18,5% | 5% | 0,04 | 0,18 | 0,86 | 10,0% | 7,4% |
| *Pieris napi* | - | - | - | - | - | - | 75% | 1,61 | 1,73 | 5,71 | 7,4% | 4,4% | 78% | 3,64 | 3,01 | 10,00 | 26,2% | 34,4% | 59% | 1,86 | 2,57 | 7,71 | 10,7% | 9,4% |
| *Anthocharis cardamines* | - | - | - | - | - | - | - | - | - | - | - | - | - | - | - | - | - | - | - | - | - | - | - | - |
| *Pieris rapae* | - | - | - | - | - | - | 100% | 3,39 | 2,44 | 9,29 | 8,6% | 7,8% | 78% | 2,18 | 1,93 | 6,43 | 23,0% | 17,1% | 95% | 6,70 | 4,50 | 15,71 | 15,1% | 28,3% |
| *Gonepteryx rhamni* | - | - | - | - | - | - | 8% | 0,06 | 0,21 | 0,71 | 1,4% | 0,5% | 49% | 1,58 | 2,59 | 9,29 | 27,8% | 45,4% | 36% | 0,86 | 1,71 | 6,86 | 11,1% | 13,2% |
| *Pieris brassicae* | - | - | - | - | - | - | - | - | - | - | - | - | 17% | 0,17 | 0,41 | 1,71 | 33,3% | 28,2% | 18% | 0,16 | 0,34 | 0,86 | 19,0% | 14,1% |
| *Leptidea juvernica* | - | - | - | - | - | - | - | - | - | - | - | - | 2% | 0,02 | 0,11 | 0,71 | 11,1% | 9,4% | 9% | 0,08 | 0,25 | 0,86 | 22,2% | 22,6% |
| *Colias hyale* | - | - | - | - | - | - | 8% | 0,06 | 0,21 | 0,71 | 12,5% | 10,4% | 2% | 0,03 | 0,22 | 1,43 | 12,5% | 20,8% | 5% | 0,04 | 0,18 | 0,86 | 12,5% | 12,5% |
| *Pontia edusa* | - | - | - | - | - | - | - | - | - | - | - | - | 2% | 0,02 | 0,13 | 0,86 | 3,8% | 1,6% | 77% | 1,97 | 1,90 | 6,00 | 65,4% | 79,2% |
