# Supplementary material for: Life on Green Patches: Diversity and Seasonal Changes of Butterfly Communities Associated With Wastelands of the Post‐Industrial Central European City
Source: Ecol Evol. 2024 Dec 16;14(12):e70695. doi: 10.1002/ece3.70695 (PMC11650753; doi:10.1002/ece3.70695)
Supplement: Supplementary file 7 — Appendix S7. SIMPER report. [file ECE3-14-e70695-s008.docx]

Appendix 7. SIMPER raport

| SIMPER  Similarity Percentages - species contributions  Sample selection: All  Variable selection: All  *Parameters*  Standardise data: No  Transform: Square root  Cut off for low contributions: 95,00%  Factor name: groups  *Factor groups*  7  8  2  6  3  4  5  1  *Group 7*  Average similarity: 30,79  Species Av.Abund Av.Sim Sim/SD Contrib% Cum.%  Aglais_io 1,82 8,27 0,96 26,85 26,85  Pieris_napi 2,01 7,03 0,81 22,82 49,68  Gonepteryx_rhamni 0,87 3,39 0,59 11,00 60,68  Pieris_rapae 0,95 3,13 0,54 10,18 70,86  Anthocharis_cardamines 1,05 2,55 0,47 8,29 79,15  Lycaena_phlaeas 0,49 1,25 0,38 4,06 83,21  Erynnis_tages 0,32 0,96 0,24 3,12 86,33  Pararge_aegeria 0,35 0,81 0,29 2,61 88,95  Issoria_lathonia 0,50 0,79 0,29 2,55 91,50  Coenonympha_pamphilus 0,36 0,61 0,20 1,97 93,47  Polygonia_c-album 0,25 0,47 0,22 1,53 94,99  Araschnia_levana 0,41 0,32 0,19 1,03 96,02  *Group 8*  Average similarity: 18,45  Species Av.Abund Av.Sim Sim/SD Contrib% Cum.%  Gonepteryx_rhamni 1,10 10,41 0,58 56,43 56,43  Polygonia_c-album 0,49 3,96 0,39 21,44 77,87  Vanessa_cardui 0,41 1,86 0,22 10,08 87,95  Aglais_urticae 0,27 1,32 0,22 7,15 95,10  *Group 2*  Average similarity: 44,78  Species Av.Abund Av.Sim Sim/SD Contrib% Cum.%  Pieris_rapae 3,39 24,77 3,21 55,32 55,32  Pieris_napi 1,61 9,57 0,99 21,38 76,71  Pararge_aegeria 0,36 4,00 0,52 8,93 85,64  Coenonympha_pamphilus 0,71 1,91 0,40 4,27 89,92  Cupido_argiades 0,48 1,30 0,31 2,90 92,82  Vanessa_atalanta 0,48 1,30 0,29 2,90 95,72  *Group 6*  Average similarity: 43,11  Species Av.Abund Av.Sim Sim/SD Contrib% Cum.%  Coenonympha_pamphilus 8,08 22,96 2,60 53,25 53,25  Lycaena_tityrus 2,00 4,54 0,68 10,54 63,79  Vanessa_cardui 3,91 4,38 0,57 10,16 73,95  Polyommatus_icarus 0,96 3,97 0,69 9,21 83,16  Ochlodes_sylvanus 1,88 3,64 0,55 8,44 91,61  Maniola_jurtina 0,82 0,64 0,23 1,48 93,08  Pieris_rapae 0,20 0,46 0,24 1,07 94,15  Pieris_napi 0,39 0,39 0,19 0,89 95,04  *Group 3*  Average similarity: 46,84  Species Av.Abund Av.Sim Sim/SD Contrib% Cum.%  Maniola_jurtina 22,69 12,47 2,07 26,62 26,62  Melanargia_galathea 6,02 7,64 1,93 16,31 42,94  Aphantopus_hyperantus 8,29 6,89 1,22 14,71 57,64  Pieris_napi 3,64 4,87 1,15 10,40 68,05  Pieris_rapae 2,18 4,15 1,01 8,85 76,90  Thymelicus_sylvestris 1,13 1,48 0,60 3,15 80,05  Gonepteryx_rhamni 1,58 1,29 0,50 2,75 82,80  Coenonympha_pamphilus 1,28 1,20 0,46 2,57 85,37  Ochlodes_sylvanus 0,81 1,00 0,41 2,15 87,52  Araschnia_levana 0,94 0,77 0,46 1,64 89,16  Lycaena_phlaeas 0,68 0,74 0,42 1,58 90,74  Thymelicus_lineola 0,57 0,69 0,40 1,48 92,21  Vanessa_cardui 1,04 0,60 0,37 1,28 93,49  Cupido_argiades 0,64 0,58 0,33 1,25 94,74  Polygonia_c-album 0,29 0,49 0,34 1,04 95,78  *Group 4*  Average similarity: 52,91  Species Av.Abund Av.Sim Sim/SD Contrib% Cum.%  Coenonympha_pamphilus 13,65 7,57 1,33 14,31 14,31  Polyommatus_coridon 18,04 7,33 0,99 13,86 28,17  Pieris_rapae 6,70 7,10 2,24 13,43 41,60  Aricia_agestis 2,82 4,38 1,96 8,28 49,87  Maniola_jurtina 3,99 4,18 1,38 7,91 57,78  Lycaena_tityrus 3,54 3,82 1,41 7,22 65,00  Lycaena_phlaeas 2,34 3,02 1,17 5,70 70,70  Polyommatus_icarus 2,34 2,86 1,06 5,41 76,12  Melanargia_galathea 4,53 2,73 0,79 5,16 81,28  Pontia_edusa 1,97 2,63 1,07 4,97 86,25  Vanessa_cardui 2,31 1,72 0,54 3,25 89,49  Pieris_napi 1,86 1,52 0,65 2,87 92,36  Thymelicus_lineola 1,80 1,10 0,53 2,08 94,44  Aphantopus_hyperantus 1,97 1,06 0,44 2,01 96,45  *Group 5*  Average similarity: 46,91  Species Av.Abund Av.Sim Sim/SD Contrib% Cum.%  Coenonympha_pamphilus 6,80 12,55 2,10 26,77 26,77  Pieris_rapae 3,92 9,99 1,55 21,29 48,06  Polyommatus_icarus 2,87 7,30 1,56 15,57 63,62  Pieris_napi 2,58 4,42 0,80 9,42 73,05  Maniola_jurtina 3,51 2,71 0,58 5,78 78,82  Lycaena_phlaeas 0,90 2,45 0,60 5,21 84,04  Lycaena_tityrus 1,16 2,18 0,61 4,66 88,69  Aricia_agestis 0,71 1,58 0,54 3,37 92,06  Issoria_lathonia 0,51 0,68 0,33 1,45 93,51  Vanessa_cardui 0,69 0,59 0,29 1,26 94,77  Vanessa_atalanta 0,28 0,39 0,25 0,83 95,60  *Group 1*  Average similarity: 64,07  Species Av.Abund Av.Sim Sim/SD Contrib% Cum.%  Coenonympha_pamphilus 4,11 62,29 2,59 97,21 97,21  *Groups 7 & 8*  Average dissimilarity = 88,05  Group 7 Group 8  Species Av.Abund Av.Abund Av.Diss Diss/SD Contrib% Cum.%  Pieris_napi 2,01 0,00 10,63 1,22 12,07 12,07  Aglais_io 1,82 0,24 10,24 1,10 11,62 23,69  Gonepteryx_rhamni 0,87 1,10 8,75 0,96 9,94 33,63  Pieris_rapae 0,95 0,00 6,27 0,89 7,12 40,74  Anthocharis_cardamines 1,05 0,00 5,81 0,83 6,60 47,35  Polygonia_c-album 0,25 0,49 5,55 0,77 6,30 53,65  Issoria_lathonia 0,50 0,24 4,17 0,67 4,73 58,38  Vanessa_cardui 0,02 0,41 3,91 0,55 4,44 62,82  Erynnis_tages 0,32 0,00 3,57 0,50 4,05 66,87  Lycaena_phlaeas 0,49 0,00 3,32 0,72 3,77 70,64  Aglais_urticae 0,02 0,27 3,11 0,55 3,53 74,17  Coenonympha_pamphilus 0,36 0,00 2,81 0,48 3,19 77,36  Pararge_aegeria 0,35 0,00 2,74 0,59 3,11 80,47  Vanessa_atalanta 0,10 0,20 2,26 0,48 2,56 83,03  Nymphalis_antiopa 0,06 0,12 2,12 0,40 2,41 85,44  Papilio_machaon 0,20 0,00 2,00 0,32 2,28 87,71  Araschnia_levana 0,41 0,00 1,89 0,47 2,15 89,86  Cupido_argiades 0,15 0,00 1,64 0,44 1,86 91,73  Polyommatus_icarus 0,19 0,00 1,54 0,40 1,75 93,48  Pieris_brassicae 0,21 0,00 1,25 0,40 1,42 94,90  Celastrina_argiolus 0,17 0,00 1,12 0,37 1,27 96,18  *Groups 7 & 2*  Average dissimilarity = 76,10  Group 7 Group 2  Species Av.Abund Av.Abund Av.Diss Diss/SD Contrib% Cum.%  Pieris_rapae 0,95 3,39 10,51 1,22 13,82 13,82  Aglais_io 1,82 0,00 8,04 1,35 10,56 24,38  Pieris_napi 2,01 1,61 7,31 1,27 9,61 33,98  Gonepteryx_rhamni 0,87 0,06 4,68 0,98 6,15 40,13  Coenonympha_pamphilus 0,36 0,71 4,57 0,82 6,00 46,14  Anthocharis_cardamines 1,05 0,00 4,44 0,84 5,83 51,97  Pararge_aegeria 0,35 0,36 4,10 0,92 5,39 57,36  Lycaena_phlaeas 0,49 0,30 3,66 0,78 4,81 62,17  Vanessa_atalanta 0,10 0,48 3,44 0,59 4,52 66,69  Cupido_argiades 0,15 0,48 3,38 0,76 4,44 71,13  Issoria_lathonia 0,50 0,18 2,99 0,73 3,93 75,06  Polyommatus_icarus 0,19 0,42 2,57 0,58 3,38 78,44  Erynnis_tages 0,32 0,00 2,45 0,53 3,22 81,66  Vanessa_cardui 0,02 0,30 1,87 0,56 2,46 84,12  Aricia_agestis 0,00 0,24 1,61 0,53 2,12 86,24  Polygonia_c-album 0,25 0,00 1,58 0,52 2,08 88,32  Araschnia_levana 0,41 0,00 1,53 0,47 2,01 90,32  Papilio_machaon 0,20 0,00 1,37 0,33 1,80 92,13  Pieris_brassicae 0,21 0,00 1,00 0,40 1,32 93,45  Celastrina_argiolus 0,17 0,00 0,89 0,37 1,16 94,61  Boloria_dia 0,22 0,00 0,76 0,28 1,00 95,61  *Groups 8 & 2*  Average dissimilarity = 96,01  Group 8 Group 2  Species Av.Abund Av.Abund Av.Diss Diss/SD Contrib% Cum.%  Pieris_rapae 0,00 3,39 22,29 2,49 23,22 23,22  Pieris_napi 0,00 1,61 12,25 1,39 12,76 35,98  Gonepteryx_rhamni 1,10 0,06 8,88 1,00 9,25 45,23  Pararge_aegeria 0,00 0,36 6,14 0,90 6,39 51,62  Vanessa_atalanta 0,20 0,48 5,77 0,64 6,01 57,63  Coenonympha_pamphilus 0,00 0,71 5,47 0,72 5,70 63,33  Vanessa_cardui 0,41 0,30 5,43 0,79 5,65 68,98  Polygonia_c-album 0,49 0,00 5,24 0,79 5,46 74,45  Cupido_argiades 0,00 0,48 4,17 0,67 4,34 78,79  Issoria_lathonia 0,24 0,18 3,51 0,58 3,66 82,44  Aglais_urticae 0,27 0,00 3,25 0,59 3,38 85,82  Lycaena_phlaeas 0,00 0,30 3,09 0,47 3,22 89,04  Aglais_io 0,24 0,00 2,66 0,61 2,78 91,82  Polyommatus_icarus 0,00 0,42 2,40 0,44 2,50 94,32  Aricia_agestis 0,00 0,24 2,27 0,54 2,36 96,68  *Groups 7 & 6*  Average dissimilarity = 87,59  Group 7 Group 6  Species Av.Abund Av.Abund Av.Diss Diss/SD Contrib% Cum.%  Coenonympha_pamphilus 0,36 8,08 15,14 1,87 17,29 17,29  Vanessa_cardui 0,02 3,91 7,03 0,88 8,03 25,31  Lycaena_tityrus 0,00 2,00 6,11 0,94 6,98 32,29  Pieris_napi 2,01 0,39 5,78 1,25 6,60 38,89  Aglais_io 1,82 0,26 5,72 1,30 6,53 45,42  Ochlodes_sylvanus 0,00 1,88 5,54 0,87 6,32 51,74  Polyommatus_icarus 0,19 0,96 4,57 1,02 5,22 56,96  Anthocharis_cardamines 1,05 0,22 3,78 0,90 4,31 61,27  Pieris_rapae 0,95 0,20 3,77 1,02 4,30 65,58  Gonepteryx_rhamni 0,87 0,22 3,68 1,03 4,20 69,78  Lycaena_phlaeas 0,49 0,12 2,51 0,81 2,86 72,64  Maniola_jurtina 0,00 0,82 2,32 0,51 2,65 75,29  Issoria_lathonia 0,50 0,10 2,16 0,67 2,46 77,75  Araschnia_levana 0,41 0,21 2,05 0,64 2,34 80,09  Pararge_aegeria 0,35 0,09 1,91 0,68 2,18 82,27  Erynnis_tages 0,32 0,00 1,81 0,54 2,06 84,33  Polygonia_c-album 0,25 0,04 1,44 0,58 1,64 85,97  Aricia_agestis 0,00 0,26 1,25 0,50 1,43 87,40  Lycaena_dispar 0,02 0,18 1,16 0,51 1,32 88,73  Pieris_brassicae 0,21 0,13 1,14 0,47 1,30 90,03  Cupido_argiades 0,15 0,09 1,14 0,48 1,30 91,33  Papilio_machaon 0,20 0,00 1,00 0,34 1,15 92,47  Leptidea_juvernica 0,07 0,04 0,74 0,35 0,84 93,32  Boloria_dia 0,22 0,03 0,72 0,32 0,83 94,15  Celastrina_argiolus 0,17 0,00 0,71 0,37 0,81 94,96  Lycaena_alciphron 0,00 0,12 0,54 0,29 0,62 95,57  *Groups 8 & 6*  Average dissimilarity = 94,54  Group 8 Group 6  Species Av.Abund Av.Abund Av.Diss Diss/SD Contrib% Cum.%  Coenonympha_pamphilus 0,00 8,08 23,06 2,51 24,39 24,39  Vanessa_cardui 0,41 3,91 9,57 1,04 10,13 34,52  Lycaena_tityrus 0,00 2,00 8,42 0,97 8,90 43,42  Ochlodes_sylvanus 0,00 1,88 7,53 0,90 7,97 51,39  Polyommatus_icarus 0,00 0,96 6,48 1,04 6,85 58,24  Gonepteryx_rhamni 1,10 0,22 6,04 1,02 6,39 64,63  Polygonia_c-album 0,49 0,04 3,64 0,83 3,85 68,48  Maniola_jurtina 0,00 0,82 3,12 0,52 3,30 71,77  Aglais_io 0,24 0,26 2,99 0,75 3,16 74,93  Aglais_urticae 0,27 0,00 2,22 0,61 2,35 77,28  Pieris_napi 0,00 0,39 2,18 0,47 2,31 79,59  Issoria_lathonia 0,24 0,10 2,08 0,50 2,20 81,80  Pieris_rapae 0,00 0,20 1,78 0,56 1,88 83,68  Aricia_agestis 0,00 0,26 1,68 0,51 1,78 85,46  Araschnia_levana 0,00 0,21 1,55 0,44 1,64 87,10  Lycaena_dispar 0,00 0,18 1,47 0,50 1,55 88,65  Lycaena_phlaeas 0,00 0,12 1,37 0,41 1,45 90,10  Anthocharis_cardamines 0,00 0,22 1,30 0,36 1,38 91,48  Vanessa_atalanta 0,20 0,00 1,28 0,40 1,36 92,84  Nymphalis_antiopa 0,12 0,00 1,21 0,39 1,28 94,11  Lycaena_alciphron 0,00 0,12 0,71 0,29 0,75 94,86  Pararge_aegeria 0,00 0,09 0,67 0,29 0,71 95,58  *Groups 2 & 6*  Average dissimilarity = 84,34  Group 2 Group 6  Species Av.Abund Av.Abund Av.Diss Diss/SD Contrib% Cum.%  Coenonympha_pamphilus 0,71 8,08 15,04 1,83 17,83 17,83  Pieris_rapae 3,39 0,20 10,18 2,00 12,07 29,90  Vanessa_cardui 0,30 3,91 7,55 0,98 8,96 38,85  Lycaena_tityrus 0,00 2,00 6,57 0,97 7,79 46,65  Pieris_napi 1,61 0,39 6,18 1,38 7,33 53,97  Ochlodes_sylvanus 0,00 1,88 5,95 0,90 7,05 61,03  Polyommatus_icarus 0,42 0,96 5,13 1,09 6,09 67,11  Pararge_aegeria 0,36 0,09 3,05 0,96 3,62 70,73  Vanessa_atalanta 0,48 0,00 2,54 0,60 3,01 73,75  Cupido_argiades 0,48 0,09 2,53 0,70 3,00 76,75  Maniola_jurtina 0,00 0,82 2,49 0,52 2,95 79,70  Lycaena_phlaeas 0,30 0,12 2,25 0,64 2,67 82,37  Aricia_agestis 0,24 0,26 2,21 0,74 2,62 84,99  Gonepteryx_rhamni 0,06 0,22 1,55 0,58 1,83 86,82  Issoria_lathonia 0,18 0,10 1,50 0,53 1,77 88,60  Aglais_io 0,00 0,26 1,43 0,49 1,70 90,29  Araschnia_levana 0,00 0,21 1,22 0,45 1,44 91,74  Lycaena_dispar 0,00 0,18 1,18 0,50 1,40 93,13  Anthocharis_cardamines 0,00 0,22 1,03 0,36 1,22 94,36  Colias_hyale 0,06 0,05 0,67 0,39 0,80 95,16  *Groups 7 & 3*  Average dissimilarity = 83,92  Group 7 Group 3  Species Av.Abund Av.Abund Av.Diss Diss/SD Contrib% Cum.%  Maniola_jurtina 0,00 22,69 13,88 2,16 16,54 16,54  Aphantopus_hyperantus 0,00 8,29 8,40 1,59 10,02 26,56  Melanargia_galathea 0,00 6,02 7,96 2,11 9,49 36,05  Pieris_napi 2,01 3,64 4,54 1,41 5,41 41,46  Pieris_rapae 0,95 2,18 4,02 1,10 4,79 46,25  Aglais_io 1,82 0,20 3,76 1,23 4,48 50,73  Gonepteryx_rhamni 0,87 1,58 3,30 1,09 3,93 54,66  Coenonympha_pamphilus 0,36 1,28 2,91 0,81 3,47 58,12  Thymelicus_sylvestris 0,00 1,13 2,60 0,95 3,10 61,22  Ochlodes_sylvanus 0,00 0,81 2,33 0,69 2,78 64,01  Anthocharis_cardamines 1,05 0,00 2,33 0,81 2,77 66,78  Lycaena_phlaeas 0,49 0,68 2,22 0,95 2,65 69,43  Araschnia_levana 0,41 0,94 2,15 0,92 2,57 72,00  Cupido_argiades 0,15 0,64 1,94 0,71 2,31 74,30  Vanessa_cardui 0,02 1,04 1,81 0,61 2,16 76,47  Pararge_aegeria 0,35 0,52 1,79 0,86 2,14 78,60  Thymelicus_lineola 0,02 0,57 1,67 0,74 1,99 80,59  Polygonia_c-album 0,25 0,29 1,55 0,81 1,85 82,44  Issoria_lathonia 0,50 0,10 1,39 0,64 1,65 84,10  Polyommatus_icarus 0,19 0,49 1,39 0,65 1,65 85,75  Erynnis_tages 0,32 0,09 1,25 0,59 1,48 87,23  Boloria_dia 0,22 0,33 1,16 0,50 1,38 88,62  Vanessa_atalanta 0,10 0,29 1,07 0,65 1,28 89,89  Pieris_brassicae 0,21 0,17 1,03 0,58 1,23 91,12  Lycaena_alciphron 0,00 0,27 0,98 0,53 1,17 92,28  Celastrina_argiolus 0,17 0,13 0,95 0,51 1,14 93,42  Aricia_agestis 0,00 0,50 0,94 0,45 1,12 94,54  Papilio_machaon 0,20 0,05 0,76 0,40 0,90 95,44  *Groups 8 & 3*  Average dissimilarity = 94,54  Group 8 Group 3  Species Av.Abund Av.Abund Av.Diss Diss/SD Contrib% Cum.%  Maniola_jurtina 0,00 22,69 16,74 2,35 17,71 17,71  Aphantopus_hyperantus 0,00 8,29 10,25 1,64 10,85 28,55  Melanargia_galathea 0,00 6,02 9,75 2,20 10,32 38,87  Pieris_napi 0,00 3,64 6,95 1,57 7,36 46,22  Pieris_rapae 0,00 2,18 6,27 1,21 6,63 52,85  Gonepteryx_rhamni 1,10 1,58 4,31 1,10 4,56 57,41  Coenonympha_pamphilus 0,00 1,28 3,30 0,73 3,49 60,89  Thymelicus_sylvestris 0,00 1,13 3,17 0,96 3,35 64,24  Ochlodes_sylvanus 0,00 0,81 2,94 0,70 3,10 67,35  Vanessa_cardui 0,41 1,04 2,85 0,78 3,01 70,36  Polygonia_c-album 0,49 0,29 2,38 0,92 2,52 72,88  Cupido_argiades 0,00 0,64 2,14 0,62 2,27 75,15  Lycaena_phlaeas 0,00 0,68 2,10 0,74 2,22 77,36  Araschnia_levana 0,00 0,94 2,09 0,83 2,21 79,57  Thymelicus_lineola 0,00 0,57 2,02 0,73 2,14 81,71  Aglais_io 0,24 0,20 1,61 0,71 1,71 83,41  Vanessa_atalanta 0,20 0,29 1,54 0,67 1,63 85,04  Pararge_aegeria 0,00 0,52 1,48 0,63 1,57 86,61  Aglais_urticae 0,27 0,00 1,29 0,59 1,37 87,97  Lycaena_alciphron 0,00 0,27 1,20 0,54 1,27 89,25  Polyommatus_icarus 0,00 0,49 1,18 0,53 1,25 90,49  Aricia_agestis 0,00 0,50 1,12 0,45 1,18 91,67  Issoria_lathonia 0,24 0,10 1,10 0,46 1,16 92,84  Boloria_dia 0,00 0,33 1,02 0,42 1,08 93,92  Celastrina_argiolus 0,00 0,13 0,76 0,36 0,80 94,72  Pieris_brassicae 0,00 0,17 0,75 0,43 0,79 95,51  *Groups 2 & 3*  Average dissimilarity = 79,18  Group 2 Group 3  Species Av.Abund Av.Abund Av.Diss Diss/SD Contrib% Cum.%  Maniola_jurtina 0,00 22,69 14,62 2,27 18,46 18,46  Aphantopus_hyperantus 0,00 8,29 8,87 1,64 11,20 29,66  Melanargia_galathea 0,00 6,02 8,41 2,21 10,62 40,28  Pieris_napi 1,61 3,64 4,47 1,43 5,64 45,93  Pieris_rapae 3,39 2,18 3,63 1,23 4,58 50,51  Coenonympha_pamphilus 0,71 1,28 3,33 0,92 4,20 54,71  Gonepteryx_rhamni 0,06 1,58 3,01 0,85 3,80 58,51  Thymelicus_sylvestris 0,00 1,13 2,75 0,97 3,47 61,98  Ochlodes_sylvanus 0,00 0,81 2,48 0,70 3,13 65,11  Cupido_argiades 0,48 0,64 2,46 0,85 3,11 68,22  Vanessa_cardui 0,30 1,04 2,28 0,74 2,87 71,10  Lycaena_phlaeas 0,30 0,68 2,20 0,86 2,78 73,88  Pararge_aegeria 0,36 0,52 2,15 1,06 2,72 76,59  Vanessa_atalanta 0,48 0,29 1,95 0,75 2,47 79,06  Araschnia_levana 0,00 0,94 1,87 0,82 2,36 81,42  Thymelicus_lineola 0,00 0,57 1,75 0,74 2,21 83,63  Polyommatus_icarus 0,42 0,49 1,73 0,67 2,18 85,81  Aricia_agestis 0,24 0,50 1,57 0,67 1,98 87,79  Polygonia_c-album 0,00 0,29 1,26 0,68 1,59 89,38  Lycaena_alciphron 0,00 0,27 1,04 0,54 1,31 90,69  Boloria_dia 0,00 0,33 0,89 0,42 1,12 91,81  Issoria_lathonia 0,18 0,10 0,87 0,50 1,09 92,90  Argynnis_paphia 0,06 0,48 0,86 0,44 1,09 93,99  Aglais_io 0,00 0,20 0,70 0,43 0,88 94,87  Pieris_brassicae 0,00 0,17 0,65 0,43 0,82 95,69  *Groups 6 & 3*  Average dissimilarity = 82,77  Group 6 Group 3  Species Av.Abund Av.Abund Av.Diss Diss/SD Contrib% Cum.%  Maniola_jurtina 0,82 22,69 11,68 1,89 14,11 14,11  Aphantopus_hyperantus 0,00 8,29 7,68 1,64 9,27 23,38  Coenonympha_pamphilus 8,08 1,28 7,46 1,61 9,01 32,40  Melanargia_galathea 0,02 6,02 7,17 2,18 8,66 41,06  Pieris_napi 0,39 3,64 4,83 1,57 5,84 46,90  Vanessa_cardui 3,91 1,04 4,39 0,97 5,30 52,20  Pieris_rapae 0,20 2,18 4,07 1,26 4,92 57,12  Lycaena_tityrus 2,00 0,30 3,53 0,97 4,26 61,38  Ochlodes_sylvanus 1,88 0,81 3,45 1,07 4,16 65,54  Polyommatus_icarus 0,96 0,49 2,69 1,08 3,25 68,79  Gonepteryx_rhamni 0,22 1,58 2,67 0,90 3,23 72,02  Thymelicus_sylvestris 0,07 1,13 2,39 0,99 2,89 74,91  Araschnia_levana 0,21 0,94 1,89 0,92 2,28 77,19  Lycaena_phlaeas 0,12 0,68 1,73 0,84 2,09 79,28  Cupido_argiades 0,09 0,64 1,65 0,65 2,00 81,28  Thymelicus_lineola 0,00 0,57 1,51 0,74 1,82 83,10  Aricia_agestis 0,26 0,50 1,36 0,65 1,65 84,75  Pararge_aegeria 0,09 0,52 1,28 0,71 1,55 86,30  Aglais_io 0,26 0,20 1,17 0,64 1,41 87,70  Polygonia_c-album 0,04 0,29 1,14 0,72 1,38 89,08  Lycaena_alciphron 0,12 0,27 1,09 0,61 1,31 90,40  Boloria_dia 0,03 0,33 0,82 0,45 1,00 91,39  Vanessa_atalanta 0,00 0,29 0,81 0,58 0,98 92,37  Pieris_brassicae 0,13 0,17 0,76 0,49 0,91 93,28  Lycaena_dispar 0,18 0,03 0,74 0,54 0,89 94,18  Argynnis_paphia 0,00 0,48 0,59 0,35 0,72 94,89  Anthocharis_cardamines 0,22 0,00 0,56 0,35 0,68 95,57  *Groups 7 & 4*  Average dissimilarity = 85,81  Group 7 Group 4  Species Av.Abund Av.Abund Av.Diss Diss/SD Contrib% Cum.%  Polyommatus_coridon 0,00 18,04 10,14 1,42 11,82 11,82  Coenonympha_pamphilus 0,36 13,65 8,78 1,50 10,23 22,05  Pieris_rapae 0,95 6,70 5,68 1,62 6,62 28,67  Maniola_jurtina 0,00 3,99 5,11 1,65 5,96 34,63  Lycaena_tityrus 0,00 3,54 4,69 1,77 5,47 40,09  Melanargia_galathea 0,00 4,53 4,64 1,10 5,40 45,50  Aricia_agestis 0,00 2,82 4,55 2,49 5,30 50,79  Polyommatus_icarus 0,19 2,34 3,59 1,40 4,18 54,98  Vanessa_cardui 0,02 2,31 3,55 0,83 4,14 59,12  Pontia_edusa 0,02 1,97 3,43 1,36 4,00 63,11  Lycaena_phlaeas 0,49 2,34 3,32 1,29 3,87 66,98  Pieris_napi 2,01 1,86 3,22 1,25 3,75 70,74  Aglais_io 1,82 0,37 3,02 1,26 3,52 74,25  Aphantopus_hyperantus 0,00 1,97 2,79 0,69 3,25 77,50  Thymelicus_lineola 0,02 1,80 2,47 0,91 2,87 80,37  Gonepteryx_rhamni 0,87 0,86 2,28 1,06 2,65 83,03  Issoria_lathonia 0,50 1,28 2,17 1,01 2,53 85,56  Anthocharis_cardamines 1,05 0,00 1,90 0,82 2,22 87,78  Erynnis_tages 0,32 0,62 1,47 0,72 1,71 89,49  Boloria_dia 0,22 0,43 0,97 0,53 1,14 90,62  Araschnia_levana 0,41 0,12 0,97 0,59 1,13 91,75  Pararge_aegeria 0,35 0,00 0,90 0,62 1,05 92,81  Cupido_argiades 0,15 0,14 0,81 0,63 0,94 93,75  Pieris_brassicae 0,21 0,16 0,77 0,60 0,90 94,64  Polygonia_c-album 0,25 0,00 0,69 0,52 0,80 95,44  *Groups 8 & 4*  Average dissimilarity = 96,30  Group 8 Group 4  Species Av.Abund Av.Abund Av.Diss Diss/SD Contrib% Cum.%  Polyommatus_coridon 0,00 18,04 11,86 1,44 12,32 12,32  Coenonympha_pamphilus 0,00 13,65 11,01 1,63 11,43 23,75  Pieris_rapae 0,00 6,70 8,58 2,34 8,91 32,65  Maniola_jurtina 0,00 3,99 6,01 1,66 6,24 38,89  Lycaena_tityrus 0,00 3,54 5,49 1,83 5,70 44,59  Melanargia_galathea 0,00 4,53 5,46 1,11 5,67 50,26  Aricia_agestis 0,00 2,82 5,32 2,61 5,52 55,79  Lycaena_phlaeas 0,00 2,34 4,55 1,48 4,73 60,51  Polyommatus_icarus 0,00 2,34 4,44 1,47 4,61 65,13  Vanessa_cardui 0,41 2,31 4,14 0,89 4,30 69,42  Pontia_edusa 0,00 1,97 4,05 1,35 4,21 73,63  Pieris_napi 0,00 1,86 3,34 0,97 3,47 77,10  Aphantopus_hyperantus 0,00 1,97 3,31 0,69 3,43 80,53  Gonepteryx_rhamni 1,10 0,86 2,96 1,06 3,07 83,60  Thymelicus_lineola 0,00 1,80 2,88 0,92 2,99 86,60  Issoria_lathonia 0,24 1,28 2,33 0,93 2,42 89,01  Polygonia_c-album 0,49 0,00 1,59 0,79 1,65 90,67  Aglais_io 0,24 0,37 1,41 0,80 1,47 92,13  Erynnis_tages 0,00 0,62 0,99 0,47 1,03 93,17  Aglais_urticae 0,27 0,00 0,99 0,60 1,03 94,20  Boloria_dia 0,00 0,43 0,81 0,44 0,84 95,04  *Groups 2 & 4*  Average dissimilarity = 78,83  Group 2 Group 4  Species Av.Abund Av.Abund Av.Diss Diss/SD Contrib% Cum.%  Polyommatus_coridon 0,00 18,04 10,61 1,44 13,46 13,46  Coenonympha_pamphilus 0,71 13,65 8,70 1,45 11,03 24,50  Maniola_jurtina 0,00 3,99 5,36 1,68 6,79 31,29  Lycaena_tityrus 0,00 3,54 4,91 1,81 6,23 37,52  Melanargia_galathea 0,00 4,53 4,86 1,11 6,16 43,69  Aricia_agestis 0,24 2,82 4,20 1,99 5,32 49,01  Polyommatus_icarus 0,42 2,34 3,76 1,40 4,76 53,77  Lycaena_phlaeas 0,30 2,34 3,66 1,37 4,65 58,42  Vanessa_cardui 0,30 2,31 3,65 0,88 4,63 63,05  Pontia_edusa 0,00 1,97 3,62 1,38 4,59 67,65  Pieris_rapae 3,39 6,70 3,50 1,34 4,44 72,09  Pieris_napi 1,61 1,86 3,17 1,30 4,02 76,10  Aphantopus_hyperantus 0,00 1,97 2,93 0,70 3,71 79,82  Thymelicus_lineola 0,00 1,80 2,58 0,92 3,27 83,09  Issoria_lathonia 0,18 1,28 2,07 0,96 2,63 85,71  Gonepteryx_rhamni 0,06 0,86 1,57 0,71 1,99 87,71  Pararge_aegeria 0,36 0,00 1,43 0,94 1,82 89,52  Cupido_argiades 0,48 0,14 1,42 0,79 1,80 91,32  Vanessa_atalanta 0,48 0,08 1,33 0,66 1,69 93,01  Erynnis_tages 0,00 0,62 0,91 0,47 1,15 94,16  Aglais_io 0,00 0,37 0,77 0,53 0,98 95,14  *Groups 6 & 4*  Average dissimilarity = 72,62  Group 6 Group 4  Species Av.Abund Av.Abund Av.Diss Diss/SD Contrib% Cum.%  Polyommatus_coridon 0,00 18,04 9,46 1,44 13,02 13,02  Pieris_rapae 0,20 6,70 6,23 2,14 8,58 21,60  Coenonympha_pamphilus 8,08 13,65 5,42 1,57 7,47 29,07  Maniola_jurtina 0,82 3,99 4,30 1,52 5,92 34,99  Melanargia_galathea 0,02 4,53 4,28 1,11 5,90 40,89  Vanessa_cardui 3,91 2,31 4,04 1,10 5,56 46,45  Aricia_agestis 0,26 2,82 3,79 2,07 5,22 51,67  Lycaena_tityrus 2,00 3,54 3,44 1,41 4,73 56,40  Lycaena_phlaeas 0,12 2,34 3,36 1,44 4,63 61,03  Pontia_edusa 0,08 1,97 3,15 1,37 4,34 65,37  Polyommatus_icarus 0,96 2,34 2,75 1,34 3,78 69,15  Pieris_napi 0,39 1,86 2,67 1,04 3,68 72,83  Ochlodes_sylvanus 1,88 0,00 2,65 0,89 3,65 76,48  Aphantopus_hyperantus 0,00 1,97 2,58 0,70 3,55 80,03  Thymelicus_lineola 0,00 1,80 2,30 0,91 3,17 83,19  Issoria_lathonia 0,10 1,28 1,82 0,91 2,50 85,70  Gonepteryx_rhamni 0,22 0,86 1,54 0,79 2,12 87,82  Aglais_io 0,26 0,37 1,10 0,71 1,52 89,34  Erynnis_tages 0,00 0,62 0,82 0,47 1,13 90,48  Araschnia_levana 0,21 0,12 0,78 0,59 1,07 91,55  Boloria_dia 0,03 0,43 0,71 0,48 0,98 92,53  Lycaena_dispar 0,18 0,04 0,60 0,54 0,83 93,36  Pieris_brassicae 0,13 0,16 0,56 0,50 0,77 94,13  Cupido_argiades 0,09 0,14 0,56 0,48 0,77 94,89  Thymelicus_sylvestris 0,07 0,15 0,55 0,46 0,76 95,65  *Groups 3 & 4*  Average dissimilarity = 67,73  Group 3 Group 4  Species Av.Abund Av.Abund Av.Diss Diss/SD Contrib% Cum.%  Polyommatus_coridon 0,00 18,04 7,44 1,39 10,99 10,99  Coenonympha_pamphilus 1,28 13,65 5,87 1,41 8,66 19,65  Maniola_jurtina 22,69 3,99 5,69 1,33 8,40 28,06  Aphantopus_hyperantus 8,29 1,97 4,26 1,40 6,29 34,35  Melanargia_galathea 6,02 4,53 3,46 1,51 5,10 39,45  Lycaena_tityrus 0,30 3,54 3,28 1,60 4,85 44,30  Pieris_rapae 2,18 6,70 3,10 1,39 4,58 48,88  Aricia_agestis 0,50 2,82 3,04 1,96 4,49 53,36  Pieris_napi 3,64 1,86 2,78 1,41 4,11 57,47  Polyommatus_icarus 0,49 2,34 2,58 1,35 3,81 61,28  Vanessa_cardui 1,04 2,31 2,56 0,94 3,78 65,06  Pontia_edusa 0,02 1,97 2,51 1,38 3,70 68,76  Lycaena_phlaeas 0,68 2,34 2,38 1,29 3,51 72,27  Gonepteryx_rhamni 1,58 0,86 1,96 0,98 2,90 75,17  Thymelicus_lineola 0,57 1,80 1,91 1,07 2,82 77,99  Thymelicus_sylvestris 1,13 0,15 1,56 0,99 2,31 80,30  Issoria_lathonia 0,10 1,28 1,45 0,88 2,14 82,44  Ochlodes_sylvanus 0,81 0,00 1,30 0,71 1,92 84,36  Araschnia_levana 0,94 0,12 1,23 0,88 1,81 86,17  Cupido_argiades 0,64 0,14 1,12 0,73 1,65 87,81  Boloria_dia 0,33 0,43 0,90 0,61 1,32 89,14  Aglais_io 0,20 0,37 0,80 0,68 1,18 90,32  Pararge_aegeria 0,52 0,00 0,77 0,64 1,13 91,45  Erynnis_tages 0,09 0,62 0,75 0,53 1,11 92,56  Polygonia_c-album 0,29 0,00 0,70 0,69 1,03 93,59  Vanessa_atalanta 0,29 0,08 0,63 0,65 0,94 94,53  Lycaena_alciphron 0,27 0,04 0,61 0,57 0,90 95,43  *Groups 7 & 5*  Average dissimilarity = 79,61  Group 7 Group 5  Species Av.Abund Av.Abund Av.Diss Diss/SD Contrib% Cum.%  Coenonympha_pamphilus 0,36 6,80 10,30 1,74 12,94 12,94  Pieris_rapae 0,95 3,92 6,91 1,22 8,68 21,62  Polyommatus_icarus 0,19 2,87 6,59 1,65 8,28 29,90  Pieris_napi 2,01 2,58 5,52 1,24 6,93 36,83  Maniola_jurtina 0,00 3,51 5,05 0,89 6,34 43,18  Aglais_io 1,82 0,15 4,88 1,31 6,13 49,31  Lycaena_phlaeas 0,49 0,90 3,56 0,98 4,48 53,79  Lycaena_tityrus 0,00 1,16 3,49 0,92 4,38 58,17  Gonepteryx_rhamni 0,87 0,17 3,02 1,00 3,79 61,96  Anthocharis_cardamines 1,05 0,00 2,93 0,83 3,68 65,65  Aricia_agestis 0,00 0,71 2,64 0,89 3,32 68,97  Issoria_lathonia 0,50 0,51 2,62 0,80 3,29 72,26  Pararge_aegeria 0,35 0,25 1,98 0,80 2,49 74,75  Vanessa_cardui 0,02 0,69 1,91 0,60 2,40 77,15  Cupido_argiades 0,15 0,35 1,72 0,67 2,16 79,31  Erynnis_tages 0,32 0,08 1,63 0,59 2,04 81,35  Polygonia_c-album 0,25 0,16 1,52 0,66 1,91 83,26  Vanessa_atalanta 0,10 0,28 1,50 0,61 1,88 85,14  Araschnia_levana 0,41 0,09 1,35 0,54 1,70 86,84  Thecla_betulae 0,17 0,15 1,07 0,50 1,35 88,19  Thymelicus_lineola 0,02 0,25 0,96 0,43 1,21 89,40  Papilio_machaon 0,20 0,01 0,86 0,35 1,08 90,48  Pieris_brassicae 0,21 0,03 0,79 0,44 1,00 91,48  Lycaena_dispar 0,02 0,16 0,73 0,44 0,92 92,39  Aphantopus_hyperantus 0,00 0,27 0,72 0,38 0,91 93,30  Pontia_edusa 0,02 0,14 0,70 0,33 0,89 94,19  Polyommatus_coridon 0,00 0,37 0,70 0,27 0,87 95,06  *Groups 8 & 5*  Average dissimilarity = 95,78  Group 8 Group 5  Species Av.Abund Av.Abund Av.Diss Diss/SD Contrib% Cum.%  Coenonympha_pamphilus 0,00 6,80 14,76 2,22 15,41 15,41  Pieris_rapae 0,00 3,92 11,99 1,67 12,51 27,92  Polyommatus_icarus 0,00 2,87 9,17 1,96 9,57 37,50  Pieris_napi 0,00 2,58 7,55 1,11 7,88 45,38  Maniola_jurtina 0,00 3,51 6,28 0,92 6,56 51,94  Lycaena_phlaeas 0,00 0,90 4,76 0,92 4,96 56,90  Gonepteryx_rhamni 1,10 0,17 4,69 0,97 4,90 61,80  Lycaena_tityrus 0,00 1,16 4,48 0,93 4,68 66,48  Vanessa_cardui 0,41 0,69 3,56 0,83 3,72 70,20  Aricia_agestis 0,00 0,71 3,39 0,90 3,54 73,73  Polygonia_c-album 0,49 0,16 3,04 0,85 3,17 76,91  Issoria_lathonia 0,24 0,51 2,99 0,70 3,12 80,03  Vanessa_atalanta 0,20 0,28 2,26 0,66 2,36 82,39  Aglais_io 0,24 0,15 2,14 0,65 2,24 84,63  Aglais_urticae 0,27 0,00 1,75 0,60 1,83 86,45  Cupido_argiades 0,00 0,35 1,60 0,52 1,67 88,12  Pararge_aegeria 0,00 0,25 1,48 0,56 1,55 89,66  Thymelicus_lineola 0,00 0,25 1,17 0,41 1,22 90,88  Thecla_betulae 0,00 0,15 1,05 0,48 1,10 91,98  Nymphalis_antiopa 0,12 0,01 1,02 0,41 1,06 93,04  Aphantopus_hyperantus 0,00 0,27 0,89 0,39 0,93 93,97  Polyommatus_coridon 0,00 0,37 0,87 0,28 0,91 94,88  Lycaena_dispar 0,00 0,16 0,84 0,41 0,88 95,75  *Groups 2 & 5*  Average dissimilarity = 66,61  Group 2 Group 5  Species Av.Abund Av.Abund Av.Diss Diss/SD Contrib% Cum.%  Coenonympha_pamphilus 0,71 6,80 10,18 1,68 15,29 15,29  Polyommatus_icarus 0,42 2,87 7,00 1,71 10,50 25,79  Pieris_napi 1,61 2,58 5,53 1,26 8,31 34,10  Maniola_jurtina 0,00 3,51 5,35 0,91 8,04 42,13  Pieris_rapae 3,39 3,92 4,43 1,18 6,65 48,79  Lycaena_phlaeas 0,30 0,90 3,82 0,99 5,73 54,52  Lycaena_tityrus 0,00 1,16 3,72 0,94 5,58 60,10  Aricia_agestis 0,24 0,71 2,97 0,99 4,46 64,56  Vanessa_atalanta 0,48 0,28 2,64 0,76 3,96 68,53  Vanessa_cardui 0,30 0,69 2,63 0,79 3,95 72,47  Cupido_argiades 0,48 0,35 2,58 0,82 3,87 76,34  Pararge_aegeria 0,36 0,25 2,55 0,98 3,82 80,16  Issoria_lathonia 0,18 0,51 2,34 0,72 3,51 83,67  Gonepteryx_rhamni 0,06 0,17 1,12 0,55 1,68 85,35  Thymelicus_lineola 0,00 0,25 0,97 0,41 1,45 86,80  Thecla_betulae 0,00 0,15 0,87 0,48 1,31 88,11  Polygonia_c-album 0,00 0,16 0,80 0,43 1,20 89,31  Argynnis_paphia 0,06 0,18 0,78 0,43 1,18 90,49  Aglais_io 0,00 0,15 0,78 0,34 1,17 91,66  Aphantopus_hyperantus 0,00 0,27 0,76 0,38 1,15 92,81  Polyommatus_coridon 0,00 0,37 0,74 0,27 1,11 93,92  Lycaena_dispar 0,00 0,16 0,71 0,41 1,07 94,99  Melanargia_galathea 0,00 0,19 0,69 0,42 1,03 96,02  *Groups 6 & 5*  Average dissimilarity = 65,94  Group 6 Group 5  Species Av.Abund Av.Abund Av.Diss Diss/SD Contrib% Cum.%  Pieris_rapae 0,20 3,92 7,20 1,63 10,91 10,91  Coenonympha_pamphilus 8,08 6,80 5,41 1,23 8,20 19,12  Vanessa_cardui 3,91 0,69 5,31 0,98 8,06 27,18  Pieris_napi 0,39 2,58 4,98 1,15 7,56 34,73  Maniola_jurtina 0,82 3,51 4,93 1,01 7,47 42,21  Lycaena_tityrus 2,00 1,16 4,37 1,11 6,62 48,83  Polyommatus_icarus 0,96 2,87 4,36 1,40 6,61 55,44  Ochlodes_sylvanus 1,88 0,00 4,04 0,89 6,12 61,56  Lycaena_phlaeas 0,12 0,90 3,04 1,00 4,62 66,18  Aricia_agestis 0,26 0,71 2,50 1,00 3,80 69,97  Issoria_lathonia 0,10 0,51 1,82 0,67 2,76 72,74  Aglais_io 0,26 0,15 1,39 0,59 2,10 74,84  Gonepteryx_rhamni 0,22 0,17 1,29 0,69 1,96 76,79  Cupido_argiades 0,09 0,35 1,27 0,55 1,92 78,71  Pararge_aegeria 0,09 0,25 1,25 0,63 1,89 80,61  Lycaena_dispar 0,18 0,16 1,20 0,64 1,82 82,43  Vanessa_atalanta 0,00 0,28 1,12 0,54 1,71 84,14  Araschnia_levana 0,21 0,09 1,07 0,53 1,62 85,76  Polygonia_c-album 0,04 0,16 0,82 0,50 1,25 87,01  Thymelicus_lineola 0,00 0,25 0,80 0,42 1,22 88,23  Pontia_edusa 0,08 0,14 0,76 0,39 1,16 89,38  Thecla_betulae 0,00 0,15 0,73 0,48 1,11 90,49  Anthocharis_cardamines 0,22 0,00 0,70 0,36 1,06 91,55  Melanargia_galathea 0,02 0,19 0,69 0,46 1,04 92,59  Aphantopus_hyperantus 0,00 0,27 0,66 0,38 1,00 93,59  Polyommatus_coridon 0,00 0,37 0,63 0,27 0,95 94,54  Argynnis_paphia 0,00 0,18 0,44 0,31 0,67 95,21  *Groups 3 & 5*  Average dissimilarity = 71,15  Group 3 Group 5  Species Av.Abund Av.Abund Av.Diss Diss/SD Contrib% Cum.%  Maniola_jurtina 22,69 3,51 9,26 1,55 13,01 13,01  Aphantopus_hyperantus 8,29 0,27 6,55 1,54 9,21 22,22  Melanargia_galathea 6,02 0,19 6,07 1,91 8,53 30,75  Coenonympha_pamphilus 1,28 6,80 5,67 1,49 7,97 38,72  Polyommatus_icarus 0,49 2,87 4,03 1,53 5,67 44,39  Pieris_napi 3,64 2,58 3,73 1,29 5,24 49,63  Pieris_rapae 2,18 3,92 3,19 1,20 4,48 54,11  Gonepteryx_rhamni 1,58 0,17 2,35 0,88 3,30 57,41  Lycaena_tityrus 0,30 1,16 2,30 0,97 3,24 60,65  Lycaena_phlaeas 0,68 0,90 2,26 1,06 3,17 63,82  Thymelicus_sylvestris 1,13 0,01 2,12 0,96 2,97 66,79  Vanessa_cardui 1,04 0,69 2,05 0,80 2,89 69,68  Aricia_agestis 0,50 0,71 1,92 0,94 2,70 72,37  Ochlodes_sylvanus 0,81 0,00 1,84 0,70 2,58 74,95  Cupido_argiades 0,64 0,35 1,73 0,78 2,43 77,38  Araschnia_levana 0,94 0,09 1,59 0,86 2,24 79,62  Thymelicus_lineola 0,57 0,25 1,55 0,83 2,18 81,80  Pararge_aegeria 0,52 0,25 1,38 0,83 1,94 83,74  Issoria_lathonia 0,10 0,51 1,26 0,65 1,78 85,51  Vanessa_atalanta 0,29 0,28 1,20 0,75 1,69 87,20  Polygonia_c-album 0,29 0,16 1,15 0,78 1,61 88,82  Aglais_io 0,20 0,15 0,85 0,54 1,19 90,01  Argynnis_paphia 0,48 0,18 0,81 0,46 1,14 91,15  Lycaena_alciphron 0,27 0,00 0,79 0,54 1,11 92,26  Boloria_dia 0,33 0,00 0,68 0,42 0,96 93,22  Thecla_betulae 0,02 0,15 0,56 0,50 0,79 94,01  Pieris_brassicae 0,17 0,03 0,56 0,47 0,79 94,80  Lycaena_dispar 0,03 0,16 0,53 0,46 0,74 95,54  *Groups 4 & 5*  Average dissimilarity = 60,53  Group 4 Group 5  Species Av.Abund Av.Abund Av.Diss Diss/SD Contrib% Cum.%  Polyommatus_coridon 18,04 0,37 8,41 1,41 13,89 13,89  Coenonympha_pamphilus 13,65 6,80 5,11 1,41 8,45 22,34  Maniola_jurtina 3,99 3,51 3,83 1,42 6,32 28,66  Melanargia_galathea 4,53 0,19 3,77 1,10 6,23 34,89  Lycaena_tityrus 3,54 1,16 3,08 1,42 5,08 39,98  Pieris_rapae 6,70 3,92 3,00 1,26 4,95 44,93  Pieris_napi 1,86 2,58 2,97 1,22 4,91 49,83  Vanessa_cardui 2,31 0,69 2,93 0,94 4,84 54,67  Aricia_agestis 2,82 0,71 2,85 1,59 4,70 59,37  Pontia_edusa 1,97 0,14 2,81 1,37 4,65 64,02  Polyommatus_icarus 2,34 2,87 2,55 1,32 4,21 68,23  Lycaena_phlaeas 2,34 0,90 2,53 1,32 4,18 72,41  Aphantopus_hyperantus 1,97 0,27 2,40 0,75 3,96 76,37  Thymelicus_lineola 1,80 0,25 2,14 0,97 3,53 79,90  Issoria_lathonia 1,28 0,51 1,89 1,02 3,12 83,02  Gonepteryx_rhamni 0,86 0,17 1,37 0,77 2,26 85,28  Cupido_argiades 0,14 0,35 0,92 0,69 1,52 86,80  Erynnis_tages 0,62 0,08 0,87 0,54 1,43 88,23  Aglais_io 0,37 0,15 0,86 0,63 1,42 89,66  Vanessa_atalanta 0,08 0,28 0,77 0,61 1,27 90,92  Pararge_aegeria 0,00 0,25 0,63 0,56 1,04 91,96  Boloria_dia 0,43 0,00 0,61 0,45 1,01 92,96  Araschnia_levana 0,12 0,09 0,49 0,47 0,81 93,77  Thecla_betulae 0,00 0,15 0,44 0,48 0,73 94,50  Lycaena_dispar 0,04 0,16 0,44 0,46 0,72 95,22  *Groups 7 & 1*  Average dissimilarity = 93,97  Group 7 Group 1  Species Av.Abund Av.Abund Av.Diss Diss/SD Contrib% Cum.%  Coenonympha_pamphilus 0,36 4,11 19,87 1,37 21,14 21,14  Aglais_io 1,82 0,00 11,30 1,29 12,03 33,17  Pieris_napi 2,01 0,00 10,76 1,26 11,45 44,62  Pieris_rapae 0,95 0,00 6,35 0,90 6,76 51,38  Gonepteryx_rhamni 0,87 0,00 6,33 0,95 6,73 58,11  Anthocharis_cardamines 1,05 0,00 5,90 0,84 6,27 64,38  Erynnis_tages 0,32 0,18 4,29 0,59 4,56 68,95  Lycaena_phlaeas 0,49 0,00 3,37 0,72 3,58 72,53  Issoria_lathonia 0,50 0,00 2,88 0,60 3,07 75,60  Ochlodes_sylvanus 0,00 0,18 2,88 0,48 3,06 78,66  Pararge_aegeria 0,35 0,00 2,77 0,59 2,95 81,61  Araschnia_levana 0,41 0,09 2,54 0,59 2,70 84,31  Polygonia_c-album 0,25 0,00 2,07 0,52 2,20 86,51  Papilio_machaon 0,20 0,00 2,03 0,32 2,16 88,68  Cupido_argiades 0,15 0,00 1,67 0,44 1,77 90,45  Polyommatus_icarus 0,19 0,00 1,57 0,40 1,67 92,12  Pieris_brassicae 0,21 0,00 1,27 0,40 1,35 93,46  Celastrina_argiolus 0,17 0,00 1,14 0,37 1,21 94,68  Boloria_dia 0,22 0,00 0,95 0,29 1,01 95,68  *Groups 8 & 1*  Average dissimilarity = 100,00  Group 8 Group 1  Species Av.Abund Av.Abund Av.Diss Diss/SD Contrib% Cum.%  Coenonympha_pamphilus 0,00 4,11 41,06 2,37 41,06 41,06  Gonepteryx_rhamni 1,10 0,00 15,35 1,03 15,35 56,41  Polygonia_c-album 0,49 0,00 9,00 0,77 9,00 65,42  Vanessa_cardui 0,41 0,00 6,87 0,60 6,87 72,29  Ochlodes_sylvanus 0,00 0,18 5,62 0,53 5,62 77,91  Aglais_urticae 0,27 0,00 5,40 0,59 5,40 83,31  Aglais_io 0,24 0,00 3,98 0,62 3,98 87,29  Issoria_lathonia 0,24 0,00 3,39 0,40 3,39 90,69  Nymphalis_antiopa 0,12 0,00 3,36 0,40 3,36 94,04  Vanessa_atalanta 0,20 0,00 2,74 0,40 2,74 96,78  *Groups 2 & 1*  Average dissimilarity = 89,96  Group 2 Group 1  Species Av.Abund Av.Abund Av.Diss Diss/SD Contrib% Cum.%  Pieris_rapae 3,39 0,00 22,63 2,71 25,15 25,15  Coenonympha_pamphilus 0,71 4,11 19,19 1,59 21,33 46,49  Pieris_napi 1,61 0,00 12,44 1,43 13,83 60,31  Pararge_aegeria 0,36 0,00 6,23 0,92 6,92 67,24  Vanessa_atalanta 0,48 0,00 5,02 0,56 5,58 72,82  Cupido_argiades 0,48 0,00 4,23 0,67 4,71 77,52  Lycaena_phlaeas 0,30 0,00 3,14 0,48 3,49 81,01  Ochlodes_sylvanus 0,00 0,18 3,04 0,55 3,38 84,39  Vanessa_cardui 0,30 0,00 2,64 0,56 2,93 87,32  Polyommatus_icarus 0,42 0,00 2,44 0,44 2,71 90,03  Aricia_agestis 0,24 0,00 2,30 0,55 2,56 92,59  Issoria_lathonia 0,18 0,00 1,87 0,44 2,08 94,67  Erynnis_tages 0,00 0,18 1,35 0,37 1,50 96,17  *Groups 6 & 1*  Average dissimilarity = 69,03  Group 6 Group 1  Species Av.Abund Av.Abund Av.Diss Diss/SD Contrib% Cum.%  Coenonympha_pamphilus 8,08 4,11 10,39 1,38 15,04 15,04  Vanessa_cardui 3,91 0,00 9,55 0,93 13,83 28,88  Lycaena_tityrus 2,00 0,00 8,54 0,97 12,37 41,24  Ochlodes_sylvanus 1,88 0,18 7,67 1,02 11,11 52,35  Polyommatus_icarus 0,96 0,00 6,57 1,05 9,52 61,87  Maniola_jurtina 0,82 0,00 3,16 0,52 4,57 66,45  Pieris_napi 0,39 0,00 2,22 0,47 3,21 69,65  Araschnia_levana 0,21 0,09 2,04 0,56 2,96 72,61  Aglais_io 0,26 0,00 1,83 0,49 2,65 75,26  Pieris_rapae 0,20 0,00 1,81 0,56 2,62 77,88  Aricia_agestis 0,26 0,00 1,70 0,51 2,46 80,34  Gonepteryx_rhamni 0,22 0,00 1,49 0,51 2,16 82,50  Lycaena_dispar 0,18 0,00 1,49 0,50 2,15 84,66  Lycaena_phlaeas 0,12 0,00 1,39 0,41 2,02 86,67  Anthocharis_cardamines 0,22 0,00 1,32 0,36 1,92 88,59  Erynnis_tages 0,00 0,18 1,00 0,37 1,45 90,04  Issoria_lathonia 0,10 0,00 0,82 0,31 1,19 91,23  Lycaena_alciphron 0,12 0,00 0,72 0,29 1,04 92,27  Pararge_aegeria 0,09 0,00 0,68 0,29 0,99 93,26  Thymelicus_sylvestris 0,07 0,00 0,63 0,25 0,91 94,17  Melitaea_cinxia 0,10 0,00 0,53 0,25 0,77 94,94  Pieris_brassicae 0,13 0,00 0,52 0,25 0,76 95,70  *Groups 3 & 1*  Average dissimilarity = 93,28  Group 3 Group 1  Species Av.Abund Av.Abund Av.Diss Diss/SD Contrib% Cum.%  Maniola_jurtina 22,69 0,00 16,89 2,37 18,11 18,11  Aphantopus_hyperantus 8,29 0,00 10,35 1,64 11,10 29,21  Melanargia_galathea 6,02 0,00 9,85 2,21 10,56 39,76  Pieris_napi 3,64 0,00 7,02 1,58 7,53 47,29  Coenonympha_pamphilus 1,28 4,11 6,95 1,34 7,45 54,73  Pieris_rapae 2,18 0,00 6,33 1,21 6,79 61,52  Gonepteryx_rhamni 1,58 0,00 3,46 0,82 3,71 65,23  Thymelicus_sylvestris 1,13 0,00 3,20 0,96 3,43 68,66  Ochlodes_sylvanus 0,81 0,18 3,07 0,79 3,29 71,95  Araschnia_levana 0,94 0,09 2,24 0,90 2,40 74,36  Vanessa_cardui 1,04 0,00 2,18 0,61 2,34 76,70  Cupido_argiades 0,64 0,00 2,16 0,62 2,32 79,02  Lycaena_phlaeas 0,68 0,00 2,12 0,74 2,27 81,28  Thymelicus_lineola 0,57 0,00 2,04 0,74 2,19 83,47  Pararge_aegeria 0,52 0,00 1,49 0,63 1,60 85,07  Polygonia_c-album 0,29 0,00 1,48 0,67 1,59 86,66  Lycaena_alciphron 0,27 0,00 1,21 0,54 1,30 87,96  Polyommatus_icarus 0,49 0,00 1,19 0,53 1,27 89,23  Aricia_agestis 0,50 0,00 1,13 0,45 1,21 90,44  Vanessa_atalanta 0,29 0,00 1,04 0,57 1,12 91,56  Boloria_dia 0,33 0,00 1,03 0,42 1,10 92,67  Erynnis_tages 0,09 0,18 0,81 0,44 0,87 93,54  Aglais_io 0,20 0,00 0,81 0,43 0,87 94,41  Celastrina_argiolus 0,13 0,00 0,77 0,37 0,82 95,23  *Groups 4 & 1*  Average dissimilarity = 89,11  Group 4 Group 1  Species Av.Abund Av.Abund Av.Diss Diss/SD Contrib% Cum.%  Polyommatus_coridon 18,04 0,00 11,95 1,44 13,41 13,41  Pieris_rapae 6,70 0,00 8,64 2,35 9,70 23,11  Coenonympha_pamphilus 13,65 4,11 7,31 1,45 8,20 31,31  Maniola_jurtina 3,99 0,00 6,05 1,66 6,80 38,11  Lycaena_tityrus 3,54 0,00 5,53 1,83 6,21 44,32  Melanargia_galathea 4,53 0,00 5,50 1,11 6,18 50,49  Aricia_agestis 2,82 0,00 5,36 2,62 6,01 56,51  Lycaena_phlaeas 2,34 0,00 4,59 1,48 5,15 61,66  Polyommatus_icarus 2,34 0,00 4,48 1,47 5,03 66,68  Vanessa_cardui 2,31 0,00 4,32 0,83 4,85 71,53  Pontia_edusa 1,97 0,00 4,08 1,35 4,58 76,11  Pieris_napi 1,86 0,00 3,37 0,97 3,78 79,89  Aphantopus_hyperantus 1,97 0,00 3,33 0,69 3,74 83,63  Thymelicus_lineola 1,80 0,00 2,91 0,92 3,26 86,89  Issoria_lathonia 1,28 0,00 2,13 0,86 2,39 89,28  Gonepteryx_rhamni 0,86 0,00 1,65 0,65 1,85 91,13  Erynnis_tages 0,62 0,18 1,33 0,59 1,50 92,63  Aglais_io 0,37 0,00 0,86 0,53 0,97 93,59  Boloria_dia 0,43 0,00 0,82 0,45 0,92 94,51  Ochlodes_sylvanus 0,00 0,18 0,81 0,55 0,91 95,42  *Groups 5 & 1*  Average dissimilarity = 80,28  Group 5 Group 1  Species Av.Abund Av.Abund Av.Diss Diss/SD Contrib% Cum.%  Pieris_rapae 3,92 0,00 12,14 1,69 15,12 15,12  Polyommatus_icarus 2,87 0,00 9,28 1,98 11,56 26,68  Coenonympha_pamphilus 6,80 4,11 7,71 1,35 9,60 36,28  Pieris_napi 2,58 0,00 7,64 1,12 9,51 45,79  Maniola_jurtina 3,51 0,00 6,35 0,92 7,91 53,70  Lycaena_phlaeas 0,90 0,00 4,82 0,93 6,00 59,70  Lycaena_tityrus 1,16 0,00 4,54 0,94 5,65 65,36  Aricia_agestis 0,71 0,00 3,43 0,91 4,27 69,63  Vanessa_cardui 0,69 0,00 2,43 0,59 3,02 72,65  Issoria_lathonia 0,51 0,00 2,39 0,59 2,97 75,62  Vanessa_atalanta 0,28 0,00 1,65 0,53 2,06 77,68  Cupido_argiades 0,35 0,00 1,61 0,52 2,01 79,69  Ochlodes_sylvanus 0,00 0,18 1,50 0,54 1,87 81,56  Pararge_aegeria 0,25 0,00 1,50 0,56 1,87 83,43  Thymelicus_lineola 0,25 0,00 1,18 0,41 1,47 84,90  Erynnis_tages 0,08 0,18 1,11 0,46 1,39 86,29  Thecla_betulae 0,15 0,00 1,06 0,48 1,32 87,61  Araschnia_levana 0,09 0,09 1,02 0,45 1,27 88,88  Aglais_io 0,15 0,00 1,00 0,34 1,25 90,13  Gonepteryx_rhamni 0,17 0,00 1,00 0,48 1,24 91,37  Polygonia_c-album 0,16 0,00 0,96 0,43 1,20 92,57  Aphantopus_hyperantus 0,27 0,00 0,90 0,39 1,12 93,69  Polyommatus_coridon 0,37 0,00 0,88 0,28 1,10 94,79  Lycaena_dispar 0,16 0,00 0,85 0,42 1,06 95,85 |
| --- |
