# Supplementary figures and images for: Life on Green Patches: Diversity and Seasonal Changes of Butterfly Communities Associated With Wastelands of the Post‐Industrial Central European City
Source: Ecol Evol. 2024 Dec 16;14(12):e70695. doi: 10.1002/ece3.70695 (PMC11650753; doi:10.1002/ece3.70695)

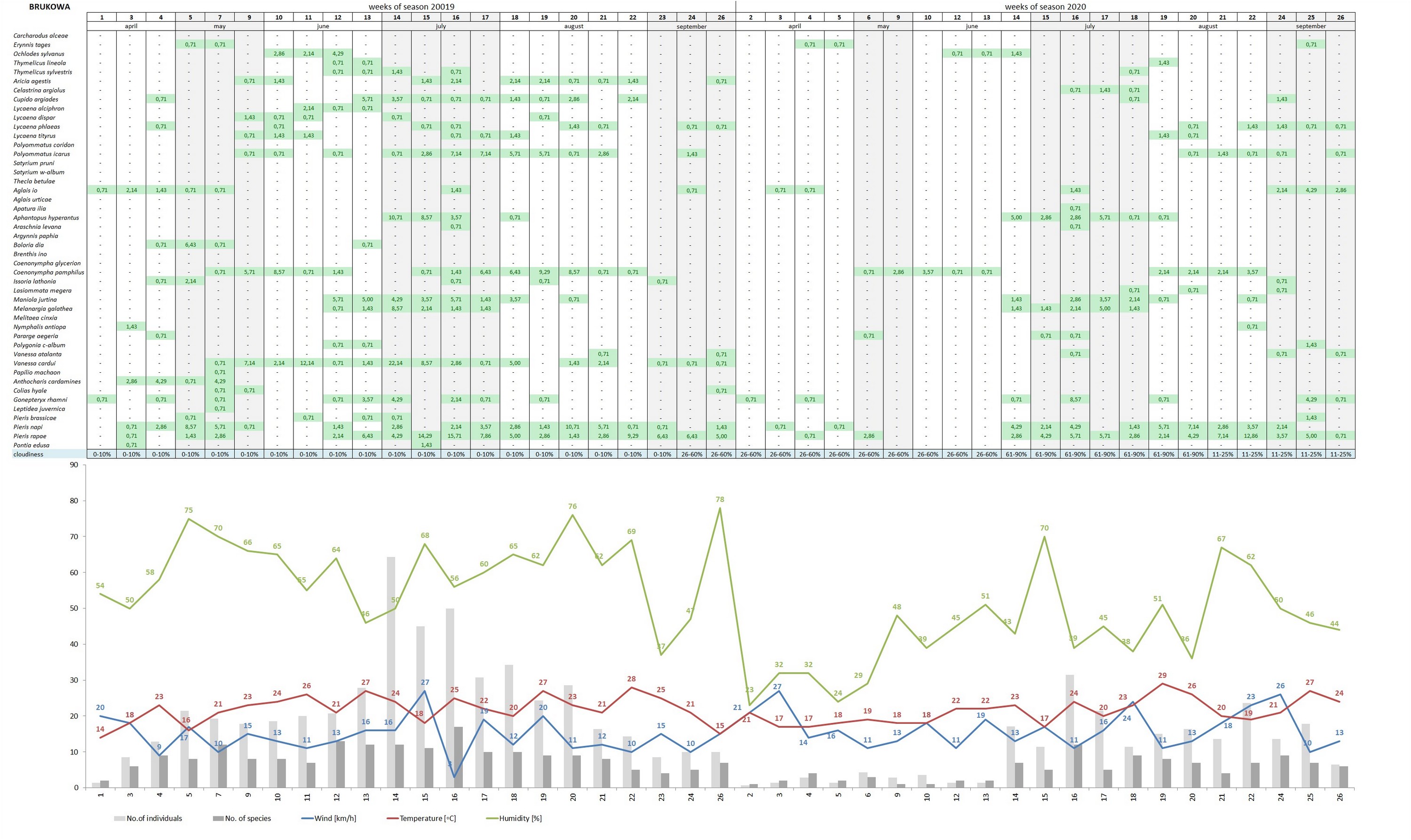

Supplement: Supplementary file 8 — Appendix S8. Seasonal changes of butterfly communities along 2019 and 2020 sampling seasons on Brukowa site on a background of weather conditions (humidity, temperature, cloudiness, wind speed). Abundance values per transect are given in the table. [file ECE3-14-e70695-s001.jpg]

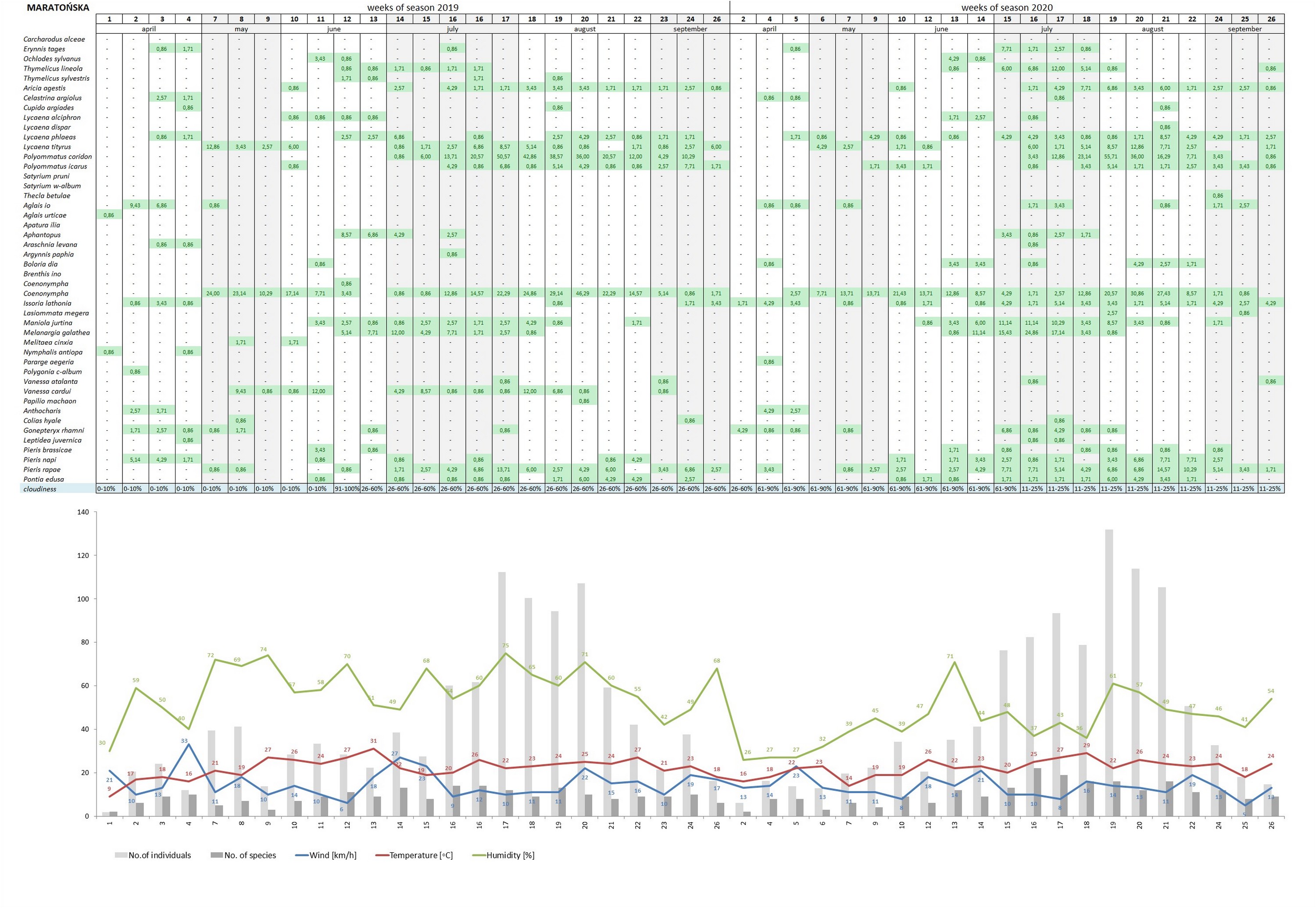

Supplement: Supplementary file 9 — Appendix S9. Seasonal changes of butterfly communities along 2019 and 2020 sampling seasons on Maratońska site on a background of weather conditions (humidity, temperature, cloudiness, wind speed). Abundance values per transect are given in the table. [file ECE3-14-e70695-s006.jpg]

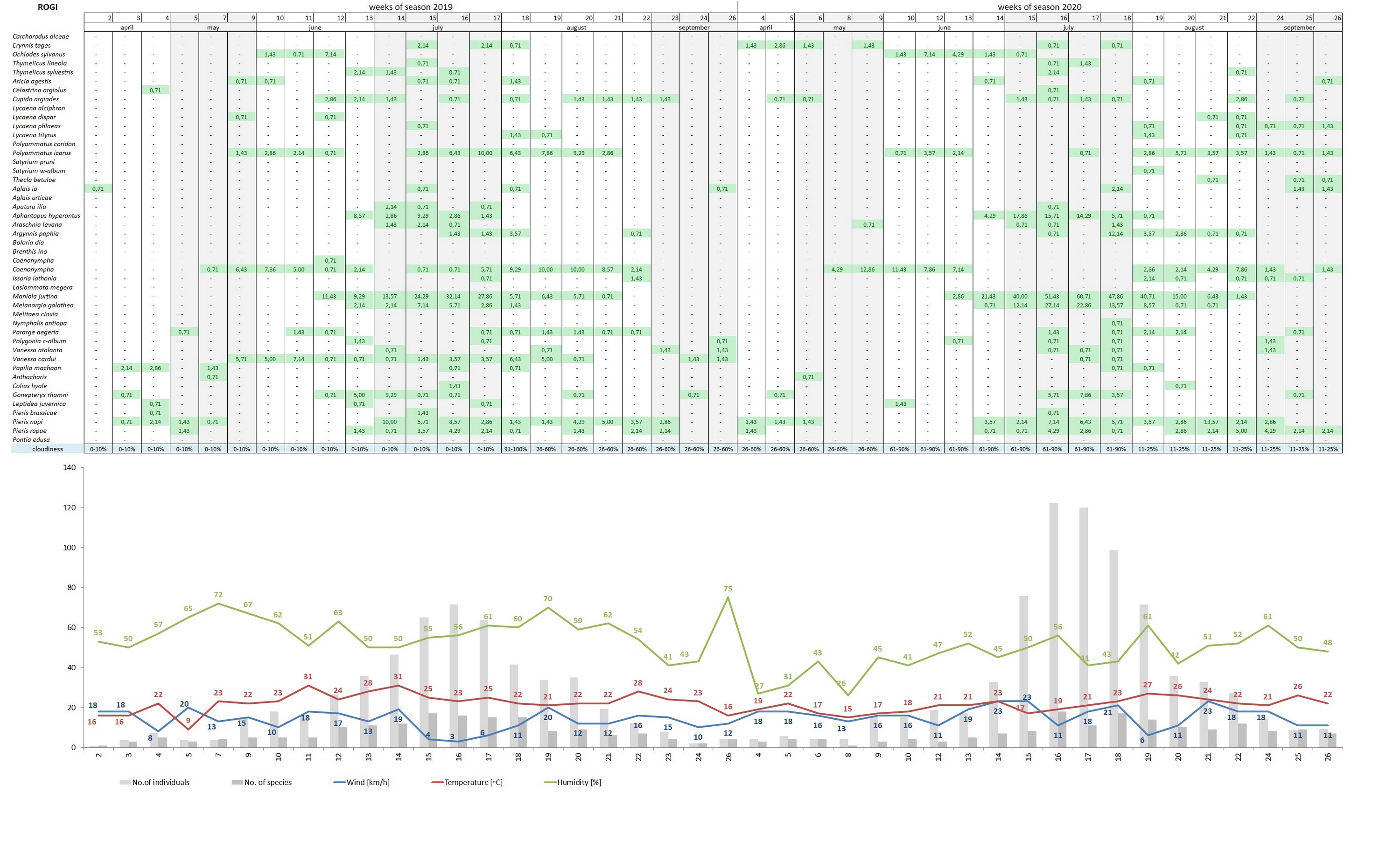

Supplement: Supplementary file 10 — Appendix S10. Seasonal changes of butterfly communities along 2019 and 2020 sampling seasons on Rogi site on a background of weather conditions (humidity, temperature, cloudiness, wind speed). Abundance values per transect are given in the table. [file ECE3-14-e70695-s003.jpg]

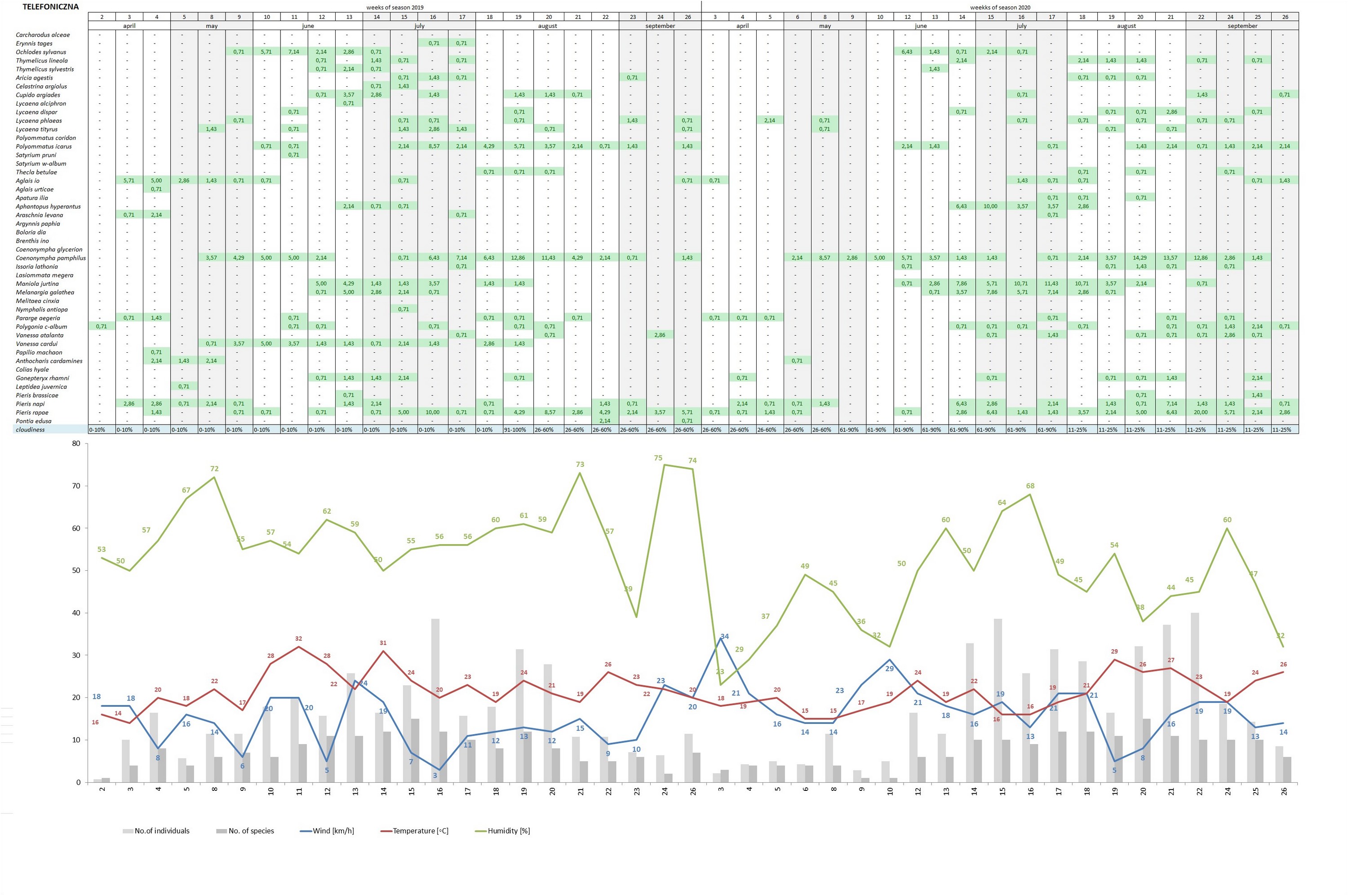

Supplement: Supplementary file 11 — Appendix S11. Seasonal changes of butterfly communities along 2019 and 2020 sampling seasons on Telefoniczna site on a background of weather conditions (humidity, temperature, cloudiness, wind speed). Abundance values per transect are given in the table. [file ECE3-14-e70695-s002.jpg]

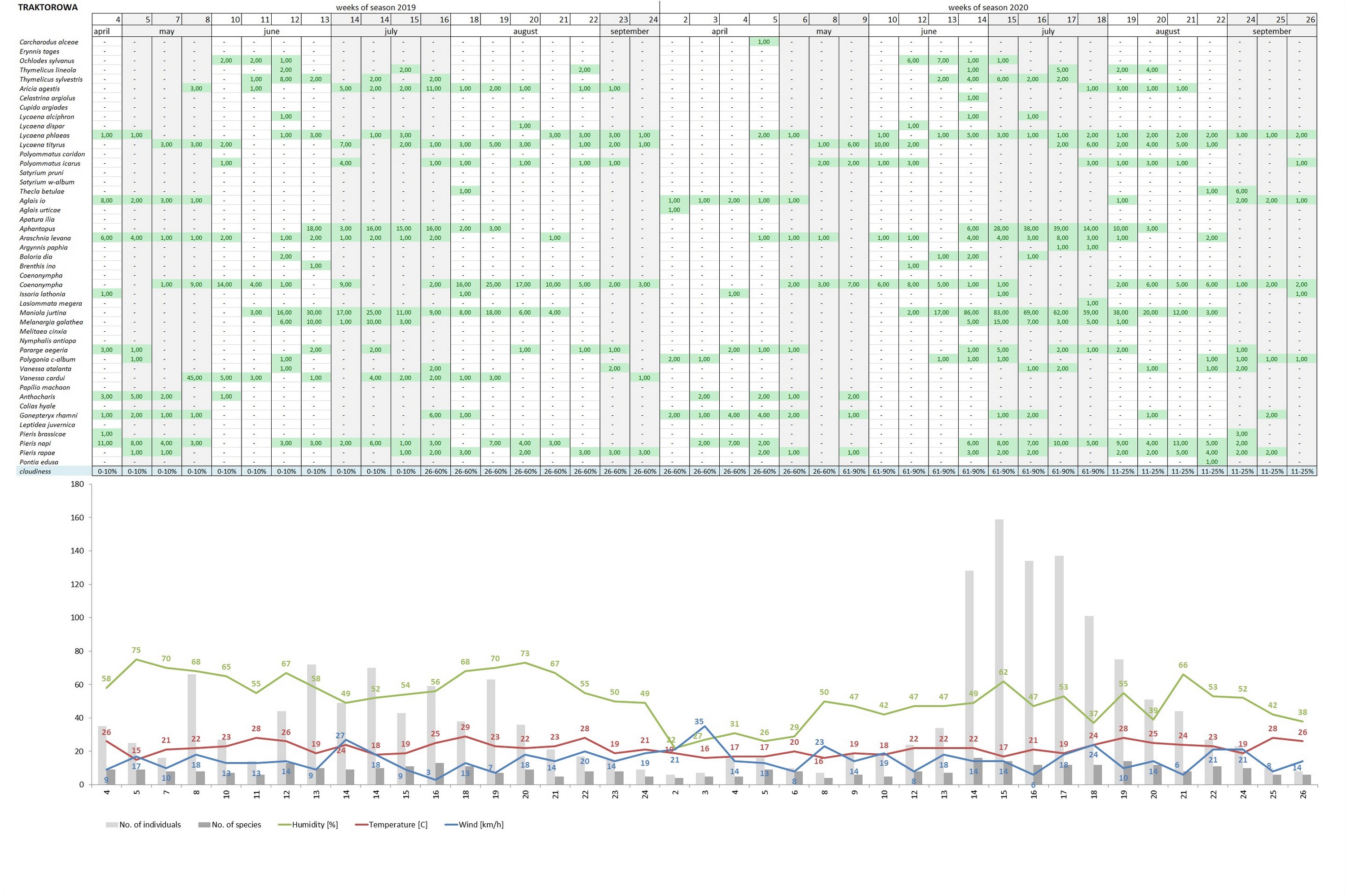

Supplement: Supplementary file 12 — Appendix S12. Seasonal changes of butterfly communities along 2019 and 2020 sampling seasons on Traktorowa site on a background of weather conditions (humidity, temperature, cloudiness, wind speed). Abundance values per transect are given in the table. [file ECE3-14-e70695-s013.jpg]
