# Supplementary material for: Life on Green Patches: Diversity and Seasonal Changes of Butterfly Communities Associated With Wastelands of the Post‐Industrial Central European City
Source: Ecol Evol. 2024 Dec 16;14(12):e70695. doi: 10.1002/ece3.70695 (PMC11650753; doi:10.1002/ece3.70695)
Supplement: Supplementary file 14 — Appendix S14. Samples from each dendrogram group. [file ECE3-14-e70695-s005.docx]

Appendix 14. Samples from each dendrogram group.

| Group 1 | 2020_12_B_07  2020_13_B_08  2020_09_R_05  2020_08_R_04  2020_10_TL_08  2020_10_B_06  2020_09_B_06  2020_09_TL_07 |
| --- | --- |
| Group 2 | 2019_21_TL_18  2020_06_B_05  2020_05_TL_04  2019_05_R_04  2020_04_TL_03  2019_22_R_17  2019_23_R_18  2019_24_TL_21  2019_21_B_18  2019_26_B_22  2019_22_B_19  2019_23_B_20 |
| Group 3 | 2020_13_M_09  2020_14_M_10  2019_12_TR_07  2020_13_TR_10  2020_18_R_12  2020_16_R_11  2020_17_R_12  2020_14_TR_10  2020_15_TR_11  2020_16_TR_12  2020_17_TR_13  2020_15_R_10  2019_13_TR_08  2019_14_TR_19  2019_15_R_11  2019_16_R_12  2020_19_R_13  2020_18_TR_14  2020_19_TR_15  2020_15_B_10  2020_17_B_12  2020_18_B_13  2020_17_TL_14  2019_14_R_10  2020_16_B_11  2020_16_TL_13  2020_18_TL_15  2020_14_TL_11  2020_15_TL_12  2020_14_B_09  2020_14_R_09  2019_12_M_10  2019_13_M_11  2019_13_TL_10  2019_14_TL_11  2019_13_B_10  2019_12_B_09  2019_12_TL_09  2019_16_TR_11  2019_13_R_09  2019_14_B_11 |
| Group 4 | 2019_16_M_14  2020_18_M_14  2020_19_M_15  2019_16_M_15  2019_17_M_16  2019_18_M_17  2019_19_M_18  2019_20_M_19  2020_22_M_18  2020_20_M_16  2020_21_M_17  2019_21_M_20  2019_22_M_21  2020_15_M_11  2020_16_M_12  2020_17_M_13  2019_15_M_13  2019_14_M_12  2019_15_TR_10  2019_15_TL_12  2019_15_B_12  2019_16_B_13 |
| Group 5 | 2019_24_TR_18  2019_23_M_22  2019_22_TR_16  2019_23_TR_17  2019_24_B_21  2019_26_TL_22  2020_09_M_06  2019_23_TL_20  2020_26_R_19  2019_24_M_23  2020_24_M_18  2020_25_M_19  2019_17_TL_14  2019_26_M_24  2020_26_M_20  2020_22_B_17  2020_20_B_15  2019_22_TL_19  2020_21_B_16  2020_20_TL_17  2020_22_R_16  2019_19_B_16  2020_10_M_07  2019_20_TL_17  2020_22_TL_19  2020_21_TL_18  2020_24_R_17  2020_24_TL_20  2020_19_B_14  2020_19_TL_16  2019_21_TR_15  2020_22_TR_18  2019_18_TR_12  2019_20_TR_14  2020_20_TR_16  2020_21_TR_17  2019_14_TR_09  2019_19_TR_13  2020_21_R_15  2019_17_R_12  2020_20_R_14  2019_20_B_17  2019_21_R_16  2019_18_TL_15  2019_19_R_14  2019_18_R_13  2019_16_TL_13  2019_18_B_15  2019_19_TL_16  2019_17_B_14  2019_20_R_15 |
| Group 6 | 2019_11_B_08  2019_11_M_09  2019_09_TL_06  2019_10_TL_07  2019_09_B_06  2019_11_R_21  2019_09_R_06  2019_10_R_07  2019_10_TR_05  2019_10_B_07  2019_11_TL_08  2019_11_TR_06  2020_13_TL_10  2020_12_M_08  2020_10_R_06  2020_12_TR_09  2020_13_R_08  2020_12_R_07  2020_12_TL_09 |
| Group 7 | 2019_04_M_04  2020_05_R_02  2020_06_R_03  2020_04_R_01  2020_05_B_04  2019_07_R_05  2020_06_TL_05  2019_03_R_02  2019_04_R_03  2020_03_B_02  2019_01_B_01  2019_02_R_01  2020_03_TL_02  2020_04_B_03  2020_26_TR_21  2020_25_R_18  2020_26_B_20  2020_24_B_18  2020_26_TL_22  2020_24_TR_19  2020_25_TL_21  2020_25_B_19  2020_25_TR_20  2019_03_B_02  2019_05_TL_04  2019_05_B_04  2019_07_B_05  2020_04_M_02  2019_03_M_03  2020_05_M_03  2019_03_TL_02  2019_04_TL_03  2020_05_TR_04  2020_06_TR_05  2019_02_M_02  2020_03_TR_02  2020_04_TR_03  2019_04_B_03  2019_04_TR_01  2019_05_TR_02 |
| Group 8 | 2020_02_M_01  2019_24_R_19  2020_02_B_01  2019_02_TL_01  2019_26_R_20  2020_02_TR_01 |
